# Drug‐microenvironment perturbations reveal resistance mechanisms and prognostic subgroups in CLL

**DOI:** 10.15252/msb.202110855

**Published:** 2022-08-12

**Authors:** Peter‐Martin Bruch, Holly AR Giles, Carolin Kolb, Sophie A Herbst, Tina Becirovic, Tobias Roider, Junyan Lu, Sebastian Scheinost, Lena Wagner, Jennifer Huellein, Ivan Berest, Mark Kriegsmann, Katharina Kriegsmann, Christiane Zgorzelski, Peter Dreger, Judith B Zaugg, Carsten Müller‐Tidow, Thorsten Zenz, Wolfgang Huber, Sascha Dietrich

**Affiliations:** ^1^ Department of Medicine V Heidelberg University Hospital Heidelberg Germany; ^2^ Molecular Medicine Partnership Unit (MMPU) Heidelberg Germany; ^3^ EMBL Heidelberg Heidelberg Germany; ^4^ Collaboration for Joint PhD Degree between EMBL and Heidelberg University Faculty of Biosciences Heidelberg Germany; ^5^ German Cancer Research Center (DKFZ) Heidelberg Germany; ^6^ National Center for Tumour Diseases Heidelberg Germany; ^7^ Institute of Pathology University of Heidelberg Heidelberg Germany; ^8^ Department of Hematology University of Zürich Zürich Switzerland

**Keywords:** chronic lymphocytic leukaemia, drug perturbation, microenvironment, multi‐omics, trisomy 12, Cancer, Computational Biology, Molecular Biology of Disease

## Abstract

The tumour microenvironment and genetic alterations collectively influence drug efficacy in cancer, but current evidence is limited and systematic analyses are lacking. Using chronic lymphocytic leukaemia (CLL) as a model disease, we investigated the influence of 17 microenvironmental stimuli on 12 drugs in 192 genetically characterised patient samples. Based on microenvironmental response, we identified four subgroups with distinct clinical outcomes beyond known prognostic markers. Response to multiple microenvironmental stimuli was amplified in trisomy 12 samples. Trisomy 12 was associated with a distinct epigenetic signature. Bromodomain inhibition reversed this epigenetic profile and could be used to target microenvironmental signalling in trisomy 12 CLL. We quantified the impact of microenvironmental stimuli on drug response and their dependence on genetic alterations, identifying interleukin 4 (IL4) and Toll‐like receptor (TLR) stimulation as the strongest actuators of drug resistance. IL4 and TLR signalling activity was increased in CLL‐infiltrated lymph nodes compared with healthy samples. High IL4 activity correlated with faster disease progression. The publicly available dataset can facilitate the investigation of cell‐extrinsic mechanisms of drug resistance and disease progression.

## Graphical abstract

Combined perturbation by microenvironmental stimuli and drugs of chronic lymphocytic leukaemia cells annotated for genetic alterations reveals distinct response patterns and molecular modulators.

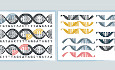

## Introduction

CLL is a common B‐cell malignancy, attributed to the accumulation of mature B‐lymphocytes in the peripheral blood, bone marrow and lymph nodes (Fabbri & Dalla‐Favera, 2016). The disease course is heterogeneous, influenced by multiple factors including B‐cell receptor (BCR) signalling, genetic alterations, epigenetic effects and the tumour microenvironment (Puente *et al*, 2018). Despite significant improvements to CLL treatment, including the advent of BCR inhibitors (Burger *et al*, 2015) and BH3‐mimetics (Fischer *et al*, 2019), CLL remains incurable. There is a compelling need to better understand cell‐intrinsic and extrinsic causes of therapy failure.

The genetic landscape of CLL is well characterised. The most important features include del(17p), *TP53*‐mutations, immunoglobulin heavy chain gene (IGHV) mutation status, del(13q) and trisomy 12 (Döhner *et al*, 2000; Gaidano & Rossi, 2017). This list of recurrent genetic and structural alterations has expanded in the past years (Strefford, 2015). The predictive value of these alterations has, however, declined in the context of modern targeted therapies, and resistance mechanisms are incompletely understood (Burger *et al*, 2020).

Additionally to cell‐intrinsic factors, CLL cell proliferation and survival is dependent on the lymph node microenvironment (Herndon *et al*, 2017), which is underlined by the observation that CLL cells undergo spontaneous apoptosis *in vitro* if deprived of protective microenvironmental signals (Collins *et al*, 1989). Microenvironmental stimuli can induce drug resistance *in vitro*. For example, the combination of IL2 and resiquimod, a Toll‐Like Receptor (TLR) 7/8 stimulus, induces resistance to venetoclax (Oppermann *et al*, 2016); and interferon‐γ (IFNγ) induces resistance to the BCR inhibitor ibrutinib (Xia *et al*, 2020). The CLL microenvironment constitutes a complex network of stromal and immune cells that promote cell expansion (Ten Hacken & Burger, 2016) via soluble factors and cell–cell contacts. Microenvironmental signalling is particularly important in protective niches, especially lymph nodes. Incomplete response to BCR inhibitors has been linked to persistent enlargement of lymph nodes (Ahn *et al*, 2018), which are the main site of CLL proliferation (Herndon *et al*, 2017).

Several studies have investigated individual components of the microenvironment in leukaemia (Dietrich *et al*, 2012; Chatzouli *et al*, 2014; Jayappa *et al*, 2017; Chen *et al*, 2019; preprint: Herbst *et al*, 2022a). However, systematic studies, particularly those exploring cell‐extrinsic influences on drug response, are rare. Carey *et al*, 2017 have screened acute myeloid leukaemia samples with a panel of soluble factors and amongst them identified IL1ß as a mediator of cellular expansion in acute myeloid leukaemia. This example highlights the value of systematic approaches to dissect tumour–microenvironment crosstalk.

Taking this approach further, we screened a panel of microenvironmental stimuli in CLL, individually and in combination with drugs, representing a reductionist approach to microenvironmental effects, and complemented our dataset with multi‐omics data on the patient samples. In this study, we integrate genetic, epigenetic and microenvironmental modulators of drug response in CLL systematically in a large patient cohort that covers the clinical and molecular diversity of CLL. Due to the dependency on microenvironmental support, CLL is an important model system for the interaction between malignant and microenvironmental cells in general.

## Results

### *Ex vivo* cell viability assay demonstrates functional diversity of cytokine and microenvironmental signalling pathways

**Figure 1:**
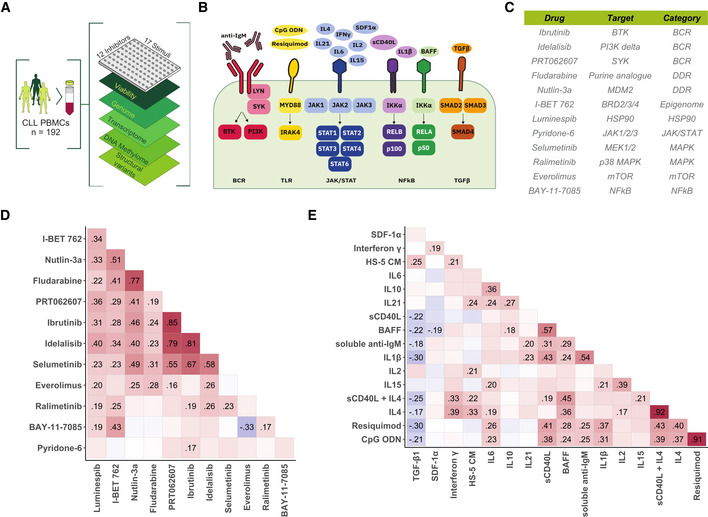
Study outline and overview of drugs and stimuli ASchematic of experimental protocol. We measured tumour cell viability after exposure to 12 drugs and 17 microenvironmental stimuli, both individually and in combination, in primary CLL samples (*n* = 192). Integrating these data with genomic, transcriptomic, DNA methylation and copy number variation data, we identified pro‐survival pathways, molecular modulators of drug and microenvironment responses, and drug‐stimulus interactions in CLL. The combinatorial data cube (12 × 17 × 192) can be accessed *in toto* (github.com/Huber‐group‐EMBL/CLLCytokineScreen2021) and explored interactively, via the shiny app (www.dietrichlab.de/CLL_Microenvironment/).BOverview of stimuli included in the screen and their primary annotated targets. In addition, we also used medium condition by the human stroma cell line HS‐5.CTable of drugs included in the screen. Drug target and category of target are also shown.D, EPearson correlation coefficients, taken across the 192 patient samples, of the logarithm of the relative viability after single treatment with drug (D) and stimulus (E) treatments. Text labels indicate correlation coefficients for those pairs where the correlation was significantly different from 0 at a false discovery rate (FDR) threshold of 0.05 (method of Benjamini & Hochberg (1995)), adjustment performed separately for drug and stimulus correlations).

To investigate the influence of 17 cytokines and microenvironmental stimuli on spontaneous and drug‐induced apoptosis, we measured their effects on cell viability in 192 primary CLL samples individually, as well as in combination with each of 12 drugs (Fig 1A–C). Relative viability was measured as luminescence‐based ATP count (i.e. Celltiter‐Glo, Promega®) after 48 h of drug‐stimulus perturbation divided by the median of the DMSO controls. In subsequent analyses, we used the natural logarithm of this value, so that negative and positive values indicate pro‐ and antiapoptotic effects, respectively, and values close to 0 correspond to no effect.

The patient samples were characterised by DNA sequencing, mapping of copy number variants, genome‐wide DNA methylation profiling and RNA‐Sequencing (Dietrich *et al*, 2018). The distribution of genetic features in our cohort is comparable to other studies (Appendix Fig S1 and Table S1) (Döhner *et al*, 2000).

To assess the heterogeneity of the response patterns, we calculated Pearson correlation coefficients for each pair of drugs and each pair of stimuli. For the drugs, high correlation coefficients (*R* > 0.75) were associated with identical target pathways. For example, drugs targeting the BCR pathway (ibrutinib, idelalisib, PRT062607 and selumetinib) were highly correlated, indicating that our data sensitively and specifically reflect inter‐individual differences in pathway dependencies (Fig 1D) (Dietrich *et al*, 2018).

In contrast, microenvironmental stimuli showed lower correlations, even where stimuli targeted similar pathways (Fig 1E). For example, lower correlations were observed between different stimuli of the JAK–STAT and NfκB pathways, indicating a low degree of redundancy between stimuli. High correlations (*R* > 0.75) were only seen with the TLR stimuli resiquimod (TLR7/8) and CpG ODN (TLR9) as well as IL4 and IL4 + soluble CD40L (sCD40L), which target near‐identical receptors and downstream targets.

Most stimuli increased CLL viability, underlining the supportive nature of the microenvironment. However, IL6 and tumour growth factor β (TGFβ) decreased viability (Appendix Fig S2). The strongest responses were seen with IL4 and TLR7/8/9 agonists, highlighting the potency of these pathways in modulating CLL survival. All screening data can be explored manually (github.com/Huber‐group‐EMBL/CLLCytokineScreen2021) and interactively, via the shiny app (dietrichlab.de/CLL_Microenvironment/).

### Patterns of responses to microenvironmental stimuli reveal two subgroups of IGHV‐M CLL with differential aggressiveness

**Figure 2:**
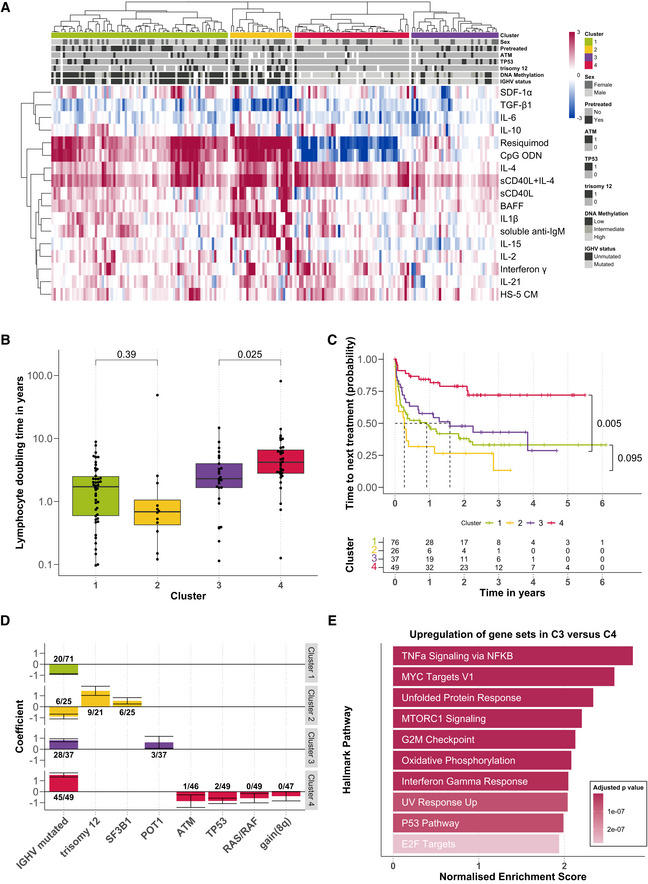
*Ex vivo* microenvironmental response profiling reveals subgroups with distinct molecular and clinical profiles Heatmap showing viability measurements for the 192 samples (rows) subject to each of 17 microenvironmental stimuli (columns). The data are shown on a robust *z*‐score scale, that is, the logarithm of the relative viability measurements was scaled by the median absolute deviation within each row. Limits were applied to scaling factor for optimal visualisation. Red indicates increased viability, and blue indicates decreased viability. Samples are annotated for genetic features, sex and pre‐treatment status.Lymphocyte doubling time, a clinical marker for disease progression, plotted for each cluster, within the subset of 115 for which this measurement was available (C1: *n* = 46; C2: *n* = 12; C3: *n* = 25; C4: *n* = 32). *P*‐values from Student's *t*‐test (non‐paired, two‐sided). The central bar, boxes and whiskers of the plot represent the median, first and third quartiles, and 1.5‐time interquartile range (IQR), respectively.Clinical outcome. Kaplan–Meier curves of time to next treatment for each cluster. *P*‐values from univariate Cox proportional hazard models comparing C1 with C2, and C3 with C4. Survival data were available for 188 patient samples.Genetics. Multinomial regression quantifies association of genetic features with each cluster. X‐axis indicates genetic features, and y‐axis indicates coefficient from multinomial regression, indicating enrichment or depletion of the feature within each cluster. Text labels show the number of positive cases and the total number of cases tested for the feature, in each cluster. Error bars represent mean ± standard deviation.Transcriptomes. GSEA comparing the expression of genes in samples from C3 (*n* = 9) versus those C4 (*n* = 12). Normalised enrichment scores are shown for the top 10 most significant Hallmark gene sets (Liberzon *et al*, 2015).

To gain a global overview of the pattern of responses to our set of microenvironmental stimuli across patient samples, we visualised and clustered (Wilkerson & Hayes, 2010) the stimulus–response profiles of all samples (Fig 2A).

This analysis revealed four functionally defined subgroups with distinct stimulus–response profiles. Two clusters (termed C1 and C2) were enriched in IGHV‐unmutated CLLs (IGHV‐U), and two (C3 and C4) in IGHV‐mutated CLLs (IGHV‐M). C1 and C2 showed strong responses to IL4 and TLR7/8/9 agonists. C2 was distinguished by stronger responses to the stimuli overall, in particular to NFκB agonists including IL1β and anti‐IgM. C3 responded weakly to the majority of stimuli, whilst C4 was defined by a decreased viability upon TLR7/8/9 stimulation (Appendix Fig S3).

Next, we investigated whether these clusters were associated with differential *in vivo* disease progression of the corresponding patients. We used lymphocyte doubling time (available for *n* = 115 samples), time to next treatment (*n* = 188) and CLL Proliferative Drive (CLL‐PD, *n* = 146) to quantify clinical progression dynamics and proliferative capacity of the CLL cells. In line with clinical observations (Baumann *et al*, 2021), the IGHV‐U‐enriched C1 and C2 showed a shorter lymphocyte doubling time than the IGHV‐M‐enriched C3 and C4. More strikingly, C3 showed a significantly shorter lymphocyte doubling time than C4, indicating a higher proliferative capacity (Fig 2B).

This observation was reflected in disease progression rates: the predominantly IGHV‐M C3 showed a progression dynamic similar to the IGHV‐U‐enriched C1 and C2. C4, however, showed a longer time to next treatment than C1, C2 and C3 (Cox proportional hazards model, *P* = 0.0014, < 0.001, 0.005, respectively, Fig 2C, Appendix Tables S2 and S3). C3 had significantly shorter time to first treatment than C4 (*P*‐value < 0.001, Appendix Fig S4A). Overall survival was not detectably different between C3 and C4 (Appendix Fig S4B). This points towards a subset of mostly IGHV‐M patients with more aggressive disease (C3), which is characterised by its differential response to microenvironmental stimuli.

CLL Proliferative Drive (CLL‐PD) is a recently discovered molecular biomarker profile that captures the proliferative capacity of CLL cells based on data from multiple omic layers (Lu *et al*, 2021). CLL‐PD was significantly higher in C3 samples than in C4 and higher in C2 samples than in C1 (Appendix Fig S5A). To understand these relationships, we looked for associations between CLL‐PD and the response to each stimulus, using multivariate regression (Appendix Fig S5B). CLL‐PD was associated with distinct pathways in IGHV‐M and U disease. In IGHV‐M, CLL‐PD correlated with responses to TLR stimuli (Appendix Fig S5C and E). In IGHV‐U, it correlated with NFkB and TGF‐beta stimuli (sCD40L, IL‐1β, soluble anti‐IgM and TGF‐β1, Appendix Fig S5D and F).

We next aimed to characterise molecular differences between clusters. We used multinomial regression to identify associations between genetic features and cluster assignment (Fig 2D). The model assigned positive and negative coefficients to genetic features associated with each cluster, indicating that the feature was enriched or depleted. Unmutated IGHV was associated with C1 and C2, whilst mutated IGHV was associated with clusters C3 and C4. Trisomy 12 and *SF3B1* mutations were associated with C2, which showed enhanced responses to many stimuli. *POT1* mutations were enriched in C3, which may contribute to the worse survival of C3 patients. *TP53*, *ATM*, *RAS*/*RAF* mutations and gain(8q) were depleted in C4, which was associated with slow *in vivo* progression.

In addition to genetic features, we also investigated the epigenetic profiles of the clusters. We observed that C1 and C2 were enriched for samples with low programmed (LP) DNA methylation profiles, whilst C3 and C4 contained many samples with high programmed (HP) DNA methylation profiles (Appendix Fig S6) (Oakes *et al*, 2016).

Notably, the observed difference in progression dynamics *in vivo* was not explained solely by the known prognostic markers IGHV status, trisomy 12, *TP53*, DNA methylation profiles and *POT1* (Ramsay *et al*, 2013). A multivariate Cox proportional hazards model accounting for these features showed an independent prognostic value of the cluster assignment between C3 and C4 (*P* = 0.03, Appendix Table S4).

To examine why C3 exhibited faster disease progression than C4, we compared baseline pathway activity at the time of sampling, using RNA‐Sequencing (Appendix Fig S7A). GSEA revealed that key cytokine gene sets were upregulated in C3, including TNFα signalling via NFκB, indicating higher pathway activity *in vivo* (Fig 2E; Appendix Fig S7B). In addition, pathways that indicate more aggressive disease relating to proliferation, metabolism and stress response were upregulated in C3 (Appendix Fig S7C–E). Differential gene expression between C1 and C2 is shown in Appendix Fig S8.

The clusters also demonstrated differential pathway sensitivities. For example, we found C3 to be more sensitive towards treatment with BAY‐11‐7085, a selective IκBα phosphorylation inhibitor (Appendix Fig S9 and S10). In C3 samples, the treatment with BAY‐11‐7085 led to a reduction of the pro‐survival effect of BAFF, which stimulates the NFκB pathway (Appendix Fig S11).

Taken together, the patterns of responses to our set of stimuli distinguish two subgroups of IGHV‐M CLL (C3 and C4) that are characterised by distinct *in vivo* pathway activities and differential disease progression.

### Microenvironmental signals and genetic features collectively determine malignant cell survival

**Figure 3:**
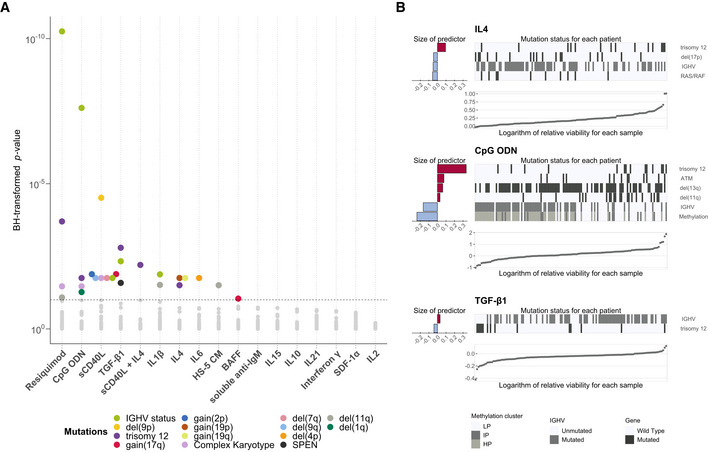
Responses to microenvironmental signals are modulated by recurrent genetic features in CLL Overview of genetic feature‐stimulus associations. *x*‐axis shows stimuli, and y‐axis shows *P*‐values from Student's *t*‐tests (two‐sided, equal variance). Each dot represents a genetic feature‐stimulus association. Tests with *P*‐values smaller than the threshold corresponding to an FDR of 10% (method of Benjamini & Hochberg (1995)) are indicated by coloured circles, where the colours represent the gene mutations and structural aberrations. All genetic features tested can be found in Appendix Table S1.Predictor profiles depicting genetic feature‐stimulus associations identified through Gaussian linear modelling with L1‐penalty, for selected stimuli. The bar plots on the left indicate size and sign of coefficients assigned to genetic features that are associated with response to given stimulus. Positive coefficients indicate higher viability after stimulation if the feature is present. The heatmaps show mutation status in each of the patient samples. The samples were sorted by the logarithm of the relative viability, which is shown in the scatter plots at the bottom. In total, 39 genetic alterations were tested amongst 129 patient samples with complete annotation. LP, low programmed; IP, intermediate programmed; HP, high programmed (Oakes *et al*, 2016).

Having observed heterogeneous responses to stimulation, we performed univariate analyses of genetic determinants of stimulus–response, including IGHV status, somatic gene mutations and structural variants (Fig 3A). At least one genetic feature determined stimulus–response for 10/17 stimuli and at least two features for 6/17 stimuli (Student's *t*‐tests, FDR = 10%), indicating that CLL viability is controlled both by cell‐intrinsic genetic alterations and the cells' microenvironment. We excluded genetic features with less than three positive cases in our cohort as associations with rare events are less reliable (Appendix Table S1). The most prominent features affecting stimulus–response were IGHV status and trisomy 12. Responses to microenvironmental stimuli were largely independent of receptor expression (Appendix Fig S12).

To address the possible interplay of multiple genetic features, we applied linear regression with lasso regularisation to derive a multivariate predictor for each stimulus, composed of genetic, IGHV and DNA methylation covariates (Fig 3B; Appendix Fig S13). This revealed at least one genetic predictor for five of the 17 stimuli. Trisomy 12 and IGHV status were again the most common features. Stimulus–responses stratified by mutations, along with regression model fits, can be explored online (dietrichlab.de/CLL_Microenvironment/).

### Trisomy 12 is a key modulator of responses to stimuli

Our survey of genetic determinants of stimulus–response highlighted trisomy 12 as a modulator of responses to IL4, TGFβ, soluble CD40L + IL4 and TLR stimuli.

**Figure 4:**
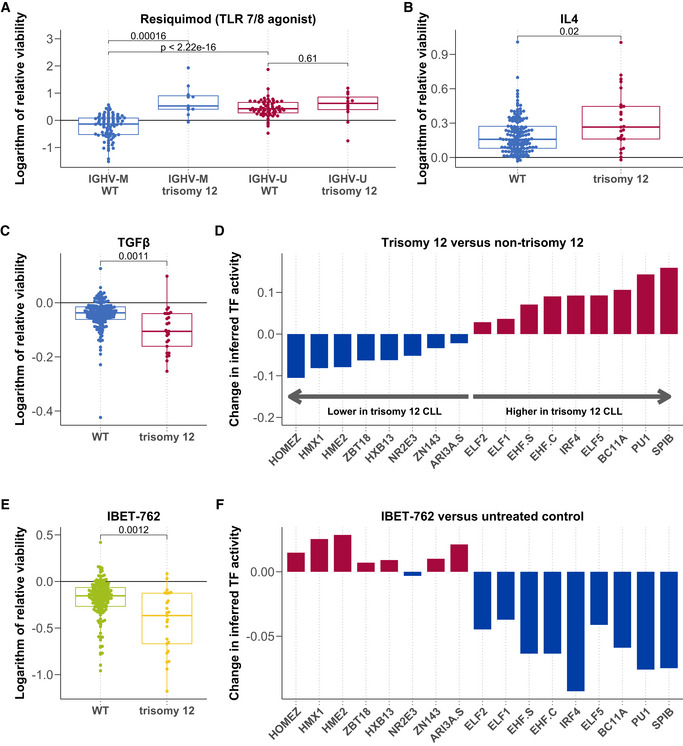
Trisomy 12 modulates responses to microenvironmental signals A–CLogarithm of the relative viability after treatment with Resiquimod (A, *n* = 169), IL4 (B, *n* = 174) and TGFβ (C, *n* = 174) stratified by trisomy 12, and in (A), also by IGHV status. *P*‐values from Student's *t*‐test (two‐sided, non‐paired). The central bar, boxes and whiskers of the plot represent the median, first and third quartiles, and 1.5‐time IQR, respectively.DChange in inferred TF activity (*y*‐axis) between samples with (*n* = 9) and without (*n* = 43) trisomy 12. Plot shows a change in inferred activity for all TFs with an adjusted *P*‐value < 0.05. Changes in inferred activity and *P*‐values for 638 TFs calculated using the diffTF software and data taken from (Rendeiro *et al*, 2016).ENatural logarithm of the relative viability (*n* = 174) after treatment with IBET 762 in trisomy 12 and non‐trisomy 12 CLL samples. *P*‐value from Student's *t*‐test (two‐sided, non‐paired). The central bar, boxes and whiskers of the plot represent the median, first and third quartiles, and 1.5‐time IQR, respectively.FDifferential inferred TF activity (y‐axis) comparing IBET 762 (*n* = 4) and control‐treated CLL samples (*n* = 4). Plot shows inferred activity for the same TFs shown in Fig 4D.

TLR response has previously been shown to depend on the IGHV mutation status (Chatzouli *et al*, 2014); we identified trisomy 12 as a second major determinant of response to TLR stimulation. IGHV‐mutated CLL samples with trisomy 12 showed a strongly increased viability after TLR stimulation with resiquimod compared to those without trisomy 12. IGHV‐unmutated CLL cells showed a strong increase in viability upon TLR stimulation regardless of trisomy 12 status (Fig 4A). In addition, we observed that the presence of trisomy 12 enhanced the effects of IL4 and TGFβ stimulation (Fig 4B and C).

We hypothesised that modulation of response to the microenvironment may play a role in the mechanism of trisomy 12 pathogenicity, which is so far incompletely understood. Previous work has indicated that trisomy 12 CLLs show higher transcript and protein abundance of microenvironmental pathways including BCR (preprint: Herbst *et al*, 2022b) and chemokine signalling genes (Dietrich *et al*, 2018).

Building on these studies of the trisomy 12 CLL transcriptome and proteome, we investigated the impact on the epigenome. We used chromosome accessibility data from ATAC sequencing to infer transcription factor (TF) activity based on motif accessibility. We quantified inferred TF activity in trisomy 12 and non‐trisomy 12 CLL PBMC samples (*n* = 4). 92 TFs demonstrated higher inferred activity (*P* < 0.05) in the trisomy 12 samples (Appendix Fig S14), reflecting a specific signalling signature in trisomy 12 CLL. Spi‐B and PU.1 were the top hits. Both are haematopoietic regulators, which exhibit functional redundancy (Garrett‐Sinha *et al*, 2001). They also have similar binding motifs, which makes it difficult to distinguish whether either or both are upregulated in trisomy 12 based on this motif‐based method of inferring activity.

We validated our finding in two additional independent datasets taken from Rendeiro *et al*, 2016 (nine trisomy 12 and 43 non‐trisomy 12) and Beekman *et al*, 2018 (13 trisomy 12 and 87 non‐trisomy 12). In the Rendeiro *et al*, 2016 data, nine TFs showed higher inferred activity (*P* < 0.05) in the trisomy 12 samples (Fig 4D) and in the Beekman *et al*, 2018 dataset, 18 TFs showed higher inferred activity (*P* < 0.05) in the trisomy 12 samples (Appendix Fig S15). Spi‐B and PU.1 were the top hits in both datasets.

To check whether the results were affected by the additional reads on chromosome 12, we repeated these analyses without including data from chromosome 12 (Appendix Fig S16) and Spi‐B and PU.1 remained the top hits.

Spi‐B and PU.1 are known to be key regulators of healthy B‐cell function (Ray‐Gallet *et al*, 1995; Turkistany & DeKoter, 2011), controlling B‐cell responses to environmental cues including CD40L, TLR ligands and IL4 (Willis *et al*, 2017). We hypothesised that these TFs might modulate proliferation by coordinating transcriptional responses to microenvironmental signals in trisomy 12 CLL.

To investigate this, we first visualised the genomic locations of all Spi‐B and PU.1 motifs that showed differential inferred activity in trisomy 12 samples (Appendix Fig S17). This analysis indicated that Spi‐B and PU.1 binding motifs showing differential accessibility in trisomy 12 CLL are distributed throughout the genome. We next approximated Spi‐B and PU.1 target genes in trisomy 12 CLL by annotating each of these motif locations with the nearest gene. We ran over‐representation tests to investigate the enrichment of immune and control pathways amongst these gene targets. We found that JAK–STAT signalling was enriched amongst Spi‐B targets and TCR signalling genes were enriched amongst PU.1 targets (*P* < 0.01, Appendix Table S5).

To further validate our hypothesis, we additionally determined Spi‐B and PU.1 target genes through the use of a ChIPseq dataset (Care *et al*, 2014), quantifying Spi‐B and PU.1 binding in lymphoma cell lines. We found that TLR, BCR and TGFβ signalling genes were enriched (*P* < 0.01) amongst the Spi‐B targets based on ChIPseq data (Appendix Table S6). No pathways were enriched amongst the PU.1 targets. Whilst lymphoma cell lines cannot completely capture the biology of CLL, this result supports the hypothesis that Spi‐B may control microenvironmental response genes in malignant B‐cells.

Finally, we investigated whether Spi‐B and PU.1 activity and the trisomy 12 TF signature might be targetable. Amongst the panel of drugs included in this screen, we noted that the bromodomain inhibitor IBET‐762 demonstrated higher efficacy in trisomy 12 CLL cells (Fig 4E). We generated an additional ATACseq dataset consisting of four CLL PBMC samples each treated with IBET‐762 and DMSO as control. We visualised inferred TF activity after IBET‐762 treatment, for trisomy 12 signature TFs. The inferred TF activity was decreased upon IBET‐762 treatment in a broad range of TFs, including all nine TFs with higher inferred activity in trisomy 12 CLL (Fig 4F).

Taken together, we highlight trisomy 12 as a modulator of response to microenvironmental signals, describe increased Spi‐B and PU.1 TF activity as a potential effector mechanism and link Spi‐B and PU.1 to microenvironmental signalling genes in CLL and lymphoma. BET inhibition, which has previously been suggested as a therapeutic target in CLL (Ozer *et al*, 2018), could therefore be a promising therapeutic strategy in trisomy 12 patients.

### Mapping drug‐microenvironment interactions reveals drug resistance and sensitivity pathways

Guided by the observation of reduced treatment efficacy in protective niches (Ahn *et al*, 2018), we examined the effects of the stimuli on drug response.

We fitted a linear model (Equation 1) to quantify how each stimulus modulates drug efficacy beyond its individual effects, such as the impact of the stimulus on baseline viability and spontaneous apoptosis. *β*_int_, termed the interaction factor, quantifies how the combined treatment effect differs from the sum of the individual treatment effects.

(1) $$\log V=\beta_{d}X_{d}+\beta_{s}X_{s}+\beta_{\mathrm{int}}X_{d}X_{s}+\varepsilon$$

*V* represents the viability with a given treatment, *β*_d_, *β*_s_, and *β*_int_ are coefficients for the drug, stimulus, and combinatorial terms, respectively, *X*_*d*_ and *X*_*s*_ are indicator variables (0 or 1) for the presence or absence of a drug/stimulus. *ε* is the vector of model residuals.

**Figure 5:**
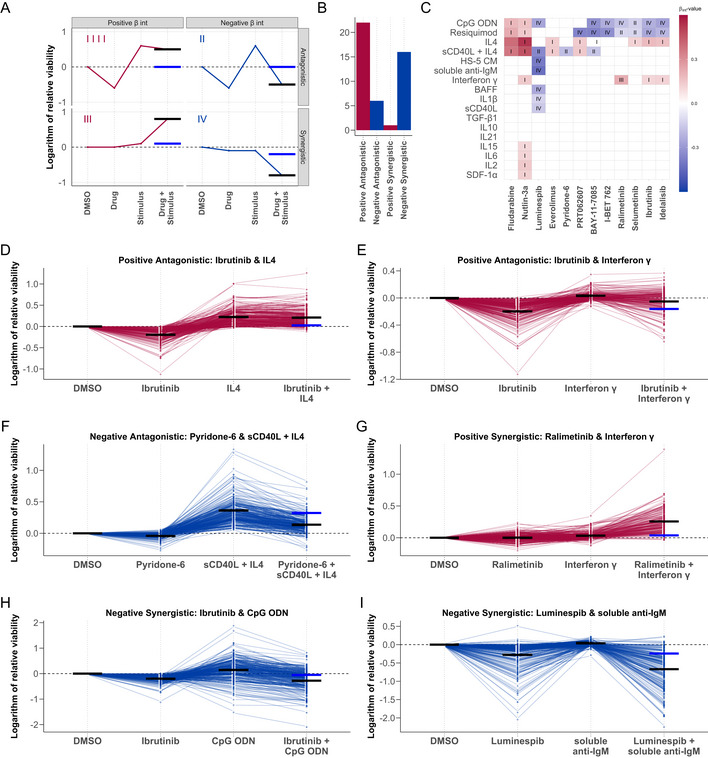
Microenvironmental stimuli influence *ex vivo* drug response AGraphical representation of the four drug‐stimulus interaction categories. Categories are defined according to the nature of the interaction (synergistic or antagonistic), and whether the viability is increased or decreased by the stimulus (positive or negative). x‐axis shows treatment type, and y‐axis shows viability with each treatment. Red and blue points and lines depict a representative treatment response pattern for given interaction type. Blue horizontal lines represent the expected viability for combinatorial treatment in the absence of an interaction between drug and stimulus (i.e. additive effects), and black horizontal lines represent the measured viability after combinatorial treatment. The difference between the black and blue lines indicates the impact of the interaction on expected viability.BBar plot of the number of interactions in each category where the *P*‐value for the interaction term *β*_int_ < 0.05.CHeatmap of all *β*_int_ values with associated *P*‐value < 0.05, annotated with interaction category (I‐IV). Rows and columns indicate drugs and stimuli, ordered according to hierarchical clustering. Scale indicates size and sign of *β*_int_ for given drug‐stimulus combination. (I) Positive *β*_int_ and antagonistic (microenvironmental stimulation reduces drug effect). (II) Negative *β*_int_ and antagonistic (drug reduces stimuli effect). (III) Positive *β*_int_ and synergistic (microenvironmental stimulation and drug have synergistic pro‐survival effect). (IV) Negative *β*_int_ and antagonistic (microenvironmental stimulation and drug show synergistic toxicity).D–IExamples of drug‐stimulus interactions, for each category. Plots show the natural logarithm of the relative viability of 192 CLL samples after treatment with the respective drug and stimulus. Each line represents one patient sample linked across treatments. Black horizontal lines in each single treatment indicate the expected viability based on linear modelling. Black and blue horizontal lines in the combinatorial treatment indicate the expected viability based on the additive effect of the drug and stimulus (blue), and the expected viability accounting for the additive effect and the interaction (black). (D + E) Ibrutinib, a clinically used BTK inhibitor, is blocked by IL4 and IFNγ. (F) The JAK inhibitor pyridone‐6 inhibits the pro‐survival effect of sCD40L + IL4 stimulation. (G) The p38 inhibitor ralimetinib and IFNγ show a synergistic pro‐survival effect not observed in either single treatment. (H) TLR agonists, including CpG ODN (shown), increase sensitivity to BTK inhibition by ibrutinib, despite increasing viability as single treatments. (I) Soluble anti‐IgM sensitises CLL samples to HSP90 inhibition by luminespib.

Forty‐five out of 204 combinations had a *β*_int_ with *P* < 0.05. We classified these into four categories, based on the sign of *β*_int_ and whether the combination effect was antagonistic or synergistic (Fig 5A–C).

Positive antagonistic interactions were the most common category, in which stimuli reversed drug action and increased viability. These interactions could lead to treatment resistance *in vivo*. For example, multiple stimuli reduced the toxicity of the chemotherapeutic compounds fludarabine and nutlin‐3a. The action of targeted therapies (including BCR inhibitors) was also reduced by the stimuli, most commonly by IL4 (Fig 5D) and IFNγ (Fig 5E), in alignment with other studies on IL4 and IFNγ (Aguilar‐Hernandez *et al*, 2016; Xia *et al*, 2020). IL4 and IFNγ reversed drug action in a common set of drugs, suggesting that both stimuli may act via a common mechanism.

A total of 6/45 interactions were categorised as negative antagonistic, in which drug action reversed the pro‐survival effect of microenvironmental stimulation. These interactions point to strategies to overcome treatment resistance. For instance, pan‐JAK inhibition reduced the increase in viability from sCD40L + IL4 stimulation (Fig 5F).

We observed a single positive synergistic case, whereby simultaneous treatment with IFNγ and ralimetinib, a p38 MAPK inhibitor, induced a synergistic increase in viability which was not observed with either single treatment (Fig 5G), pointing towards an inhibitory effect of p38 activity on IFNγ signalling.

Sixteen combinations showed negative synergistic interactions. For example, TLR9 agonist CpG ODN increased the efficacy of BCR inhibition (Fig 5H). Interestingly, in samples where TLR stimulation increased viability, the addition of BCR inhibitors (ibrutinib and idelalisib) suppressed this effect, indicating that the increase in viability upon TLR stimulation is dependent on BCR activity. The efficacy of luminespib, a HSP90 inhibitor, increased with various stimuli, including soluble anti‐IgM (Fig 5I).

Altogether, these results highlight the influence of the microenvironment on drug efficacy and underline the value of microenvironmental stimulation in *ex vivo* studies of drug efficacy.

### Genetic features affect drug‐microenvironment interactions

Next, to investigate to what extent genetic driver mutations modulated the interactions between stimuli and drugs, we fit the linear model in Equation (1) in a sample‐specific manner.

**Figure 6:**
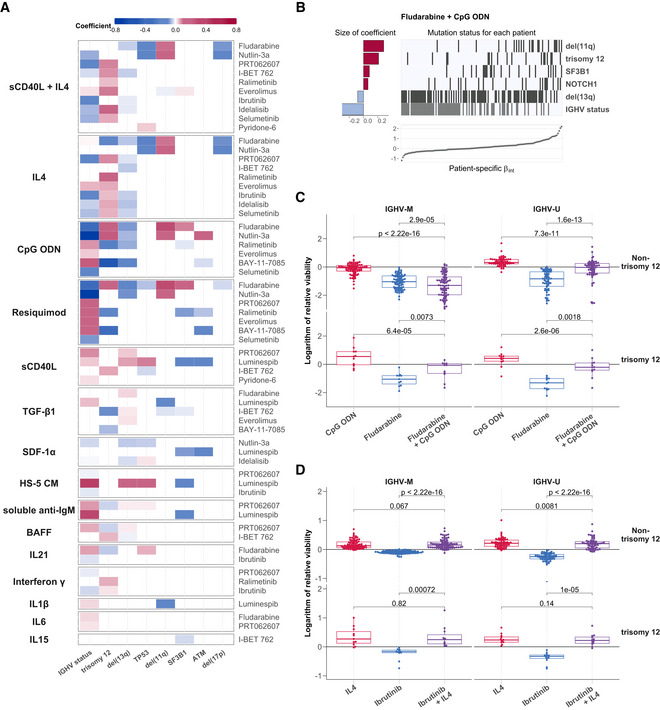
Integrating the effects of genetic features and stimuli on drug response Based on the drug‐stimulus viability data of 137 CLL patient samples with complete genetic annotation, we fitted a sample‐specific linear model.
AHeatmap depicting genetic predictors of drug‐stimulus interactions. Each row indicates genetic features that are associated with the size of interaction between the drug (right) and stimulus (left), that is, each row depicts the coefficients of a single multivariate model fit as in (B)). Coloured fields indicate that *β*_int_ for given drug and stimulus is modulated by corresponding genetic feature. Positive coefficients are shown in red, indicating a more positive *β*_int_ if the feature is present. For clarity, plot shows eight most common genetic features and omits drug‐stimulus combinations where no coefficient was assigned to any of these eight features. Heatmap of coefficients for all tested genetic features can be seen in Appendix Fig S18.BPredictor profile depicting genetic features that modulate the interaction between fludarabine and CpG ODN, identified using multivariate modelling. The horizontal bars on the left show the size of coefficients assigned to genetic features by model. The matrix in the centre indicates patient sample mutation status for the given genetic features aligned with the scatter plot below indicating the size of *β*_int_ for the same patient sample. Grey lines indicate the presence of genetic feature/IGHV mutated.C, DBeeswarm boxplots of the natural logarithm of the relative viability of 169 CLL samples, for (C) fludarabine + CpG ODN and (D) ibrutinib + IL4 single and combinatorial treatments, faceted by IGHV status and trisomy 12 status. *P*‐values from paired Student's *t*‐test. The central bar, boxes and whiskers of the plot represent the median, first and third quartiles and 1.5‐time IQR, respectively.

This resulted in a sample‐specific *β*_int_ interaction coefficient for each drug‐stimulus combination. We looked for associations between the size of *β*_int_ and genetic features using multivariate regression with L1 (lasso) regularisation, with genetic features and IGHV status as predictors. We generated a predictor profile for each drug‐stimulus combination, depicting coefficients that were selected in > 90% of bootstrapped model fits. In total, we found genetic modulators for 60/204 interactions (Fig 6A; Appendix Fig S18). Trisomy 12 and IGHV status modulated the largest number of interactions.

For example, we found the value of *β*_int_ for fludarabine and CpG ODN to be modulated by six genetic factors; most strongly by IGHV status, del(11q), and trisomy 12 (Fig 6B).

In IGHV‐M non‐trisomy 12 samples, fludarabine efficacy increased in the presence of TLR stimulation, whilst in the other subgroups TLR stimulation induced resistance to fludarabine (Fig 6C).

Our approach also identified interactions dependent on single treatment effects. IL4 induced complete resistance to ibrutinib regardless of higher ibrutinib efficacy in trisomy 12 and IGHV‐U samples. This highlights the breadth of IL4‐induced resistance to ibrutinib in CLL across genetic backgrounds (Fig 6D; Appendix Fig S19).

Overall, these findings illustrate how the presence of known genetic alterations determines how drugs and external stimuli interact with each other.

### The key resistance pathways IL4 and TLR show increased activity in CLL‐infiltrated lymph nodes

Microenvironmental signalling within lymph nodes has been implicated in treatment resistance (Yan *et al*, 2011; Hayden *et al*, 2012; Mittal *et al*, 2014; Herndon *et al*, 2017), though a clear understanding of the mechanisms involved remains missing. Since IL4 and TLR signalling was the most prominent modulators of drug response in our study, we assessed their activity within the lymph node niche. We stained paraffin‐embedded sections of 100 CLL‐infiltrated and 100 tumour‐free lymph nodes for pSTAT6, an essential downstream target of IL4 (Appendix Fig S20), and pIRAK4, a downstream target of TLR7/8/9.

**Figure 7:**
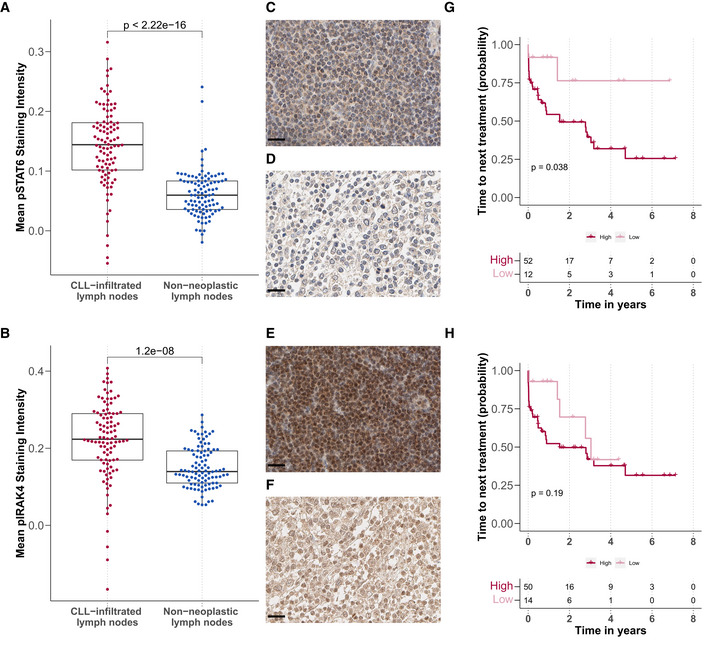
IL4 and TLR signalling is upregulated in CLL‐infiltrated lymph nodes A, BMean (A) pSTAT6 and (B) pIRAK4 staining intensity in CLL‐infiltrated (*n* = 100 for pSTAT and pIRAK4) and tumour‐free lymph node biopsies (*n* = 98 for pSTAT6 and *n* = 100 for pIRAK4) after background subtraction (*y*‐axis), *P*‐values from Student's *t*‐test. Each dot represents the mean of all cells in the tissue microarray cores per patient sample. The central bar, boxes and whiskers of the plot represent the median, first and third quartiles, and 1.5‐time IQR, respectively.C–FExample images of immunohistochemistry sections: (C + D) pSTAT6 levels in (C) CLL‐infiltrated and (D) tumour‐free samples. (E + F) pIRAK4 levels in (E) CLL‐infiltrated and (F) tumour‐free samples. Scale bar defines 20 μm in all images.G, HKaplan–Meier plots for time to next treatment stratified by levels (high/low) of pSTAT6 (G) and pIRAK4 (H). *P*‐values from univariate Cox proportional hazard models.

The CLL‐infiltrated lymph nodes showed higher levels of pSTAT6 (Fig 7A) and pIRAK4 (Fig 7B). Examples are shown in Fig 7C–F.

To investigate the influence of microenvironmental activity in lymph nodes on CLL disease progression, we correlated staining intensity with time to next treatment. High activity of pSTAT6 correlated with shorter time to next treatment (Fig 7G), and the same trend could be observed with higher pIRAK4 (Fig 7H). Higher IL4 activity within the lymph node appeared to relate to shorter time to next treatment and may provide further evidence to the hypothesis that microenvironmental activity promotes treatment resistance within the lymph node, eventually leading to relapse.

## Discussion

Our work maps the effects of microenvironmental stimuli in the presence of drugs and links these to underlying molecular properties across 192 primary CLL samples. We employ all combinations of 17 stimuli with 12 drugs as a reductionist model investigating individual effects of microenvironmental modulators. We account for the confounding effects of spontaneous apoptosis *ex vivo* and dissect the effect of individual microenvironmental stimuli on baseline viability and drug toxicity. The results may serve as building blocks for a more holistic understanding of the interactions of tumour genetics, microenvironment and drug response in complex *in vivo* situations.

We discover that CLL subgroups can be extracted from microenvironmental response phenotypes and that this classification is linked to distinct molecular profiles and clinical outcomes. A subgroup of IGHV‐M samples with distinct *ex vivo* responses to microenvironmental stimulation (C3) showed faster disease progression. This subgroup (C3) also demonstrated higher activity *in vivo* of pathways involved in response to microenvironmental signals, including NFκB, and was more susceptible to NFκB inhibition by BAY‐11‐7085. Overall, this highlights the important role of these signalling pathways in CLL pathophysiology. The question whether the distinct response *ex vivo* is caused by a higher intrinsic activity of these pathways *in vivo* remains open and would be an interesting topic of further research. Further, we link microenvironmental response phenotypes to the CLL Proliferative Drive (Lu *et al*, 2021), further characterising the biology of this newly proposed patient stratification method.

The sensitivity afforded by our approach enabled us to identify drug‐stimulus interactions, meaning instances where the effect of a drug on the tumour cells is modulated by a microenvironmental stimulus. Building on work on IL4‐ and IFN‐induced drug resistance (Aguilar‐Hernandez *et al*, 2016; Xia *et al*, 2020), our assay demonstrates the importance of both pathways in inducing resistance across a range of BCR inhibitors and chemotherapeutics. Systematic mapping of drug‐stimulus interactions, such as generated in this study, can be used to inform biology‐based combinatorial treatments across different entities.

We demonstrated the breadth of IL4‐induced resistance against kinase inhibitors and chemotherapeutics across a range of genetic backgrounds. IL4 induced complete resistance to BCR inhibitors across genetic subgroups, despite greater BCR inhibitor efficacy in IGHV‐U CLL. Further, we observed increased IL4 activity within CLL‐infiltrated lymph nodes compared with tumour‐free lymph nodes, which complements previous work demonstrating higher IL4 activity in the lymph node compared with peripheral blood (Aguilar‐Hernandez *et al*, 2016; Herishanu *et al*, 2011). Increased IL4 signalling activity was associated with faster disease progression. Our work highlights the significance of IL4 within the lymph node niche, especially since proliferation (Herndon *et al*, 2017) and resistance to BCR inhibition (Ahn *et al*, 2018) is linked to lymph nodes and our recent work suggests T follicular helper cells as a source of IL4 (Roider *et al*, 2020). Targeting pathways downstream of IL4, via JAK inhibition, has been reported to improve BCR response in non‐responding CLL patients (Spaner *et al*, 2019).

Underneath cell‐extrinsic factors that control the apoptotic and proliferative activity of tumour cells in response to drugs, there lies the network of cell‐intrinsic molecular components. We used multivariate modelling to capture the impact of molecular features on responses to stimuli and drugs. For instance, the response to TLR stimulation depended on IGHV status, trisomy 12, del11q, del13q and *ATM*, reflecting the multiple layers of biology involved. Most strikingly, we observed that TLR stimulation increased fludarabine toxicity in the subgroup of IGHV‐M non‐trisomy 12 patient samples, whilst it led to fludarabine resistance in all other backgrounds. This finding combined with our observation that TLR signalling is highly active in CLL‐infiltrated lymph nodes might help explain the heterogeneous effects of chemotherapy in CLL, in particular that fludarabine therapy can achieve lasting remission in IGHV‐M but not in IGHV‐U patients.

Beyond TLR, trisomy 12 modulated a broad range of stimulus–responses and drug‐stimulus interactions, comparable to the impact of IGHV status. Molecularly, we link the observed trisomy 12 phenotype to upregulated microenvironmental signalling and higher inferred activity of Spi‐B and PU.1, essential regulators of environmental sensing in B‐cells (Willis *et al*, 2017). The TF signature associated with trisomy 12 can be reversed by bromodomain inhibition and trisomy 12 samples exhibit increased sensitivity to bromodomain inhibition, indicating this as potential therapeutic strategy in trisomy 12 CLL. This is in line with previous epigenetic studies, suggesting BET inhibition as a viable therapeutic strategy in CLL (Ozer *et al*, 2018).

We present a data resource for the study of drug response in the context of cell‐intrinsic and cell‐extrinsic modulators. The data may inform targeted mechanistic investigations and direct efforts for combination therapies. The entire dataset, including the reproducible analysis, can be downloaded *in toto* from the online repository (github.com/Huber‐group‐EMBL/CLLCytokineScreen2021), or explored interactively (dietrichlab.de/CLL_Microenvironment/).

## Materials and Methods

### Reagents and Tools table

**Table:** 

Reagent/Resource	Reference or Source	Identifier or Catalog Number
**Experimental Models**		
Information on patient samples is provided in Table EV1		
**Antibodies**		
pIRAK4	Abcam; Lot: GR3228799‐1; Clone: polyklonal; Host: rabbit; Dilution: 1:50	ab216513
pSTAT6	Abcam; Lot: GR45152‐32; Clone: polyklonal; Host: rabbit; Dilution: 1:50	ab28829
**Chemicals, enzymes and other reagents**		
***Drugs***		
Ibrutinib	Selleck Chemicals; Concentration 1: 500 nM; Concentration 2: 50 nM	S2680
Idelalisib	Selleck Chemicals; Concentration 1: 500 nM; Concentration 2: 50 nM	S2226
Fludarabine	Selleck Chemicals; Concentration 1: 2,000 nM; Concentration 2: 200 nM	S1491
Nutlin‐3a	Selleck Chemicals; Concentration 1: 10,000 nM; Concentration 2: 1,000 nM	S8059
Selumetinib	Selleck Chemicals; Concentration 1: 1,000 nM; Concentration 2: 100 nM	S1008
BAY‐11‐7085	Selleck Chemicals; Concentration 1: 2,000 nM; Concentration 2: 200 nM	S7352
Everolimus	Selleck Chemicals; Concentration 1: 500 nM; Concentration 2: 50 nM	S1120
PRT062607	Selleck Chemicals; Concentration 1: 500 nM; Concentration 2: 50 nM	S8032
Pyridone‐6	MedChemExpress; Concentration 1: 500 nM; Concentration 2: 50 nM	457021‐03‐7
Ralimetinib	Selleck Chemicals; Concentration 1: 1,500 nM; Concentration 2: 150 nM	S1494
Luminespib	Selleck Chemicals; Concentration 1: 200 nM; Concentration 2: 20 nM	S1069
I‐BET 762	Selleck Chemicals; Concentration 1: 1,000 nM; Concentration 2: 100 nM	S7189
***Microenvironmental stimuli***		
IL4 human recombinant animal component free	Sigma‐Aldrich; Concentration: 10 ng/ml; Lot: 0712AFC14	SRP3093
IL10 human Animal component free	Sigma‐Aldrich; Concentration: 10 ng/ml; Lot: 1012AFC21	SRP3312
IL2 human recombinant animal component free	Sigma‐Aldrich; Concentration: 10 ng/ml; Lot: 0416AFC12	SRP3085
R‐848	Enzo Life Siences; Concentration: 1,000 ng/ml; Lot: 10211615	ALX‐420‐038‐M025
Human IL‐21	Peprotech; Concentration: 10 ng/ml; Lot: 414226	200‐21
Human BAFF	Peprotech; Concentration: 250 ng/ml; Lot: 0706CY194	310‐13
Human IL‐1 beta	Peprotech; Concentration: 10 ng/ml; Lot: 0606B95	200‐01
Human sCD40 Ligand	Peprotech; Concentration: 1,000 ng/ml; Lot: 1214145	310‐02
Goat F(AB')2 Fragment to human IgM	MP Biomedicals; Concentration: 20,000 ng/ml; Lot: 7227	55055
Human TGFβ1	Peprotech; Concentration: 10 ng/ml; Lot: 1117209	100‐21
Human IL15	Peprotech; Concentration: 10 ng/ml; Lot: 91624	200‐15
Human IL6	Peprotech; Concentration: 10 ng/ml; Lot: 031316‐2	200‐06
ODN 2006 (ODN 7909)	Invivogen; Concentration: 1,000 ng/ml; Lot: 3901‐09T	tlrl‐2006‐1
Human SDF1 alpha (CXCL12)	Peprotech; Concentration: 200 ng/ml; Lot: 101492	300‐28A
Human Interferon gamma	Peprotech; Concentration: 5 ng/ml; Lot: 121527	300‐02
HS‐5 conditioned medium	this study; Concentration: 20%	
***Other reagents***		
RPMI	Gibco®	21875‐034
Pen/Strep	Gibco®	15140‐122
L‐Glutamine	Gibco®	25030‐024
FBS	PAN BIOTECH™	P30‐3302
Human Serum	PAN BIOTECH™	P40‐2701
**Software**		
R		4.1.0
R Studio		2022.02.0 Build 443
Qupath		0.1.2
HOCOMOCO		10
diffTF		1.8
broom		0.8.0
ChIPseeker		1.30.3
clusterProfiler		4.2.2
ConsensusClusterPlus		1.56.0
cowplot		1.1.1
DESeq2		1.34.0
DESeq2		
dplyr		1.0.7
genomation		1.24.0
ggbeeswarm		0.6.0
ggfortify		0.4.14
ggplot2		3.3.5
ggpubr		0.4.0
ggrepel		0.9.1
glmnet		4.1‐2
gridExtra		2.3
gtable		0.3.0
Hmisc		4.6‐0
magick		2.7.3
magrittr		2.0.2
maxstat		0.7‐25
msigdbr		7.5.1
org.Hs.eg.db		3.13.0
patchwork		1.1.1
pheatmap		1.0.12
plyr		1.8.6
png		0.1‐7
RColorBrewer		1.1‐3
scales		1.2.0
survival		3.2‐12
survminer		0.4.9
tidyr		1.1.3
tidyverse		1.3.1
TxDb.Hsapiens.UCSC.hg19.knownGene		3.2.2
**Other**		
40 μm Cell Strainer	Falcon®	352340
CellTiter‐Glo	Promega®	G7573
384‐well deepwell plates	Greiner Bio‐One	781270
Aluminium Plate Seals	Greiner Bio‐One	676090
384‐well culture plates	Greiner Bio‐One	781904
6–well plates	Greiner Bio‐One	657160

### Methods and protocols

#### Sample preparation and drug‐stimulation profiling

Informed consent was obtained from all patients before isolation and storage of CLL cells in line with the Declaration of Helsinki and the Department of Health and Human Services Belmont Report. The study was approved by the ethics committee of the Medical Faculty Heidelberg, Germany. Mononuclear cells were isolated from peripheral blood of CLL patients by a Ficoll gradient and cryopreserved as previously described (Dietrich *et al*, 2018). Cells were incubated for 48 h with or without drugs and stimuli. We selected drugs that produced diverse patterns of responses across patient samples and that covered targets important to the biology of CLL. For each drug, we selected two concentrations, one inducing subtle, sublethal effects, the other killing a large fraction of cells. This was done to enable observation of both synergistic and protective effects with additional exposition to stimuli. All drugs were dissolved in dimethyl sulfoxide (DMSO) and stored at −20°C. The stimuli were chosen based on literature review of small scale *in vitro* studies in CLL. Concentrations were based on previously reported studies and on our own experiments. HS‐5‐conditioned medium was produced by incubating HS‐5 stromal cell line to > 80% confluency and cell removal by centrifugation. The viability was measured by a luminescent ATP readout (CellTiter‐Glo, Promega®). The detailed methodology is described below.

- Preparation of drug‐stimulus combinations in 384‐well plates
  - All drugs were diluted in DMSO to 333‐fold target concentration in the culture (see Reagents and Tools table). These dilutions were performed manually and pipetted into a 384‐well deep‐well plate (Greiner Bio‐One cat.no. 781270).
  - 1.5 μl of drug solution was pipetted per well into 384‐well plates (Greiner Bio‐One cat.no. 781904). These plates were sealed with aluminium foil (Greiner Bio‐One cat.no. 676090) and stored at −20°C.
  - For each day of screening, two 384‐well drug plates were thawed.
  - Stimuli were diluted and stored according to the protocols defined by the manufacturers (Reagents and Tools table). On each screening day, all stimuli were thawed and diluted in RPMI + 1% Pen/Strep + 1% Glutamine.
  - 98.5 μl of stimuli solution was pipetted into the 384‐well drug plates containing 1.5 μl of drug solution per well.
  - Using a high‐throughput liquid handling device (Integra Viaflo), 5 μl of drug‐stimulus mixture was added into 384‐well culture plates. Two culture plates were used per patient sample.
- Patient samples
  - Mononuclear cells were isolated from peripheral blood and cryopreserved as previously described (Dietrich *et al*, 2018).
  - Samples were removed from liquid nitrogen and transported on dry ice to the cell culture facility. The samples were incubated in a water bath at 37°C until they were thawed. The samples were then transferred to 50 ml centrifugation tubes with 10 ml of RPMI + 10% FBS + 1% Pen/Strep + 1% Glutamine.
  - Samples were centrifuged at 400 *g* for 5 min.
  - The supernatant was removed quickly, taking care not to disturb the cell pellet.
  - 10 ml of RPMI + 10% human serum + 1% Pen/Strep + 1% Glutamine were added, and the cell pellet was resuspended.
  - Cells were incubated for 3 h on a roll mixer. This step allowed for reconstitution of cellular metabolism after freezing and thawing. Additionally, dead cells clumped together and could be filtered out, ensuring high viability at the beginning of each experiment.
  - Cell suspensions were filtered through 40 μm filters.
  - 10 μl of cell suspension was stained with trypan blue, and cells were counted in a Neubauer chamber to calculate the dilution for the next step.
  - Cells were diluted in RPMI + 10% human serum + 1% Pen/Strep + 1% Glutamine to 1 × 10^6^ cells/ml.
- Viability screening
  - 20 μl of cell suspension was added onto previously prepared culture plates containing drugs and stimuli.
  - Patient samples were incubated with drugs and stimuli in culture plates at 37°C and 5% CO_2_ for 48 h.
  - CellTiter‐Glo was used to measure cell viability. Prior to screening, CellTiter‐Glo was dissolved and stored according to the manufacturer's protocol and was thawed and equilibrated to room temperature on screening days.
  - 7 μl of CellTiter‐Glo was added to each well and left to incubate for 20 min at room temperature in a dark environment.
  - Luminescence was measured on a Perkin Elmer EnVision using a measurement time of 100 ms.

#### ATAC sequencing

CLL cells were isolated from four patients as described above and MACS sorted for CD19 positive cells (Miltenyi autoMACS). 5 ml of cell suspension was cultured in 6–well plates and incubated at 37°C and 5% CO_2_ for 6 h in DMSO or IBET 762. The final cell concentration was 2 × 10^6^ cells/ml. Cell viability and purity was assessed using FACS. All samples had a viability over 90% and over 95% of CD19^+^/CD5^+^/CD3^−^ cells.

#### ATAC sequencing library generation

ATAC sequencing libraries were generated as described previously (Buenrostro *et al*, 2015). Cell preparation and transposition was performed according to the protocol, starting with 5 × 10^4^ cells per sample. Purified DNA was stored at −20°C until library preparation was performed. To generate multiplexed libraries, the transposed DNA was initially amplified for 5x PCR cycles using 2.5 μl each of 25 μM PCR Primer 1 and 2.5 μl of 25 μM Barcoded PCR Primer 2 (Illumina), 25 μl of NEBNext High‐Fidelity 2x PCR Master Mix (New England Biolabs) in a total volume of 50 μl. A 5 μl of the amplified DNA was used to determine the required number of additional PCR cycles for each library using qPCR by plotting the line of Rn versus cycle and extrapolating to one‐third of the maximum fluorescent intensity. The largest number of additional cycles thus determined was 13. Finally, amplification was performed on the remaining 45 μl of the PCR reaction. The amplified fragments were purified with two rounds of SPRI bead clean‐up (1.4x). The size distribution of the libraries was assessed on a Bioanalyzer with a DNA High Sensitivity kit (Agilent Technologies), and concentration was measured with Qubit® DNA High Sensitivity kit in Qubit® 2.0 Fluorometer (Life Technologies). Sequencing was performed on NextSeq 500 (Illumina) using 75 bp paired‐end sequencing, generating approx. A 450 million paired reads per run, with an average of 55 million reads per sample.

#### Immunohistochemistry

Lymph node biopsies of CLL‐infiltrated and tumour‐free samples were formalin‐fixed, paraffin‐embedded, arranged in Tissue Microarrays and stained for pSTAT6 (ab28829, Abcam) and pIRAK4 (ab216513, Abcam). The slides were analysed using Qupath (Bankhead *et al*, 2017) and the recommended protocol.

#### Data processing and statistical analysis

To quantify the responses to drugs and stimuli, we used a measure of viability relative to the control, namely the natural logarithm of the ratio between CellTiter‐Glo luminescence readout of the respective treatment and the median of luminescence readouts of the DMSO control wells on the same plate, excluding controls on the outer plate edges. Downstream data analysis was performed using R version 4 and using packages including DESeq2 (Love *et al*, 2014), survival (Therneau & Grambsch, 2000), Glmnet (Friedman *et al*, 2010), ConsensusClusterPlus (Wilkerson & Hayes, 2010), clusterProfiler (Yu *et al*, 2012), ChIPseeker (Yu *et al*, 2015), and genomation (Akalin *et al*, 2015) to perform univariate association tests, multivariate regression with and without lasso penalisation, Cox regression, generalised linear modelling and clustering. The complete analysis is described along with computer‐executable transcripts at github.com/Huber‐group‐EMBL/CLLCytokineScreen2021.

For Fig 2A, the data are shown on a robust *z*‐score scale, that is, the logarithm of the relative viability measurements was scaled by the median absolute deviation within each row. Limits were applied to scaling factor for optimal visualisation. To generate the column‐wise clusters, we performed repeated hierarchical clustering with the Euclidean metric over 10,000 repetitions with randomly selected sample subsets of 80% using ConsensusClusterPlus (Wilkerson & Hayes, 2010).

For Fig 2B, lymphocyte doubling time was calculated as described in (Lu *et al*, 2021). For Fig 2C, TTT represents the period between the date of sample collection and the data of treatment initiation. To calculate significance, univariate Cox proportional hazards regression models were fitted using the coxph function, using C1 as reference for the comparison C1 versus C2 and C4 as reference for C3 versus C4. To determine whether the prognostic value of cluster assignment between C3 and C4 was independent of other prognostic markers, a multivariate Cox proportional hazards regression model was fit, with the design formula ~ Cluster + IGHV.status + trisomy 12 + TP53 + Methylation cluster + POT1, with Cluster 3 as reference. In Fig 2D, multinomial regression with lasso penalisation with a matrix of genetic features (*n* = 39) and IGHV status (encoded as *M* = 1 and *U* = 0) was used to identify multivariate predictors of cluster assignment. The resulting coefficients are the mean of 50 bootstrapped repeats, where coefficients were filtered if they were selected in < 60% of cases or were < 0.35. Error bars represent the mean ± standard deviation from bootstrapped repeats. Genetic features with > 20% missing values were excluded, and only patients with complete annotation were included in the model (*n* = 129). For Fig 2E and Appendix Figs S7 and S8, to quantify differential gene expression between C1 (*n =* 17) and C2 (*n =* 11), and C3 (*n =* 9) and C4 (*n =* 12), genes encoding components of the BCR were first filtered, including genes at the heavy, light and kappa immunoglobulin loci. Differential expression was quantified using DESeq2 (Love *et al*, 2014) with design formula ~ IGHV.status + Cluster. For the GSEA, genes were ranked according to the resulting Wald statistics and GSEA was performed using the fgsea algorithm and using Hallmark gene sets.

Significance testing for genetic determinants of microenvironmental response shown in Fig 3A was performed for somatic mutations and copy number aberrations present in ≥ 3 patients, and IGHV status (*n* = 54). For multivariate modelling with lasso penalisation shown in Fig 3B, to generate the feature matrix, genetic features with < 20% missing values were considered, *KRAS*, *BRAF* and *NRAS* mutations were summarised as *RAS/RAF* alterations and only patient samples with complete genetic annotation were tested. In total, 39 genetic features as well as IGHV status (encoded as *M =* 1 and *U =* 0), and Methylation Cluster (encoded as LP = 0, IP = 0.5, HP = 1) and 129 patients were included in this analysis. The model was fit for 30 bootstrapped repeats, and the resulting coefficients are the mean of those coefficients that were selected in > 75% model fits.

For the ATAC sequencing and inferred TF activity analyses described in Fig 4, we first generated bam and peak files from fastqs and subsequently ran the diffTF software (Berest *et al*, 2019) to infer TF activity as follows.

In Fig 4D, we selected raw sequencing files from (Rendeiro *et al*, 2016) to include one sample per patient passing quality checks (*n* = 52) and obtained bam files mapped to the hg19 genome and adjusted for CG bias as previously outlined (Berest *et al*, 2019). In Appendix Fig S15, we selected raw sequencing files from (Beekman *et al*, 2018) to include 100 samples and obtained bam files mapped to the hg19 genome as previously outlined (Berest *et al*, 2019) with the exception that we did not adjust for GC‐bias.

In both datasets, as trisomy 12 status was not already annotated, we called trisomy 12 in samples that contained > 1.4 times more reads per peak on average in chromosome 12, compared with peaks on other chromosomes, based on the consensus set of peaks that we used to run diffTF. We used diffTF in permutation mode (Berest *et al*, 2019) to infer the differential TF activity between trisomy 12 and non‐trisomy 12 samples using TF motifs from HOCOMOCO v10 (Kulakovskiy *et al*, 2016). For the (Rendeiro *et al*, 2016) data, we used minOverlap = 2 and design formula: “~ sample_processing_batch + sex + IGHV status + trisomy 12” and for the (Beekman *et al*, 2018) data, we used minOverlap = 5 and design formula “~ sex + IGHV status + trisomy 12.”

In Appendix Fig S14, raw ATACseq data generated from our CLL samples were processed as described by (Berest *et al*, 2019), with the exception that we did not use CG bias correction. We used analytical mode of diffTF with TF motifs from HOCOMOCO v10 database (Kulakovskiy *et al*, 2016) using the following parameters: minOverlap = 1; design formula = “~ Patient + trisomy 12 status.” In both datasets, the analysis was repeated by first removing all peaks located on chromosome 12 from the input peak file provided to diffTF, to test the dependency of the results on the third copy of chromosome 12.

For Fig 4F, raw ATACseq data generated from IBET‐762 and DMSO‐treated CLL samples were processed as described in Berest *et al*, 2019, mapped to hg38. We then ran diffTF in analytical mode with TF motifs from HOCOMOCO v11 database (Kulakovskiy *et al*, 2016) using the following parameters: minOverlap = 2; design formula = “~ patient sample + treatment (IBET‐762 or DMSO control).” The inferred TF activity values of the separate diffTF analyses (trisomy 12 vs. non‐trisomy 12 and IBET‐762 vs. DMSO‐treated cells) are not directly comparable. Significantly different TFs from the trisomy 12 vs. non‐trisomy 12 analysis are shown.

For the analysis of Spi‐B and PU.1 gene targets in Appendix Table S5, we began with the summary files from running diffTF on the ATACseq data from (Rendeiro *et al*, 2016), as described above. The summary files contained Log2 fold changes and associated *P*‐values for all motif locations of Spi‐B and PU.1 (motifs were defined using the HOCOMOCO database). Spi‐B and PU.1 motif locations were selected where the absolute Log2 fold change between trisomy 12 and non‐trisomy 12 samples > 1 and the associated adjusted *P*‐value < 0.1. These motif locations are plotted in Appendix Fig S17. We next defined Spi‐B and PU.1 targets as the closest gene to each of these motif locations. In Appendix Table S6, Spi‐B and PU.1 gene targets were defined as the closest gene to each significant ChIP peak (*q* value < 0.05) and within ±1 kb of transcription start site. For both analyses, over‐representation tests of immune pathways taken from the KEGG and Reactome databases, plus control pathways, were run using the clusterProfiler package (Yu *et al*, 2012). The method corresponds to a one‐sided version of Fisher's exact test.

To generate predictor profiles for Fig 6A, the linear model in Equation (1) was first fitted separately for each patient sample to calculate drug‐stimulus interaction coefficients (*β*_int_) for each sample. Associations between the size of these sample‐specific *β*_int_ and genetic features were next identified using multivariate regression with lasso regularisation with genetic alterations (*n* = 39) and IGHV status (*U* = 0, *M* = 1) as feature matrix and selecting coefficients that were chosen in > 90% of bootstrapped model fits.

For Fig 7G and H, pSTAT6 and pIRAK4 groups were defined using maximally selected rank statistics based on staining intensities. The same 64 CLL lymph node samples for which survival data were available were used for both Kaplan–Meier plots.

#### Analysed data from previous studies

We obtained CLL ATACseq data (Rendeiro *et al*, 2016; Beekman *et al*, 2018) from the EGA (EGAD00001002110, EGAD00001004046) and ChIPseq data for Spi‐B and PU.1 binding (Care *et al*, 2014) from the NCBI GEO database (Edgar *et al*, 2002) (GSE56857, GSM1370276, GSM1370275).

## Author contributions

**Peter‐Martin Bruch:** Conceptualization; data curation; formal analysis; validation; investigation; visualization; methodology; writing – original draft; project administration; writing – review and editing. **Holly AR Giles:** Conceptualization; formal analysis; investigation; visualization; methodology; writing – original draft; writing – review and editing. **Carolin Kolb:** Data curation; writing – review and editing. **Sophie A Herbst:** Data curation; validation; investigation; visualization; writing – review and editing. **Tina Becirovic:** Data curation; writing – review and editing. **Tobias Roider:** Data curation; formal analysis; visualization; writing – review and editing. **Junyan Lu:** Software; formal analysis; validation; methodology; writing – review and editing. **Sebastian Scheinost:** Data curation; writing – review and editing. **Lena Wagner:** Data curation; writing – review and editing. **Jennifer Huellein:** Methodology; writing – review and editing. **Ivan Berest:** Formal analysis; investigation; methodology; writing – review and editing. **Mark Kriegsmann:** Data curation; investigation; writing – review and editing. **Katharina Kriegsmann:** Data curation; writing – review and editing. **Christiane Zgorzelski:** Data curation; investigation; writing – review and editing. **Peter Dreger:** Writing – review and editing. **Judith B Zaugg:** Formal analysis; supervision; methodology; writing – review and editing. **Carsten Müller‐Tidow:** Resources; supervision; writing – review and editing. **Thorsten Zenz:** Conceptualization; resources; writing – review and editing. **Wolfgang Huber:** Conceptualization; resources; data curation; supervision; funding acquisition; methodology; writing – original draft; writing – review and editing. **Sascha Dietrich:** Conceptualization; resources; data curation; supervision; funding acquisition; methodology; writing – original draft; project administration; writing – review and editing.

In addition to the CRediT author contributions listed above, the contributions in detail are:

S.D. and P.M.B. designed the experiments. S.D., W.H., H.A.R.G. and P.M.B. conceptualised the data analysis. W.H. and S.D. supervised the study. W.H., T.Z. and S.D. conceptualised the multi‐omics approach to analyse CLL drug response. P.M.B. performed the drug‐stimulus profiling experiments with support from C.K., S.S. and L.W. T.Z., C.M.T. and P.D. provided guidance on experimental protocols. H.A.R.G., P.M.B, J.H., J.L. and S.D. performed data processing and curation. H.A.R.G. and P.M.B. analysed and interpreted the data, with support from W.H., S.D., J.L, S.H. and T.R. and produced the figures with input from W.H. and S.D. H.A.R.G. performed statistical inference and regression modelling, and P.M.B. performed exploratory analysis and integration with clinical data. T.B. and S.H. performed the shRNA knockdown experiments, J.B.Z. and I.B. provided the ATACseq dataset used in Fig 4 and ran the alignments for the ATACseq data in Fig 4 and Appendix Fig S15 and quantified differential transcription factor activity, M.K., K.K. and C.Z. generated the immunohistochemistry data, and P.M.B. analysed these data. H.A.R.G. designed and wrote the Shiny app and the software documentation. P.M.B. and H.A.R.G. wrote the manuscript, with input from S.D. and W.H. All authors reviewed and edited the manuscript.

## Disclosure and competing interests statement

The authors declare that they have no conflict of interest.

## Supporting information




Appendix S1

Table EV1

## Data Availability

The datasets and computer code produced in this study are available in the following databases:
Viability data including patient annotation and immunohistochemistry results: BioStudies (Sarkans *et al*, 2018) accession number S‐BSST824 (https://www.ebi.ac.uk/biostudies/studies/S‐BSST824). ATAC sequencing data: European Genome‐Phenome Archive (EGA) accession number EGAD00001008723 (https://ega‐archive.org/datasets/EGAD00001008723). Reproducible data analysis scripts: GitHub (github.com/Huber‐group‐EMBL/CLLCytokineScreen2021). An online application is provided at dietrichlab.de/CLL_Microenvironment/ for rapid data exploration of the viability data.
