# Supplementary material for: Drug‐microenvironment perturbations reveal resistance mechanisms and prognostic subgroups in CLL
Source: Mol Syst Biol. 2022 Aug 12;18(8):e10855. doi: 10.15252/msb.202110855 (PMC9372727; doi:10.15252/msb.202110855)
Supplement: Supplementary file 1 — Appendix S1 [file MSB-18-e10855-s003.pdf]

Drug-microenvironment perturbations reveal resistance mechanisms and prognostic subgroups in CLL

Appendix

Peter-Martin Bruch*, Holly A. R. Giles*, Carolin Kolb , Sophie A. Herbst, Tina Becirovic, Tobias Roider, Junyan Lu, Sebastian Scheinost, Lena Wagner, Jennifer Huellein, Ivan Berest, Mark Kriegsmann, Katharina Kriegsmann, Christiane Zgorzelski, Peter Dreger, Judith B. Zaugg, Carsten Müller-Tidow, Thorsten Zenz, Wolfgang Huber*, Sascha Dietrich* * These authors contributed equally to this work.

14th April 2022

Contents

Appendix Tables **3**

Appendix Table S1. List of genetic features tested for their association with stimulus response in the univariate model of Figure 3A. 4

Appendix Table S2. Survival analysis of microenvironmental response clusters comparing C3 to C1, C2 and C4. 4

Appendix Table S3. Survival analysis of microenvironmental response clusters comparing C3 to C1 and C2 combined. 4

Appendix Table S4. Multivariate survival analysis of response clusters. 4

Appendix Table S5. Over-representation tests of immune signalling pathways amongst Spi-B and PU.1 gene targets, based on primary CLL ATACseq data. 5

Appendix Table S6. Over-representation tests of immune signalling pathways amongst Spi-B and PU.1 gene targets, based on DLBCL ChIPseq data from Care et al. 2014[1]. 6

Appendix Figures **7**

Appendix Figure S1. Genetic profiles of screened patient samples. 7

Appendix Figure S2. Response to stimuli stratified by IGHV status. 8

Appendix Figure S3. Stimulus response stratified by clusters. 9

Appendix Figure S4. Time to first treatment and overall survival by clusters. 10

Appendix Figure S5. The relationship between CLL Proliferative Drive (as defined by Lu et al. (2021)[2]) and microenvironmental response. 11

Appendix Figure S6. DNA Methylation profile of Clusters defined by response to microenvironmental stimuli 13

Appendix Figure S7. RNA-Sequencing of matched samples indicates differential gene expression between Cluster 3 and Cluster 4. 15

Appendix Figure S8. RNA-Sequencing of matched samples indicates differential gene expression between Cluster 1 and 2. 16

Appendix Figure S9. Drug response between clusters. 17

Appendix Figure S10. Drug response by clusters. 18

Appendix Figure S11. Effect of BAY-11-7085 treatment on BAFF response 19

Appendix Figure S12. Correlation of stimulus response and receptor expression. 20

Appendix Figure S13. Predictor profiles to represent genetic feature-stimulus associations. . . . 21

Appendix Figure S14. ATACseq comparing trisomy 12 and non-trisomy 12 CLL samples. 22
Appendix Figure S15. ATACseq comparing trisomy 12 and non-trisomy 12 CLL samples. 23
Appendix Figure S16. ATACseq comparing trisomy 12 and non-trisomy 12 CLL samples, independent of third copy of chromosome 12 25
Appendix Figure S17. Chromosomal locations of Spi-B and PU.1 motifs that show differential accessibility between trisomy 12 and non-trisomy 12 CLL. 26
Appendix Figure S18. Genetic predictors of drug-stimulus interactions. 27
Appendix Figure S19. Genetic predictors of the interaction between ibrutinib and IL4. 29
Appendix Figure S20. STAT6 dependency of IL4 signaling. 30

References **31**

Appendix Tables

Genetic feature	Wildtype/IGHV unmutated	Mutated/ IGHV Mutated	Not available
ATM	164	16	12
BIRC3	177	3	12
CARD11	135	3	54
Complex_Karyotype	115	31	46
CREBBP	135	3	54
CSMD3	175	5	12
del11p	143	3	46
del11q	148	28	16
del12q	171	3	18
del13q	66	108	18
del14q	145	5	42
del15q	141	5	46
del17p	154	20	18
del17q	143	3	46
del18p	141	5	46
del1q	141	5	46
del21q	143	3	46
del3p	143	3	46
del3q	143	3	46
del4p	143	3	46
del4q	143	3	46
del6q	166	7	19
del7q	143	3	46
del8p	135	11	46
del9p	140	6	46
del9q	142	4	46
EGR2	176	4	12
FAT2	175	5	12
FAT4	176	4	12
FBXW7	178	3	11
FLRT2	135	3	54
gain17q	143	3	46
gain18q	144	4	44
gain19p	142	4	46
gain19q	142	4	46
gain1q	143	3	46
gain2p	137	9	46
gain8q	159	13	20
IGHV.status	83	99	10
IKZF3	176	4	12
MED12	170	10	12
MYD88	184	3	5
NFKBIE	175	5	12
NOTCH1	137	24	31
POT1	174	6	12
Ras_Raf	170	15	7
RYR2	177	3	12
SF3B1	161	26	5
SPEN	177	3	12
TP53	157	30	5
trisomy12	149	25	18
U1	137	5	50
XPO1	175	5	12
ZMYM3	134	4	54

Appendix Table S1. List of genetic features tested for their association with stimulus response in the univariate model of Figure 3A.

Only genetic features with at least three positive cases were considered.

Factor	Hazard Ratio	p-value	CI Low	CI High
Cluster 3 vs Cluster 1	1.27	0.37	0.75	2.16
Cluster 3 vs Cluster 2	2.06	0.03	1.07	3.93
Cluster 3 vs Cluster 4	0.35	0.01	0.17	0.73

Appendix Table S2. Survival analysis of microenvironmental response clusters comparing C3 to C1, C2 and C4.

Cox proportional hazards model of stimulus response clusters using time to next treatment and with C3 as reference.

Factor	Hazard Ratio	p-value	CI Low	CI High
Cluster 3 vs combined Clusters 1 and 2	1.42	0.17	0.86	2.36

Appendix Table S3. Survival analysis of microenvironmental response clusters comparing C3 to C1 and C2 combined.

Cox proportional hazards model of stimuli response clusters using TTT.

Factor	Hazard Ratio	p-value	CI Low	CI High
Cluster 3 vs Cluster 1	0.95	0.87	0.52	1.74
Cluster 3 vs Cluster 2	1.64	0.23	0.74	3.63
Cluster 3 vs Cluster 4	0.34	0.03	0.13	0.88
IGHV.status	1.95	0.32	0.53	7.20
trisomy 12	1.04	0.93	0.48	2.23
TP53	3.88	<0.0001	2.25	6.68
Methylation_ClusterIP	1.57	0.3	0.67	3.70
Methylation_ClusterLP	1.00	1	0.25	4.04
POT1	2.65	0.07	0.93	7.54

Appendix Table S4. Multivariate survival analysis of response clusters.

Multivariate Cox proportional hazards model of stimulus response clusters and genetic subgroups of disease progression using time to next treatment with C3 as reference.

Pathway	Geneset Database	Geneset Size	Spi-B targets (/333)	Spi-B p-value	Spi-B adj. p-value	PU.1 targets (/423)	PU.1 p-value	PU.1 adj. p-value
JAK STAT signaling pathway	KEGG	155	8	**0.005**	0.098	7	0.021	0.207
RLR Signaling Pathway	KEGG	71	3	0.133	0.717	1	0.742	0.939
Signaling by TGFbeta Receptor Complex	Reactome	73	3	0.141	0.717	3	0.151	0.597
Interleukin 10 Signaling	Reactome	46	2	0.2	0.717	1	0.581	0.894
Interleukin 15 Signaling	Reactome	14	1	0.225	0.717	0	1	1.000
Interleukin 1 Signaling	Reactome	102	3	0.277	0.717	1	0.859	0.955
TCR Signaling Pathway	KEGG	108	3	0.307	0.717	7	**0.003**	0.058
Signaling by Interleukins	Reactome	463	10	0.311	0.717	8	0.655	0.936
Interleukin 4 and Interleukin 13 Signaling	Reactome	111	3	0.322	0.717	3	0.339	0.666
BCR Signaling Pathway	KEGG	75	2	0.394	0.723	3	0.159	0.597
Chemokine Signaling Pathway	KEGG	189	4	0.447	0.723	5	0.269	0.597
DNA Replication	KEGG	36	1	0.483	0.723	0	1	1.000
Cytokine Cytokine Receptor Interaction	KEGG	265	5	0.53	0.723	6	0.366	0.666
TLR Signaling Pathway	KEGG	102	2	0.557	0.723	1	0.859	0.955
Regulation of TP53 Pathway	Reactome	160	3	0.56	0.723	6	0.07	0.464
Notch Signaling Pathway	KEGG	47	1	0.579	0.723	2	0.216	0.597
Cell Cycle	Reactome	693	12	0.631	0.743	7	0.99	0.990
Interferon Alpha Beta Signaling	Reactome	73	1	0.741	0.824	1	0.751	0.939
Interferon Gamma Signaling	Reactome	93	1	0.823	0.866	3	0.246	0.597
Oxidative Phosphorylation	KEGG	132	1	0.917	0.917	1	0.923	0.971

Appendix Table S5. Over-representation tests of immune signalling pathways amongst Spi-B and PU.1 gene targets, based on primary CLL ATACseq data.

Gene sets were selected from KEGG and Reactome databases. Control pathways i.e not related to immune signalling (Oxidative Phosphorylation, TP53 signalling and DNA Replication) were also included in the analysis and are not significant. 25 genes sets were tested, results for all genesets containing at least one target gene are shown.

Pathway	Geneset Database	Geneset Size	Spi-B targets (/3253)	SPIB p-value	SPIB adj. p-value	PU.1 targets (/250)	PU.1 p-value	PU.1 adj. p-value
BCR Signaling Pathway	KEGG	75	36	**0**	**0**	4	0.056	0.337
TLR Signaling Pathway	KEGG	102	40	**0**	**0.001**	5	0.046	0.337
Signaling by TGFbeta Receptor Complex	Reactome	73	26	**0.007**	0.061	0	1.000	1.000
TCR Signaling Pathway	KEGG	108	35	0.011	0.069	3	0.356	0.800
Interleukin 1 Signaling	Reactome	102	32	0.024	0.111	3	0.323	0.800
TNF Signaling	Reactome	44	16	0.027	0.111	0	1.000	1.000
RLR Signaling Pathway	KEGG	71	22	0.064	0.208	1	0.761	0.975
Signaling by Interleukins	Reactome	463	117	0.074	0.208	12	0.173	0.518
Interleukin 6 Signaling	Reactome	11	5	0.081	0.208	0	1.000	1.000
Interferon Alpha Beta Signaling	Reactome	73	22	0.083	0.208	2	0.423	0.845
Chemokine Signaling Pathway	KEGG	189	50	0.115	0.262	6	0.158	0.518
NLR Signaling Pathway	KEGG	62	18	0.146	0.305	1	0.713	0.975
Interferon Gamma Signaling	Reactome	93	25	0.194	0.373	2	0.552	0.975
DNA Replication	KEGG	36	9	0.434	0.776	2	0.155	0.518
Cell Cycle	Reactome	693	158	0.494	0.823	19	0.047	0.337
Regulation of TP53 Pathway	Reactome	160	36	0.56	0.875	2	0.835	0.975
Notch Signaling Pathway	KEGG	47	10	0.651	0.957	0	1.000	1.000
Interleukin 10 Signaling	Reactome	46	9	0.751	1	1	0.602	0.975
Interleukin 2 Signaling	Reactome	12	2	0.795	1	0	1.000	1.000
Other Interleukin Signaling	Reactome	24	4	0.83	1	0	1.000	1.000
JAK STAT signaling pathway	KEGG	155	30	0.874	1	1	0.959	0.975
Interleukin 4 and Interleukin 13 Signaling	Reactome	111	20	0.912	1	1	0.896	0.975
Interleukin 15 Signaling	Reactome	14	1	0.973	1	0	1.000	1.000
Oxidative Phosphorylation	KEGG	132	19	0.995	1	1	0.933	0.975
Cytokine Cytokine Receptor Interaction	KEGG	265	27	1	1	2	0.975	0.975

Appendix Table S6. Over-representation tests of immune signalling pathways amongst Spi-B and PU.1 gene targets, based on DLBCL ChIPseq data from Care et al. 2014[1].

Gene sets were selected from KEGG and Reactome databases. Control pathways i.e not related to immune signalling (Oxidative Phosphorylation, TP53 signalling and DNA Replication) were also included in the analysis and are not significant. 25 genesets were tested, all are shown.

Appendix Figures

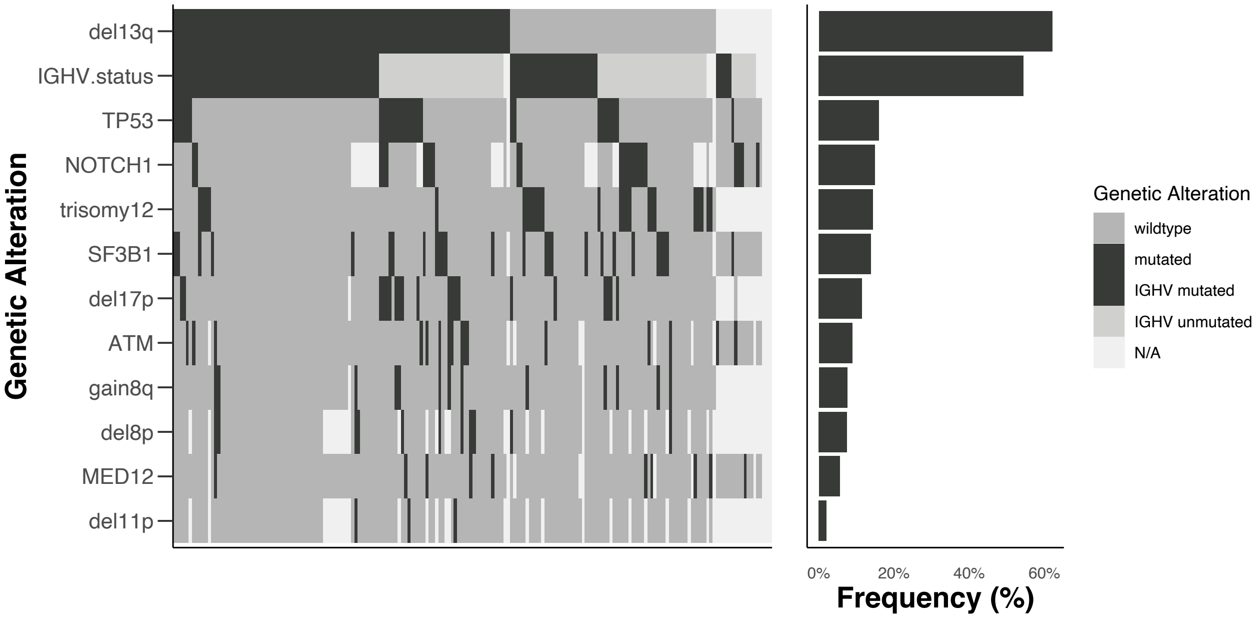

Appendix Figure S1. Genetic profiles of screened patient samples.

Selected genetic features on y-axis and patient samples (n=192) on x-axis.

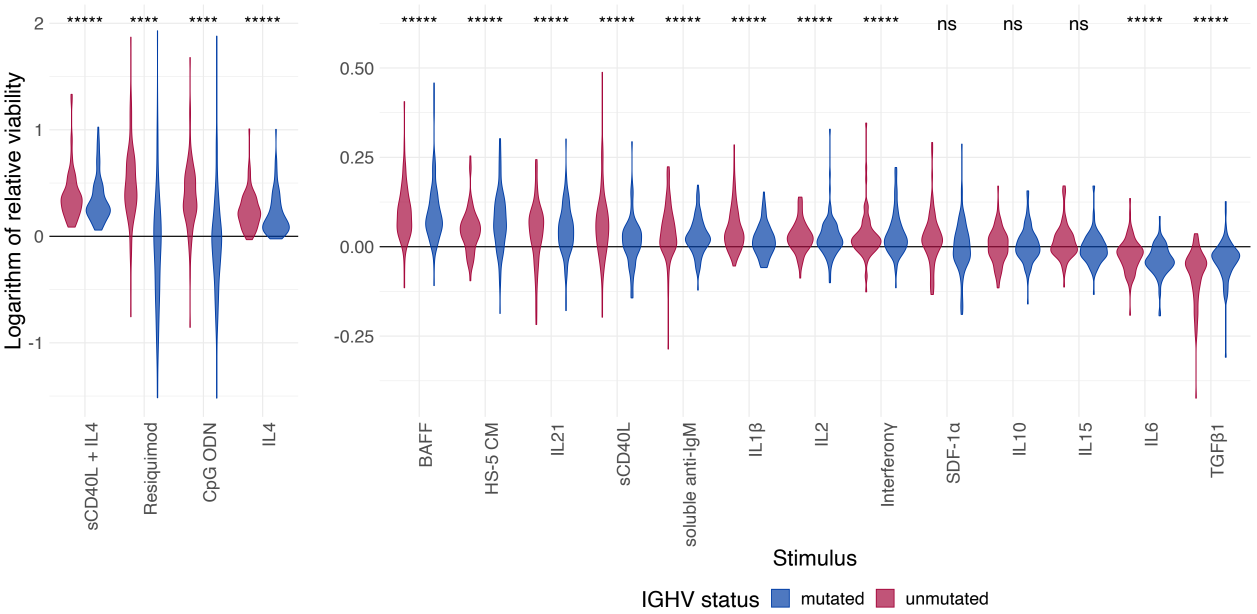

Appendix Figure S2. Response to stimuli stratified by IGHV status.

Logarithm of the relative viability after 48h incubation with microenvironmental stimuli, stratified by IGHV status (IGHV mutated: n=99; IGHV unmutated: n=83). BH-transformed p-values are shown from one-sample Student's t-tests of all patient samples. ($p<0.00001$=*****, $p<0.0001$=****, $p>0.05$=ns)

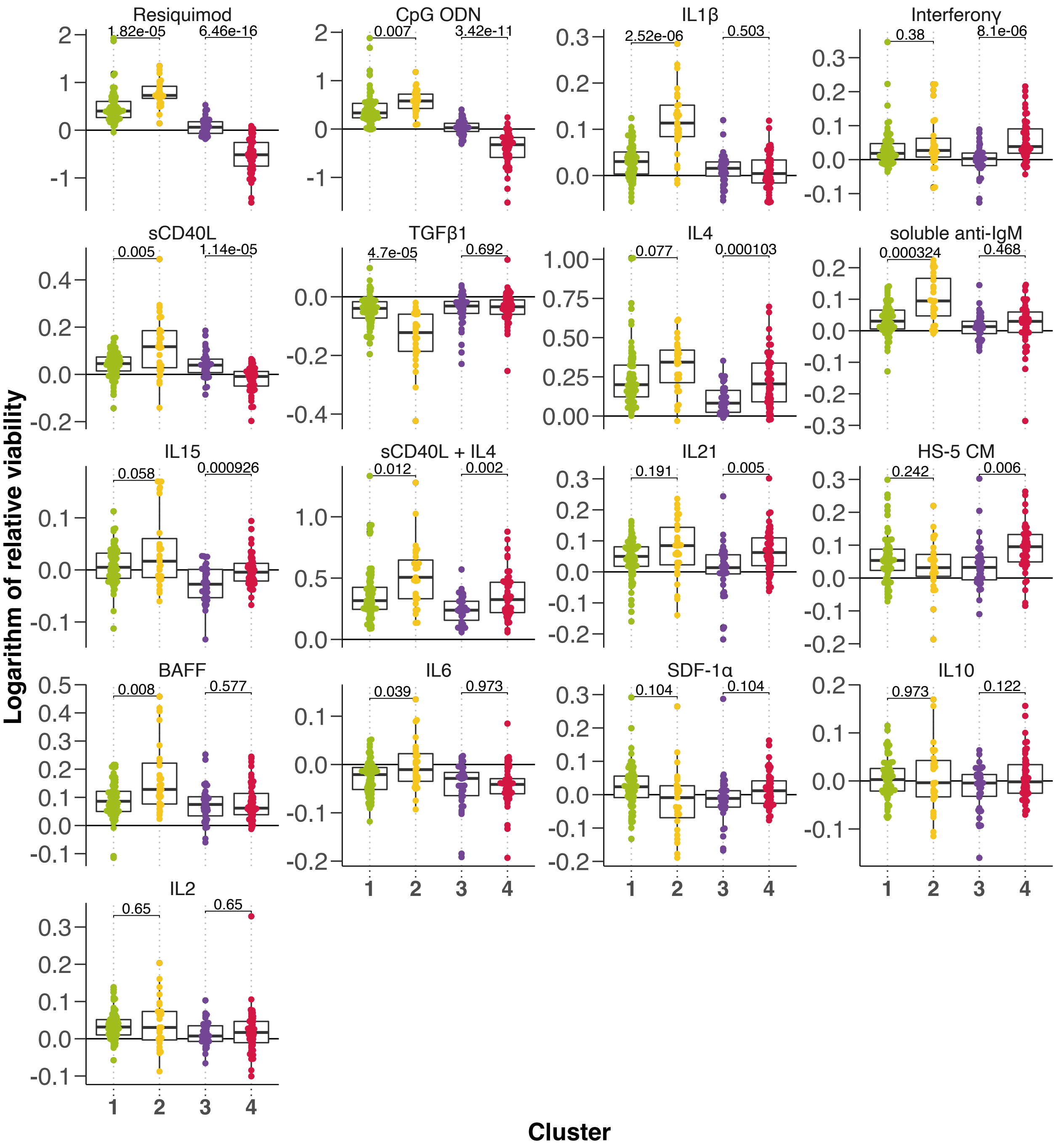

Appendix Figure S3. Stimulus response stratified by clusters.

Logarithm of the relative viability after treatment with stimuli, faceted by cluster. BH-transformed p-values from Student's t-test (C1: n=77; C2:n=27; C3: n=38; C4: n=50).

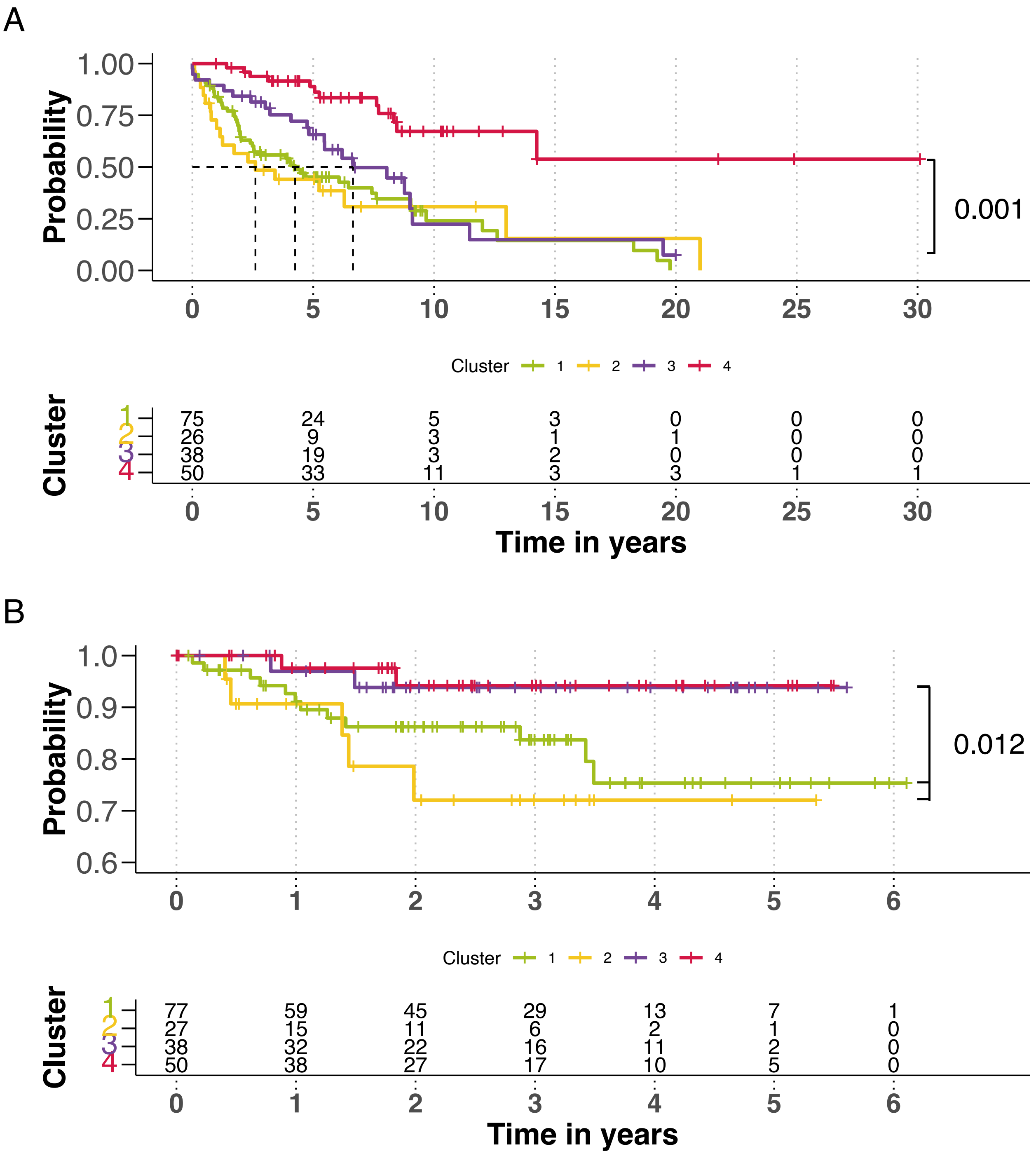

Appendix Figure S4. Time to first treatment and overall survival by clusters.

Kaplan-Meier curves of time to first treatment with p-value from univariate Cox proportional hazards model between C3 and C4 **(A)** and overall survival with p-value from univariate Cox proportional hazards model between C1&2 and C3&4 **(B)**. Median survival not reached.

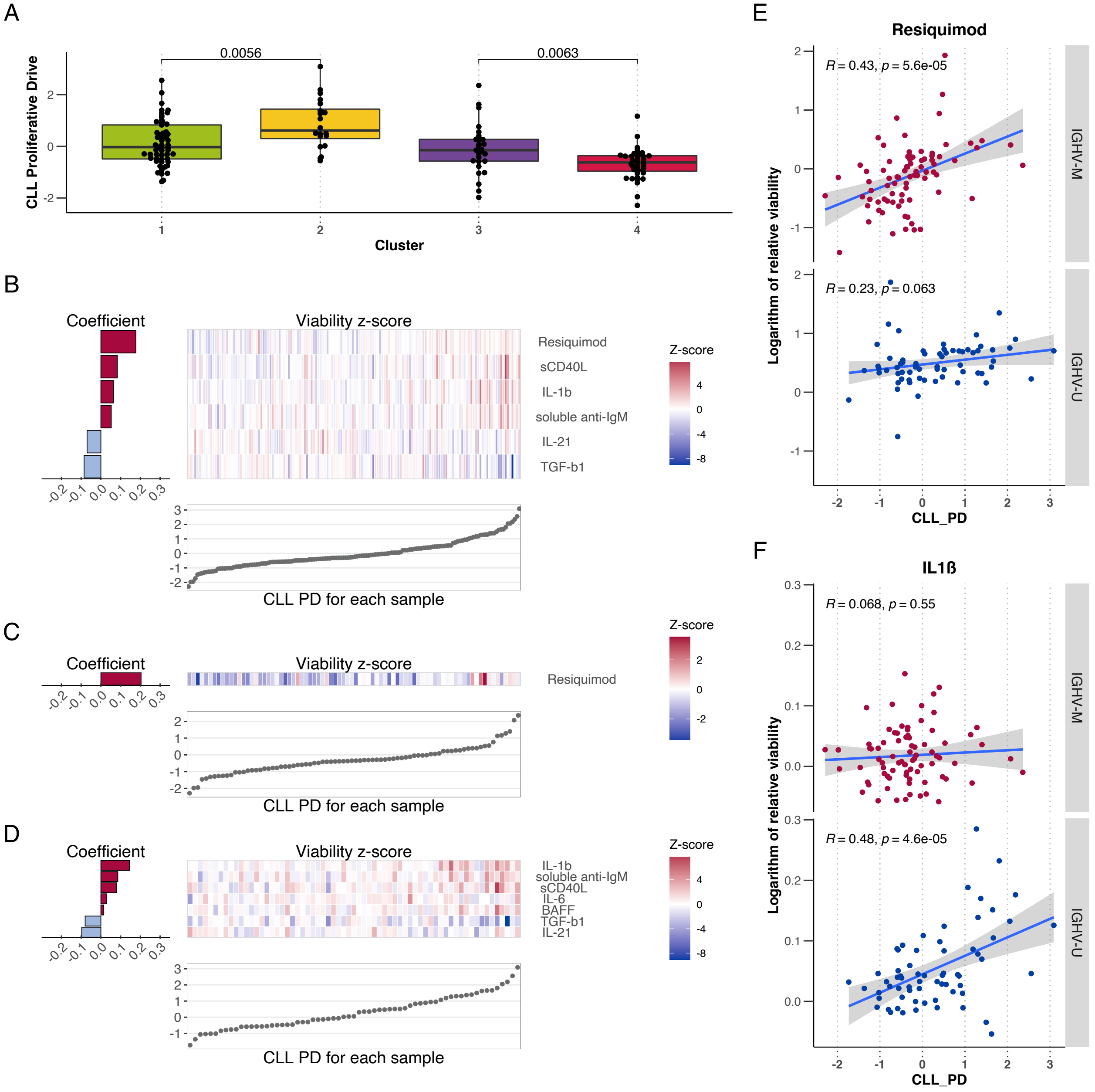

Appendix Figure S5. The relationship between CLL Proliferative Drive (as defined by Lu et al. (2021)[2]) and microenvironmental response.

(A) Beeswarm-boxplot of CLL-PD values stratified by cluster. P-values are from Student's t-tests (C1: n=57; C2:n=20; C3: n=29; C4: n=40). **(B-D)** Predictor profiles depicting relationship between CLL-PD and stimulus response. Stimuli that were associated to the size of CLL-PD were identified with Gaussian linear modelling with L1-penalty, using viability z-scores for each stimulus as feature matrix, and CLL-PD values as response matrix (n=146). Predictor profiles show z-scores of logarithm of relative viability for each stimuli associated with CLL-PD, bar plot on left shows size of coefficient assigned to each stimulus and scatter plot below indicates CLL-PD values for same patient sample. Coefficients are mean of 30 bootstrapped model fits, plotted when selected in >75% of fits. **(C)** shows model outputs for IGHV-M only samples (n=80) and **(D)** for IGHV-U only samples (n=65). **(E & F)** Exemplary scatter plots split by IGHV mutation status shown for Resiquimod **(E)** and IL1ß **(F)** (IGHV-M: n=80, IGHV-U: n=65). CLL-PD on x-axis and the logarithm of

relative viability after microenvironmental stimulation on y-axis. Pearson correlation coefficients are shown with p-values.

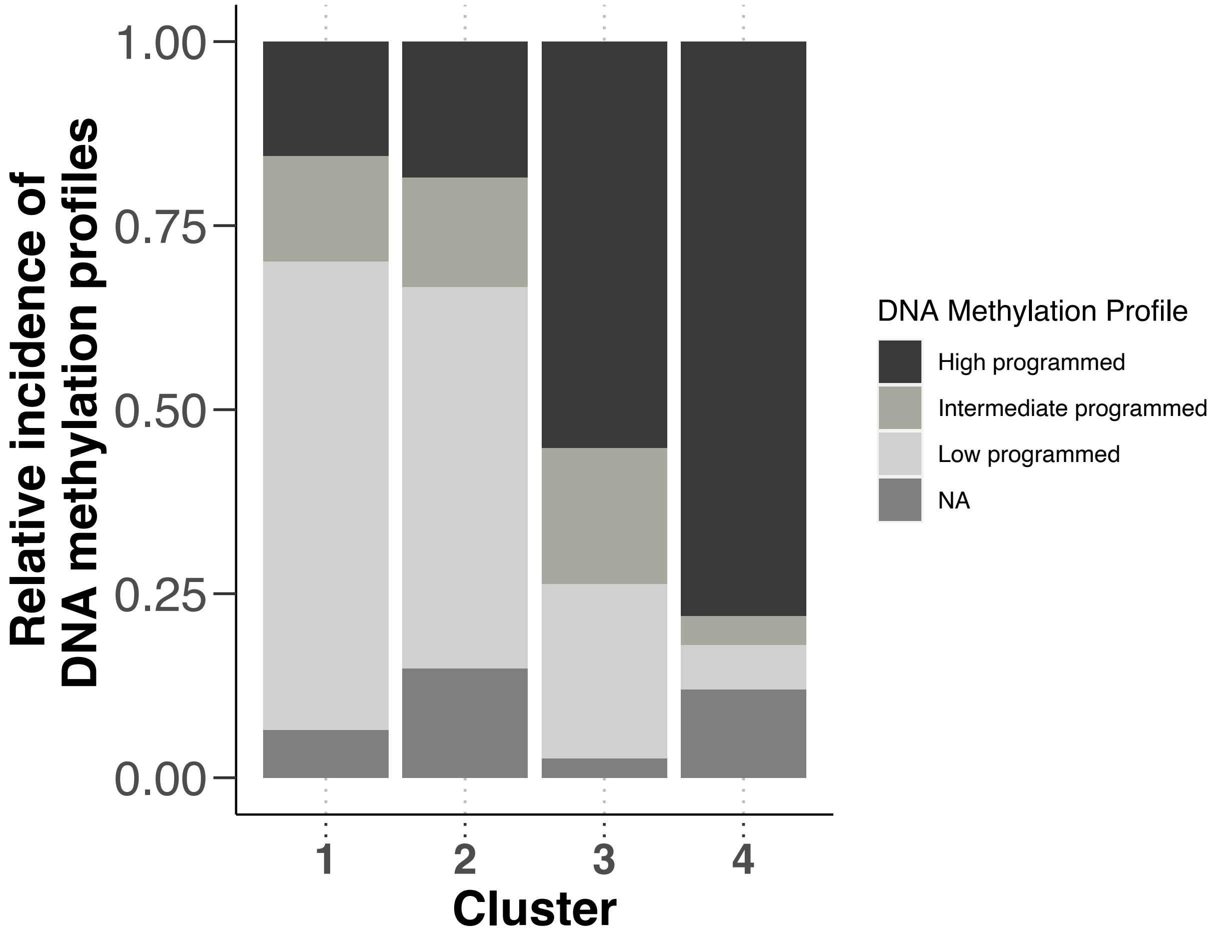

Appendix Figure S6. DNA Methylation profile of Clusters defined by response to microenvironmental stimuli

Barplot of DNA methylation profiles in the four clusters of microenvironmental response. C1 and C2 show higher abundance of low programmed (LP) DNA Methylation profiles, while C3 and C4 show more highly programmed (HP) DNA methylation clusters (C1: n=77; C2:n=27; C3: n=38; C4: n=50). NA=Not available.

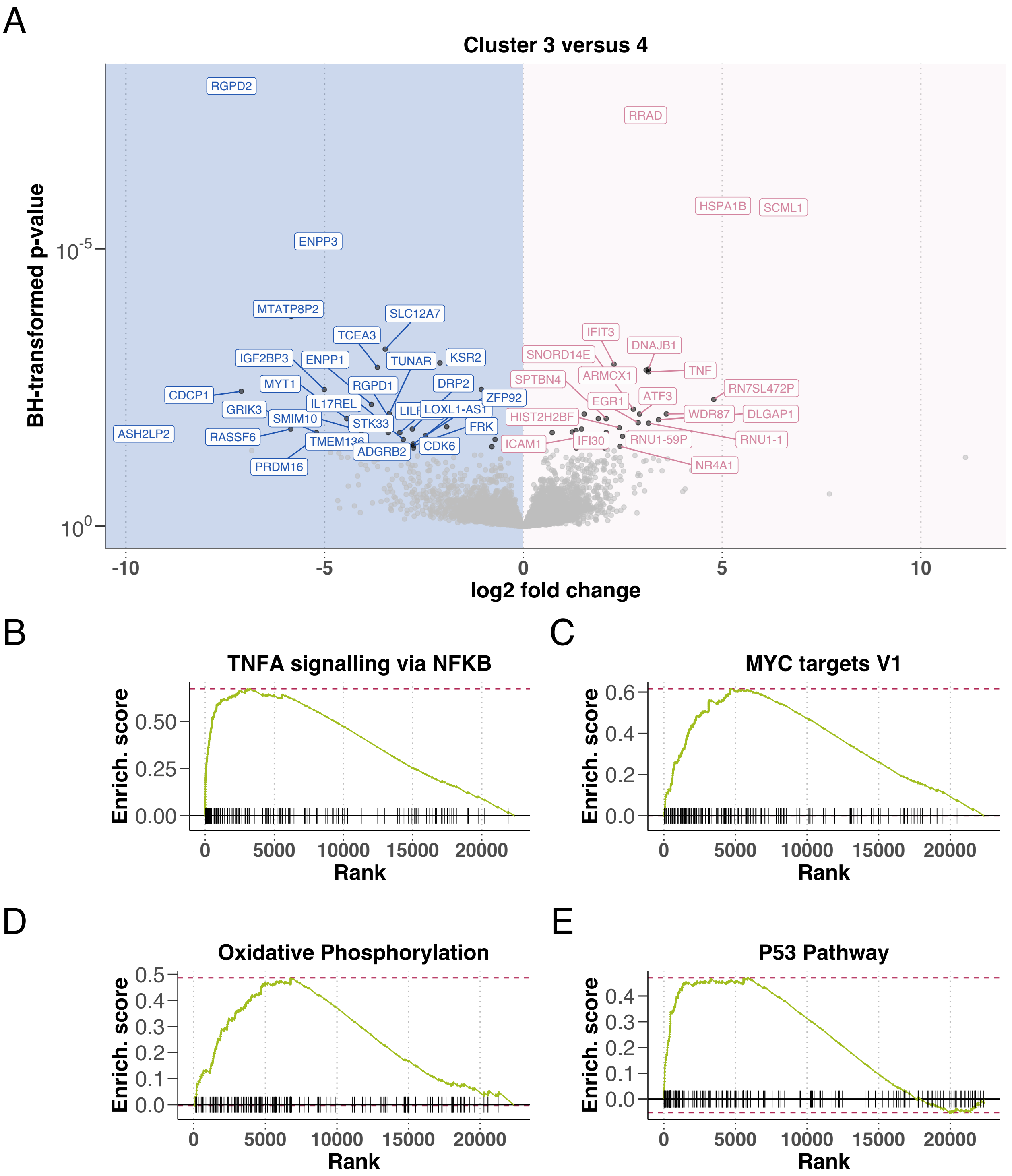
A
Cluster 3 versus 4
BH-transformed p-value
log2 fold change
RGPD2
RRAD
HSPA1B
SCML1
ENPP3
MTATP8P2
SLC12A7
TCEA3
IFIT3
DNAJB1
IGF2BP3
ENPP1
TUNAR
KSR2
SNORD14E
TNF
ARMCX1
SPTBN4
CDCP1
MYT1
RGPD1
DRP2
ATF3
RN7SL472P
IL17REL
ZFP92
EGR1
GRIK3
LOXL1-AS1
HIST2H2BF
WDR87
DLGAP1
SMIM10
STK33
FRK
ASH2LP2
RASSF6
ICAM1
IFI30
RNU1-59P
RNU1-1
TMEM136
ADGRB2
CDK6
NR4A1
PRDM16
B
TNFA signalling via NFKB
C
MYC targets V1
D
Oxidative Phosphorylation
E
P53 Pathway
Enrich. score
Rank

Appendix Figure S7. RNA-Sequencing of matched samples indicates differential gene expression between Cluster 3 and Cluster 4.

Volcano plot of differentially expressed genes between Cluster 3 and Cluster 4 (C3: n=9; C4: n=12) **(A)**. X axis indicates log2 fold change values, calculated using the DESeq2 package, y axis gives corresponding -log10(transformed p-value). P-values transformed using BH method. Genes are labeled where p-value < 0.05. Enrichment plots of selected pathways **(B-D)**. Gene set enrichment analysis (GSEA) was performed with the Hallmark gene sets from the GSEA Molecular Signatures Database. Wald statistic was used to rank the genes. The green curve corresponds to the Enrichment Score curve, which is the running sum of the weighted enrichment score obtained from GSEA software.

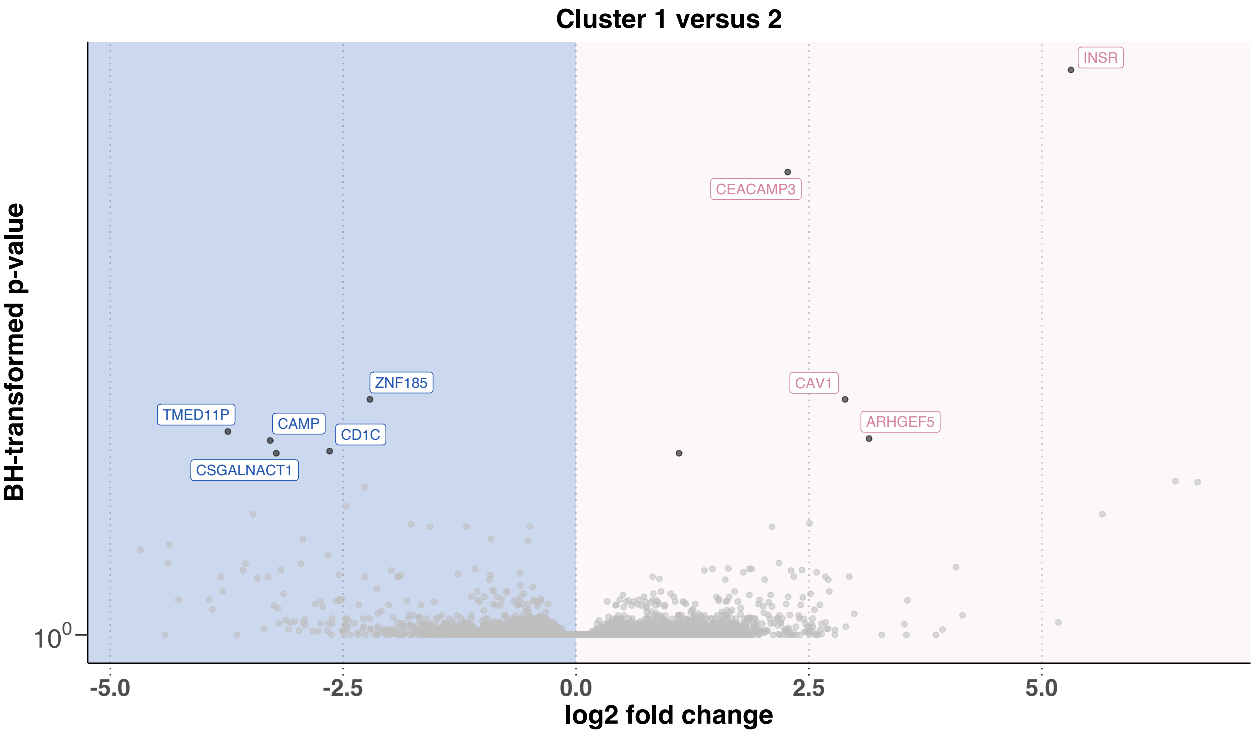

Appendix Figure S8. RNA-Sequencing of matched samples indicates differential gene expression between Cluster 1 and 2.

Volcano plot of differentially expressed genes between Cluster 1 and Cluster 2 (C1: n=17; C2: n=11). X axis indicates log2 fold change values, calculated using the DESeq package, y axis gives corresponding -log10(transformed p-value). P-values transformed using BH method. Genes are labeled where p-value<0.05.

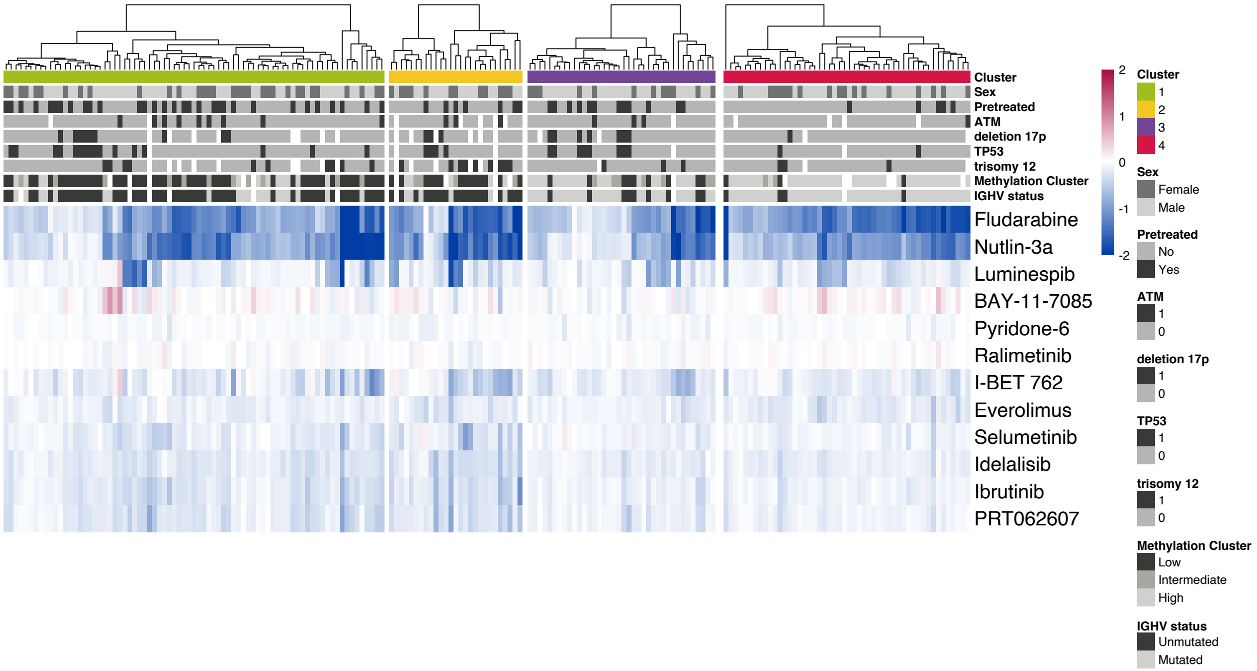

Appendix Figure S9. Drug response between clusters.

Heatmap showing the logarithm of relative viability after drug treatment. Rows represent drug treatments and columns represent primary CLL samples (n=192), annotated for their genetic background, sex and pretreatment status above. Red values indicate increased viability upon treatment, blue indicates decreased viability.

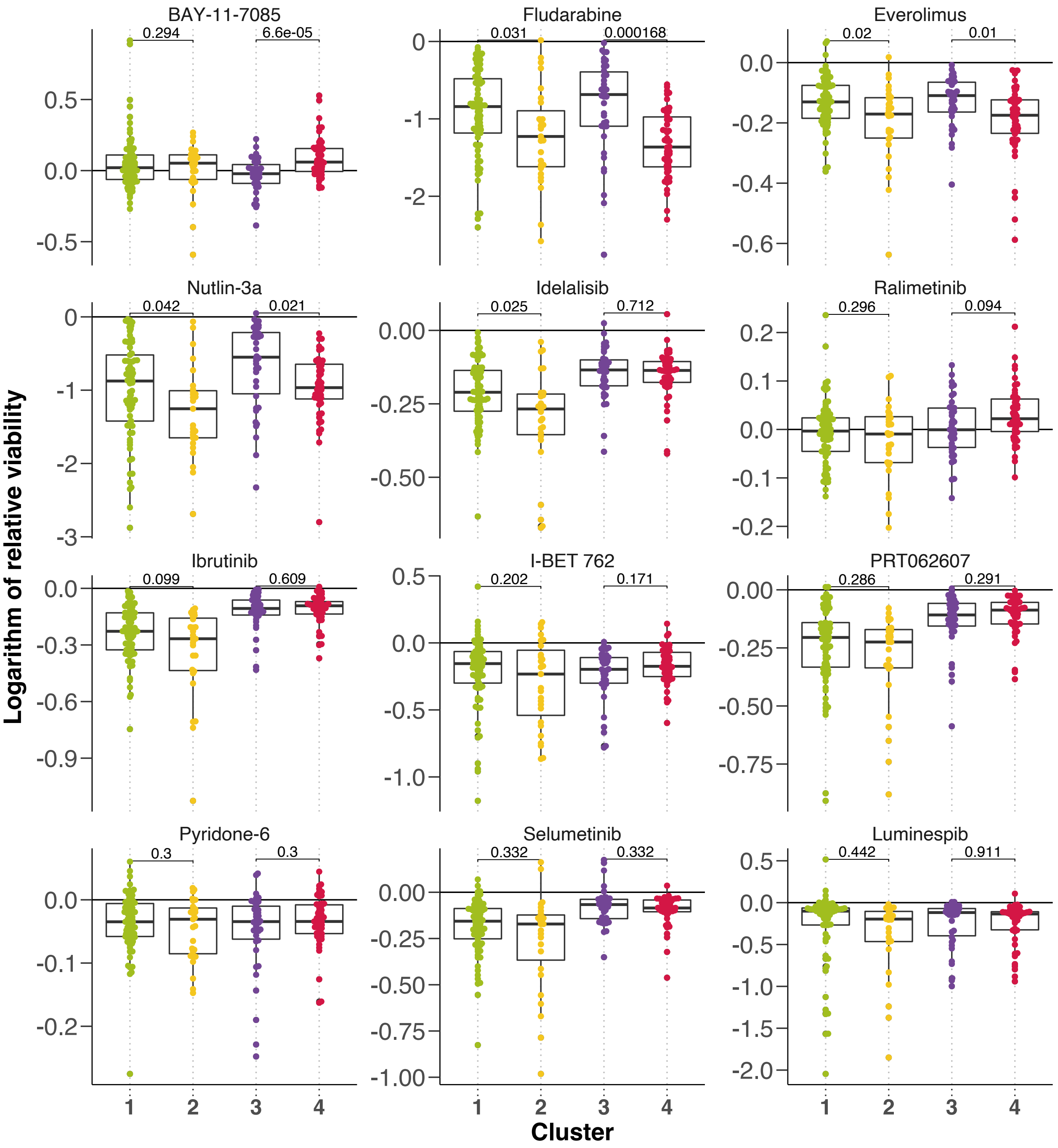

Appendix Figure S10. Drug response by clusters.

Logarithm of the relative viability after treatment with drugs, faceted by cluster. BH-transformed p-values from non-paired, two-sided Student's t-test are shown (C1: n=77; C2:n=27; C3: n=38; C4: n=50).

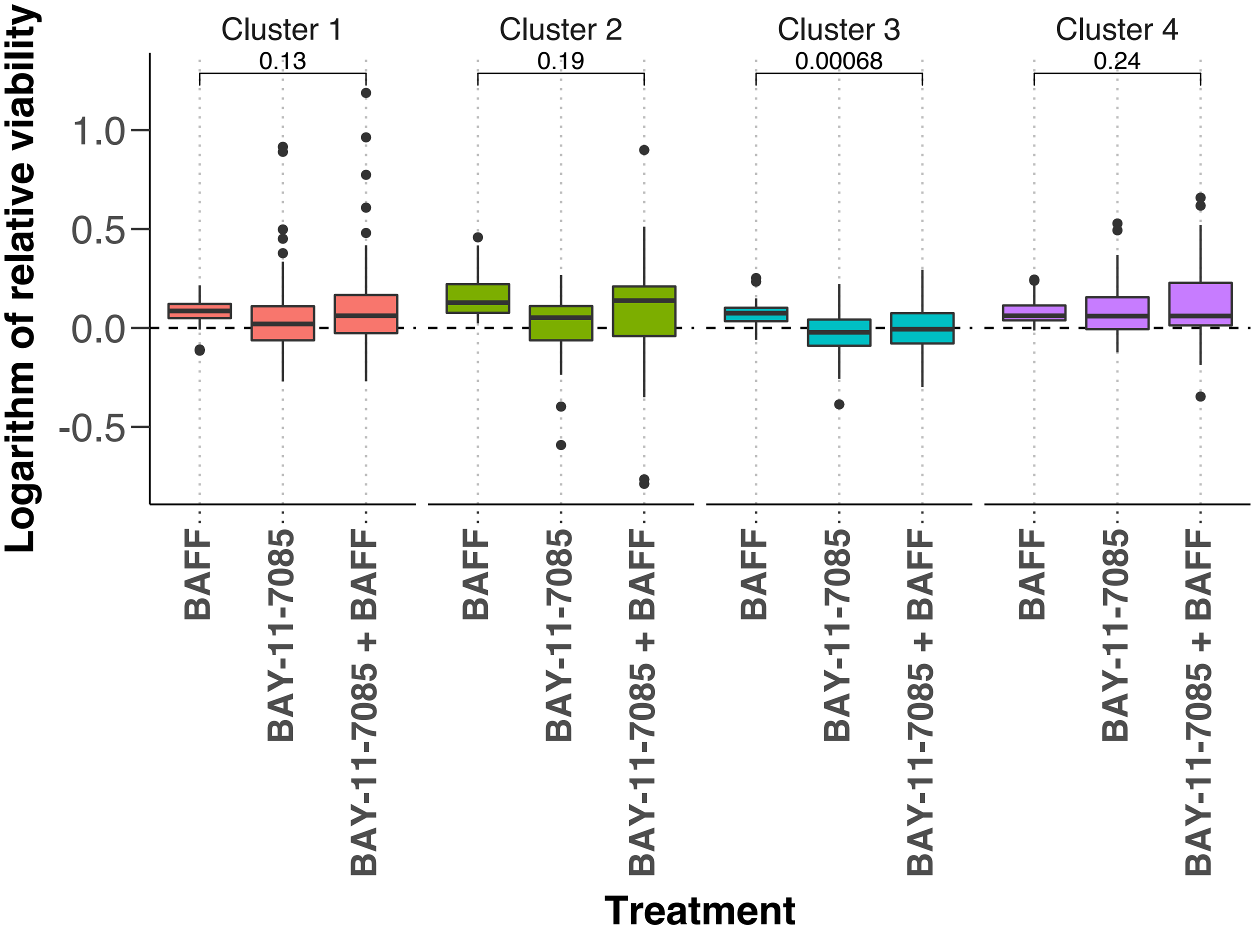

Appendix Figure S11. Effect of BAY-11-7085 treatment on BAFF response

Logarithm of the relative viability after treatment BAFF, BAY-11-7085 or both, faceted by cluster. P-values from paired, two-sided Student's t-test are shown (C1: n=77; C2:n=27; C3: n=38; C4: n=50).

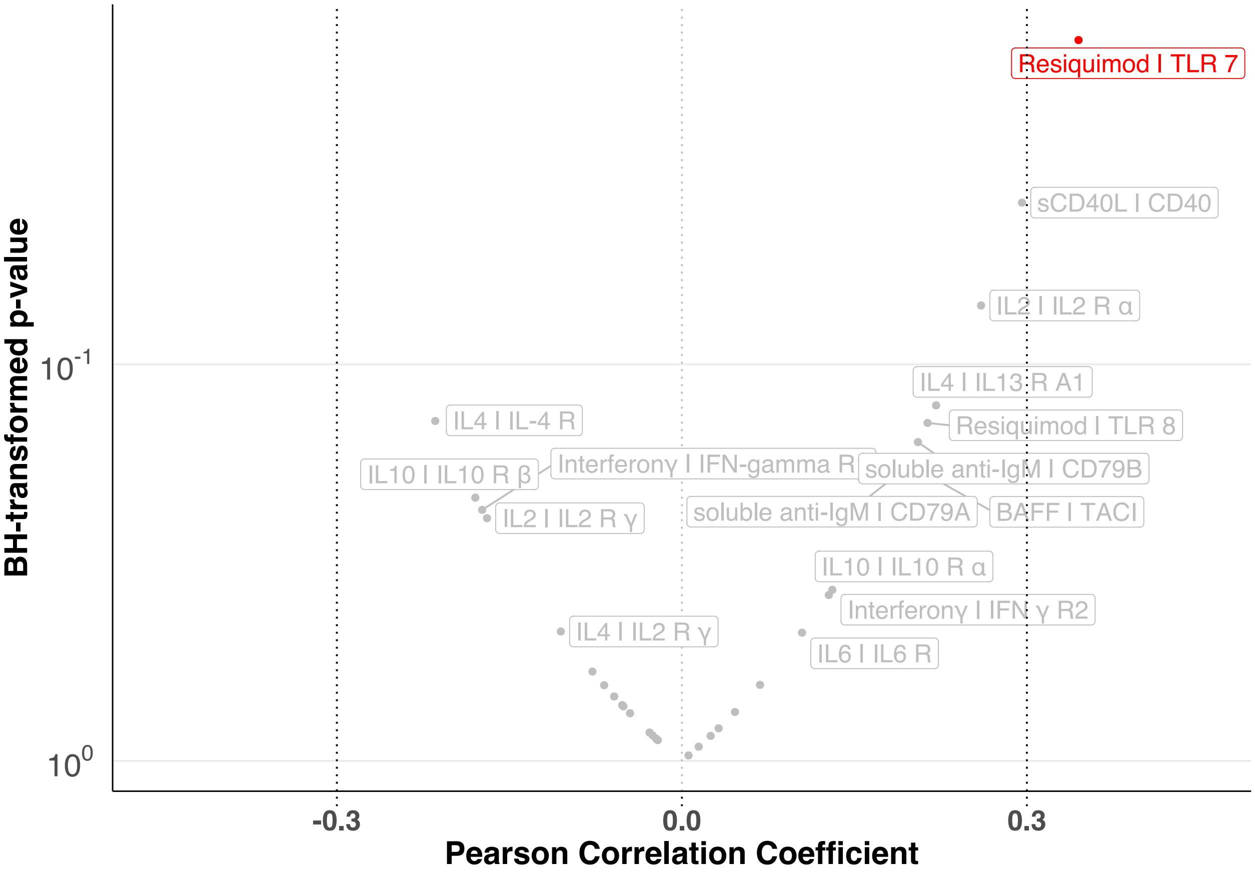

Appendix Figure S12. Correlation of stimulus response and receptor expression.

Volcano plot depicts Pearson correlation coefficients against corresponding BH-transformed p-values, for the correlation between the logarithm of the relative viability and vst RNA counts of corresponding stimulus receptor (n=49). RNA counts taken from RNA-Sequencing of untreated matched CLL patient sample. Only viability after treatment with Resiquimod correlated with receptor expression (R>0.3).

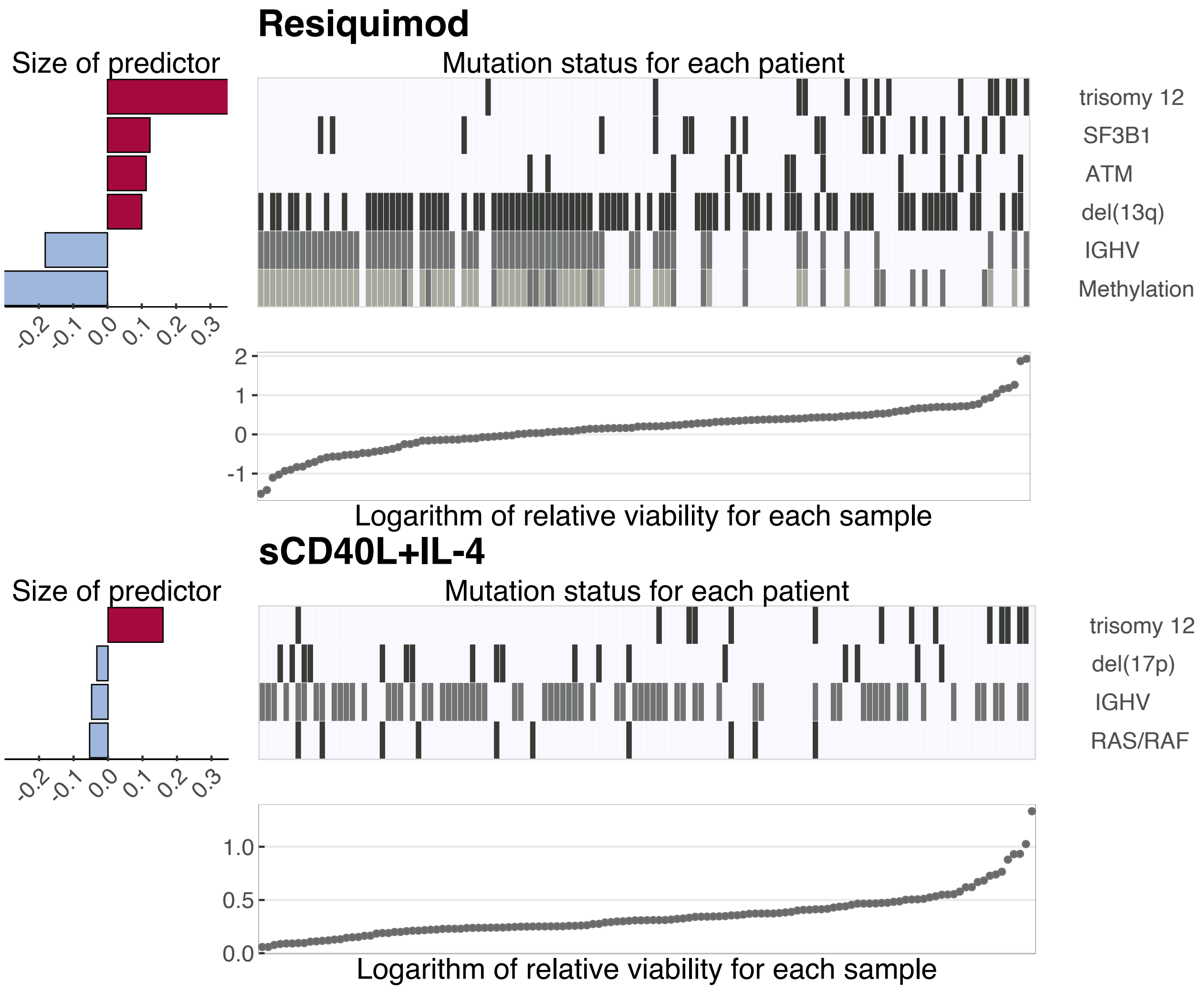

Appendix Figure S13. Predictor profiles to represent genetic feature-stimulus associations.

Stimuli which are not shown in Figure 3B but have predictors >0.02 assigned by multivariate analysis using Gaussian linear modelling with L1-penalty are shown. Within the plots, the bar plots on left indicate size and sign of coefficients assigned to the named predictors. Positive coefficients indicate higher viability after stimulation if the feature is present. Scatter plots indicate the logarithm of relative viability values, in order of magnitude, for each individual sample. Heatmaps show status for each of the genetic predictors for corresponding sample in scatter plot (n=128).

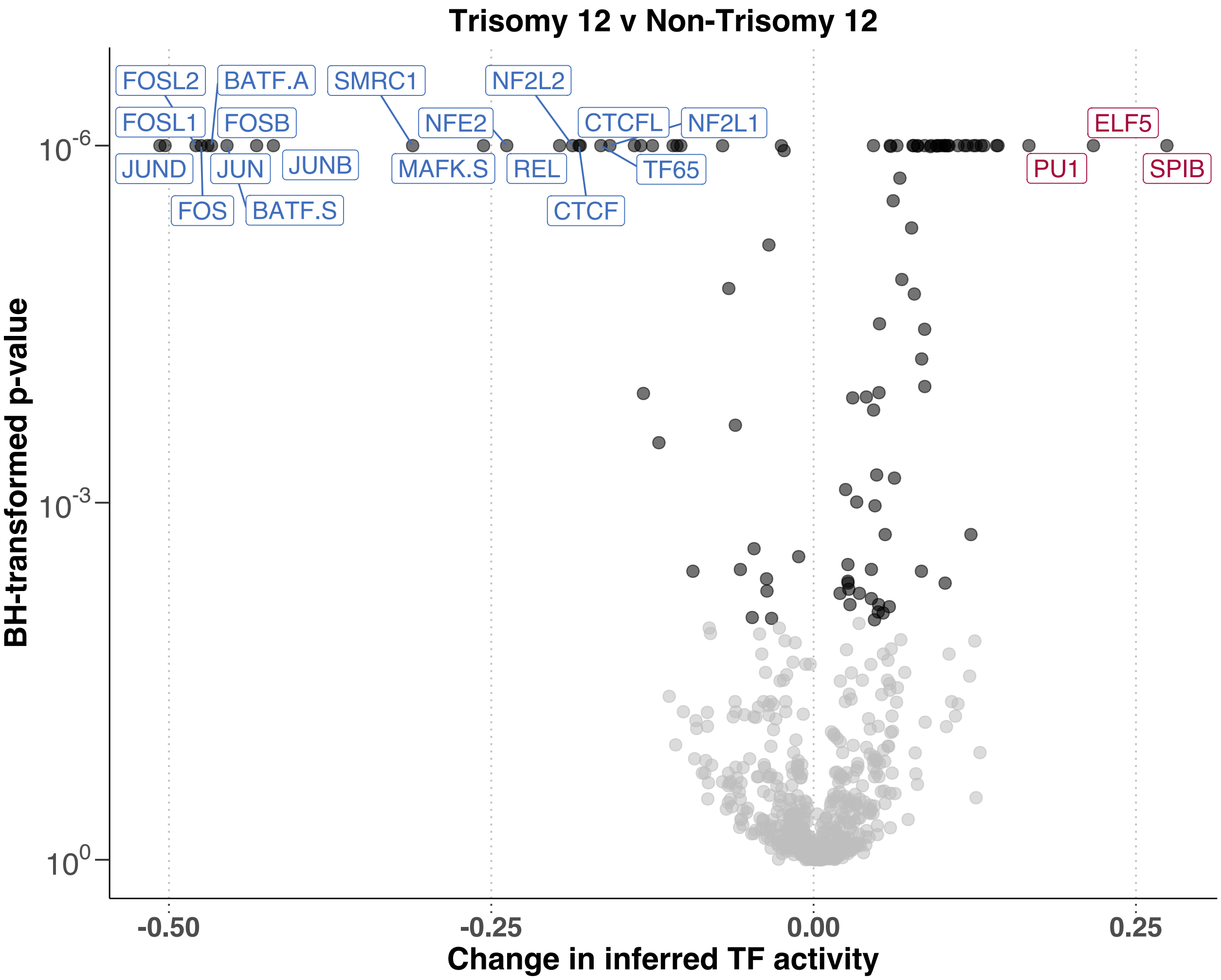

Appendix Figure S14. ATACseq comparing trisomy 12 and non-trisomy 12 CLL samples.

Volcano plot shows change in inferred TF activity (x-axis) against transformed p-values (y-axis) for trisomy 12 (n = 2) versus non-trisomy 12 samples (n = 2). Inferred TF activity calculated using the diffTF package, TFs are labeled where inferred activity > 0.15, and BH-transformed p-value < 0.01.

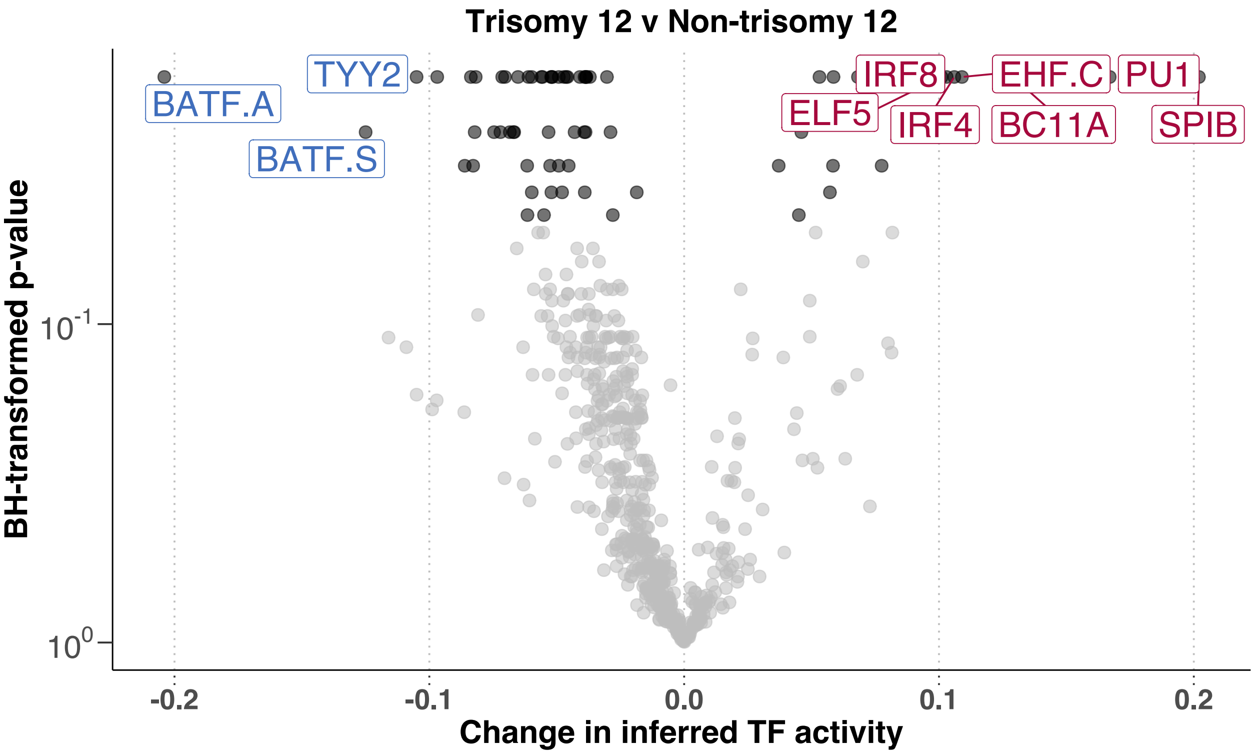

Appendix Figure S15. ATACseq comparing trisomy 12 and non-trisomy 12 CLL samples.

Volcano plot shows change in inferred TF activity (x-axis) against transformed p-values (y-axis) for trisomy 12 (n = 13) versus non-trisomy 12 samples (n = 87). Inferred TF activity calculated using the diffTF package, and measured as weighted mean difference. p-values are obtained through diffTF in permutation mode and transformed by the Benjamini-Hochberg procedure. TFs are labeled where absolute weighted mean difference > 0.1, and transformed p-value < 0.05. Data from Beekman et al. 2018[4].

A

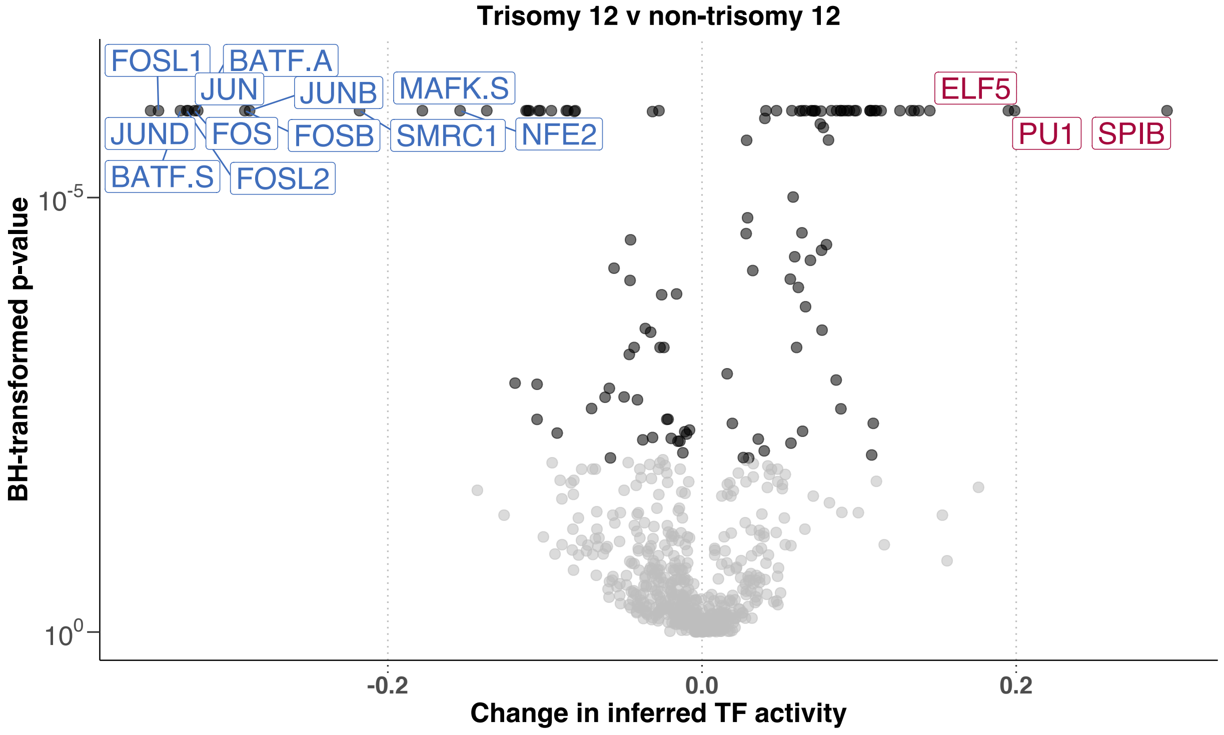
Trisomy 12 v non-trisomy 12
FOSL1
BATF.A
JUN
JUNB
MAFK.S
ELF5
JUND
FOS
FOSB
SMRC1
NFE2
PU1
SPIB
BATF.S
FOSL2
BH-transformed p-value
10^{-5}
10^{0}
-0.2
0.0
0.2
Change in inferred TF activity

B

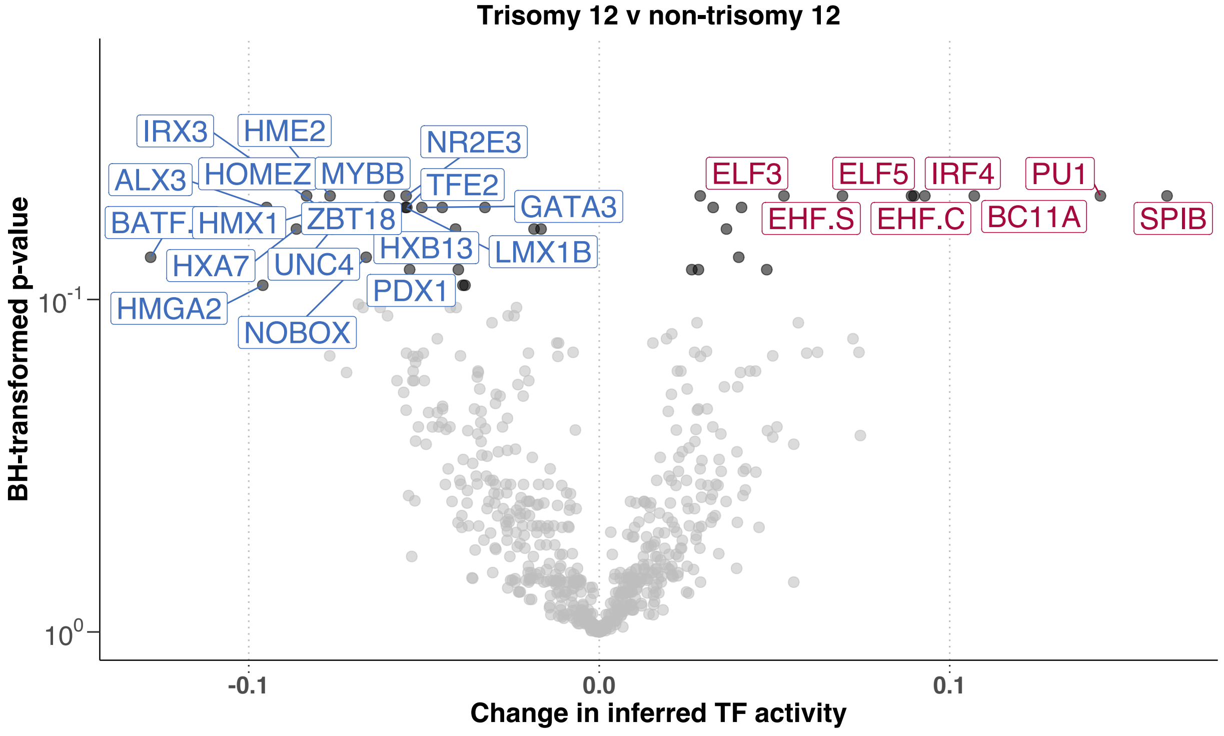
Trisomy 12 v non-trisomy 12
IRX3
HME2
NR2E3
ALX3
HOMEZ
MYBB
TFE2
ELF3
ELF5
IRF4
PU1
GATA3
BATF
HMX1
ZBT18
EHF.S
EHF.C
BC11A
SPIB
HXB13
LMX1B
HXA7
UNC4
PDX1
HMGA2
NOBOX
BH-transformed p-value
10^{-1}
10^{0}
-0.1
0.0
0.1
Change in inferred TF activity

Appendix Figure S16. ATACseq comparing trisomy 12 and non-trisomy 12 CLL samples, independent of third copy of chromosome 12

Volcano plot shows change in inferred TF activity (x axis) against transformed p-values (y axis) for trisomy 12 versus non-trisomy 12 samples, for (A) the 2 versus 2 comparison and (B) the 9 versus 42 comparison based on the data from Rendeiro et al. 2016[3]. Input peak files supplied to diffTF did not include peaks on chromosome 12. p-values are obtained through diffTF in (A) analytic mode and (B) permutation mode, transformed by the Benjamini-Hochberg procedure. TFs are labelled in (A) where absolute weighted mean difference > 0.15, and transformed p-value < 0.01 and in (B) where absolute weighted mean difference > 0.05, and transformed p-value < 0.1.

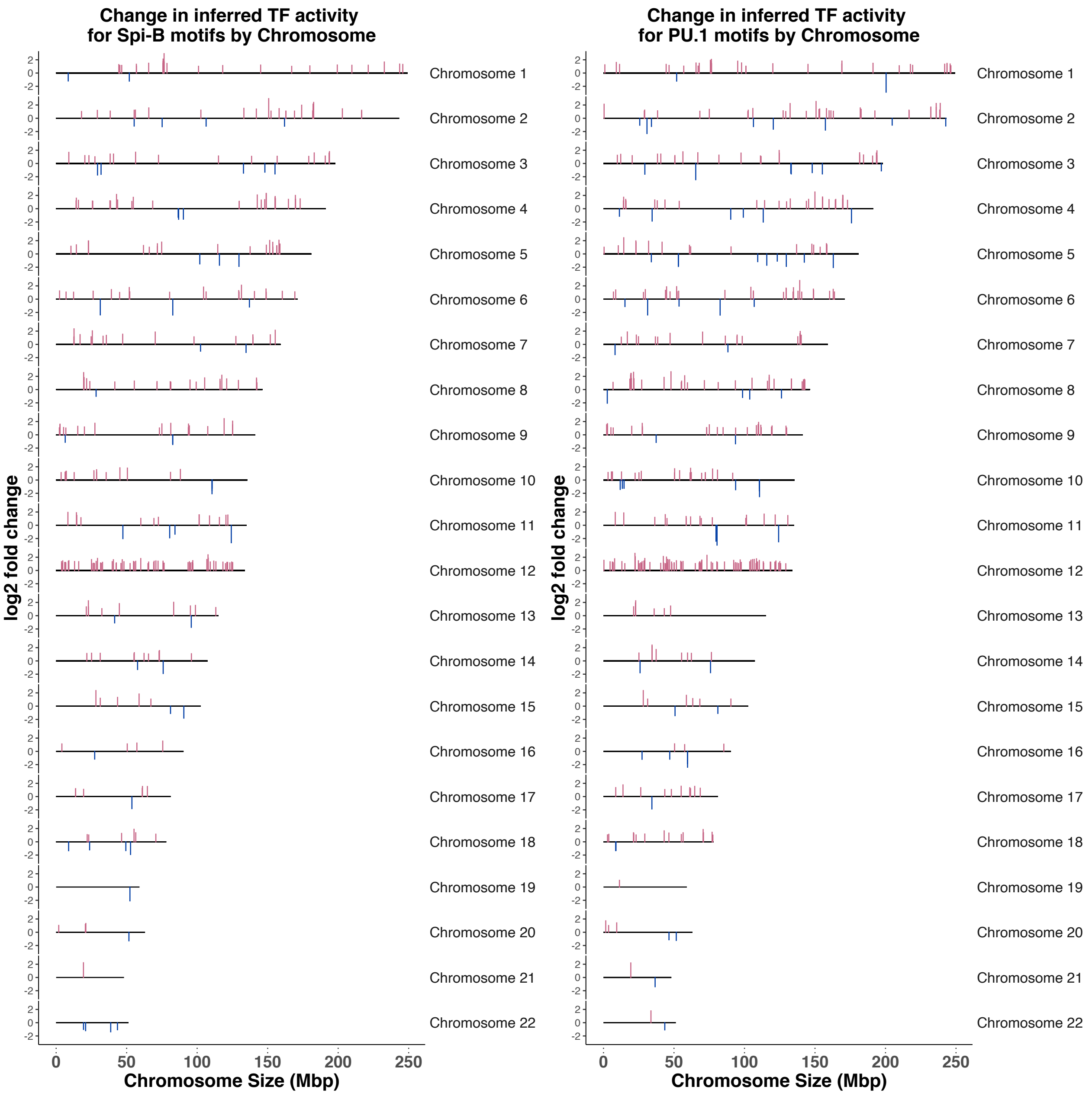

Appendix Figure S17. Chromosomal locations of Spi-B and PU.1 motifs that show differential accessibility between trisomy 12 and non-trisomy 12 CLL.

Log2 fold changes are plotted (y axis) for Spi-B and PU.1 motifs that show an absolute Log2 fold change > 1 and a p-value < 0.1, as calculated using the diffTF package for all Spi-B and PU.1 motif locations, (motif defined in the HOCOMOCO database). Data shown is the 9 (trisomy 12 CLL) versus 42 (non-trisomy 12 CLL) comparison based on the data from Rendeiro et al. 2016[3].

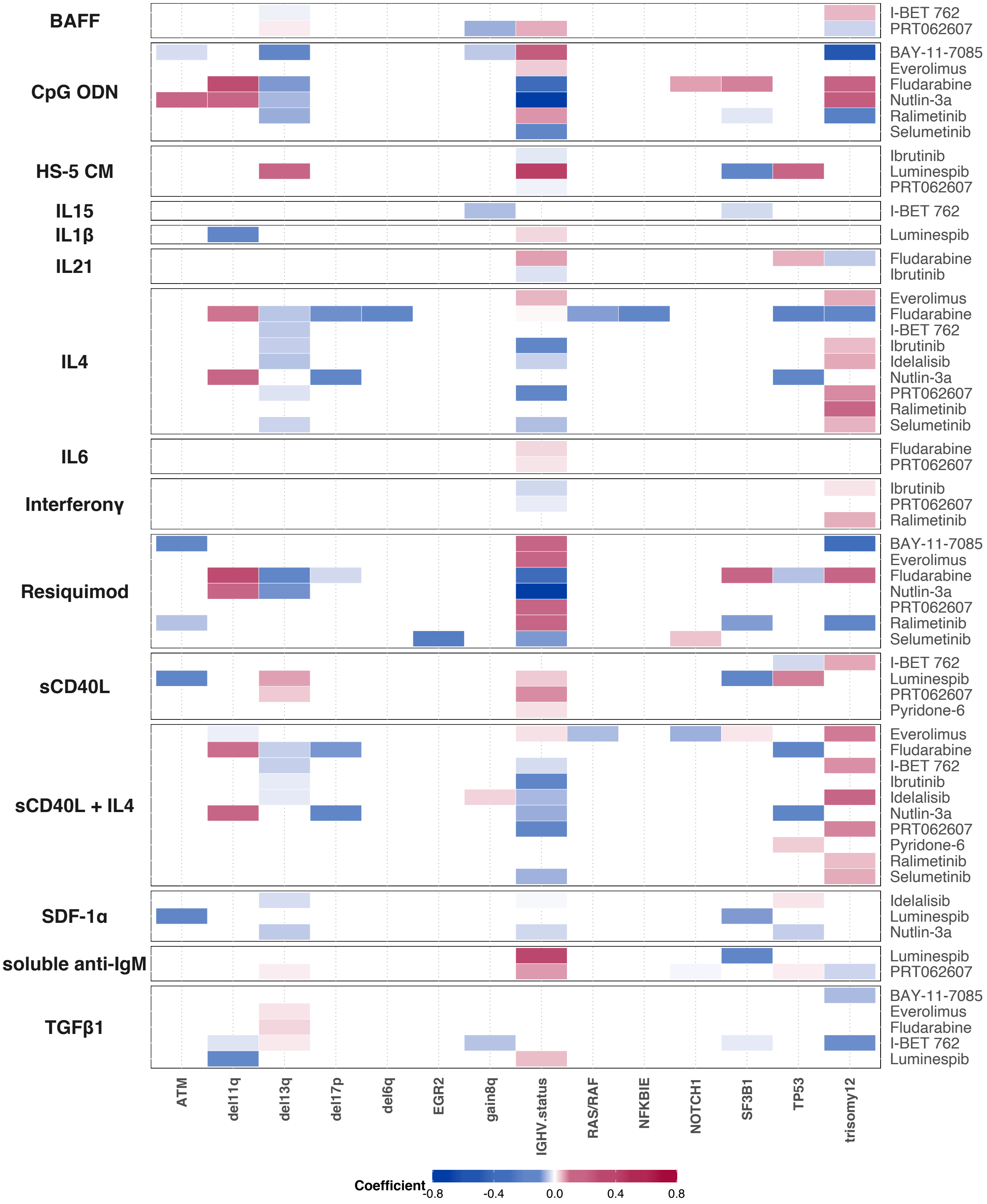

Appendix Figure S18. Genetic predictors of drug-stimulus interactions.

Based on the drug-stimulus viability data of 137 CLL patient samples with complete genetic annotation, we fitted a sample specific linear model. Heatmap depicting overview of genetic predictors of drug - stimulus interactions (each row represents the coefficients from fitting a single multivariate model). Stimuli are shown

on left, and corresponding drugs on right. Drugs, stimuli and genetic alterations are alphabetically sorted. Coloured fields indicate that the interaction coefficient for given drug and stimulus is modulated by corresponding genetic feature. Positive coefficients are shown in red, indicating the interaction coefficient is more positive for given drug and stimulus combination if the feature is present.

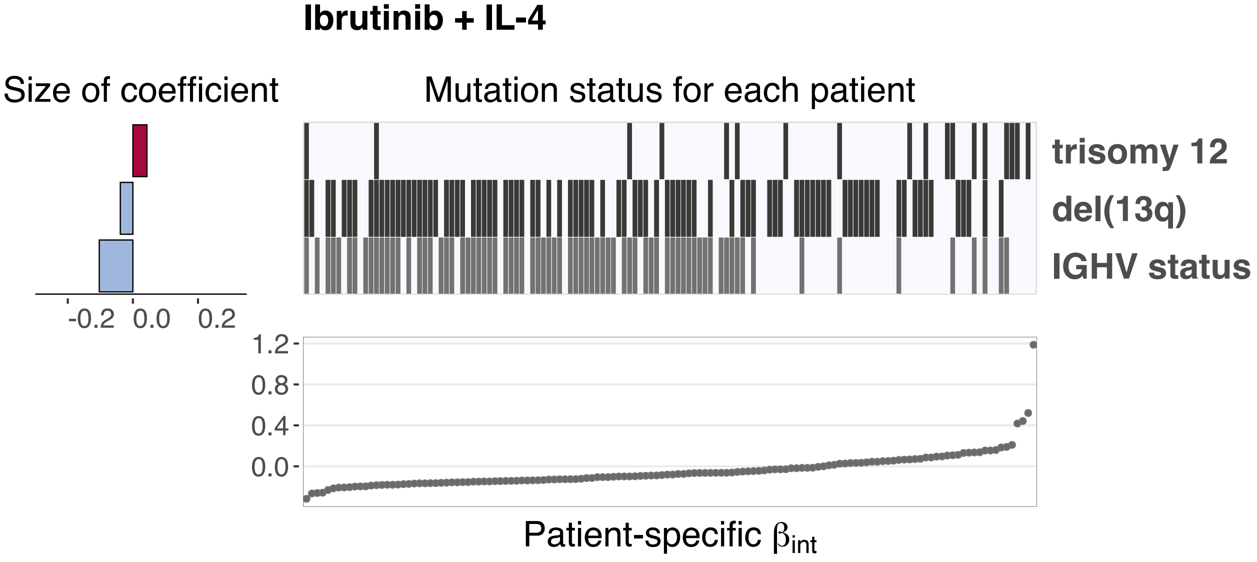

Appendix Figure S19. Genetic predictors of the interaction between ibrutinib and IL4.

Predictor profile depicting genetic features that modulate the size of the β_{int} for ibrutinib and IL4. To generate the predictor profile, linear model in Equation 1 was fitted in a sample-specific manner, to calculate drug-stimulus β_{int} s for each patient sample (n=137). Ranked patient-specific β_{int} values are shown in lower scatter plot. Associations between the size of the β_{int} and genetic features were identified using multivariate regression with L1 (lasso) regularisation, with gene mutations and IGHV status as features, and selecting coefficients that were chosen in >90% of bootstrapped model fits. The horizontal bars on left show the size of fitted coefficients assigned to genetic features. Matrix above scatter plot indicates patient mutation status for the selected genetic features. Matrix fields correspond to points in scatter plot (i.e. patient data is aligned), to indicate how the size of the β_{int} varies with selected genetic feature.

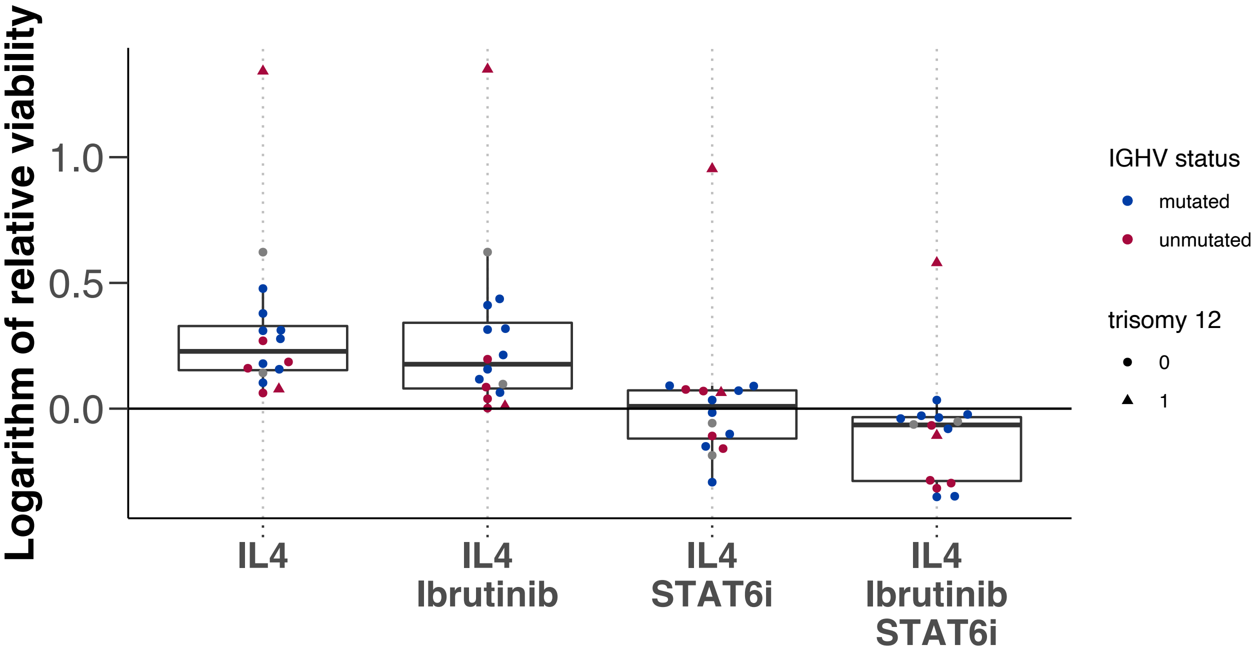

Appendix Figure S20. STAT6 dependency of IL4 signaling.

Natural logarithm of the relative viability after treatment with IL4 in combination with ibrutinib, the STAT6 inhibitor AS1517499, and both (n=16). The effects observed with IL4 stimulation are dependent on STAT6 activation. Addition of the STAT6 inhibitor AS1517499 could revoke the effect on baseline viability as well as drug induced toxicity.

References

1. Care, M.A., Cocco, M., Laye, J.P., et al. (2014) SPIB and BATF provide alternate determinants of IRF4 occupancy in diffuse large B-cell lymphoma linked to disease heterogeneity. Nucleic Acids Res 42: 7591–7610

2. Lu, J., Cannizzaro, E., Meier-Abt, F. et al. Multi-omics reveals clinically relevant proliferative drive associated with mTOR-MYC-OXPHOS activity in chronic lymphocytic leukemia. Nat Cancer 2, 853–864 (2021).

3. Rendeiro, A., Schmidl, C., Strefford, J. et al. Chromatin accessibility maps of chronic lymphocytic leukaemia identify subtype-specific epigenome signatures and transcription regulatory networks. Nat Commun 7, 11938 (2016).

4. Beekman, R., Chapaprieta, V., Russiñol, N. et al. The reference epigenome and regulatory chromatin landscape of chronic lymphocytic leukemia. Nat Med 24, 868–880 (2018).
