# Supplementary material for: Drug‐microenvironment perturbations reveal resistance mechanisms and prognostic subgroups in CLL
Source: Mol Syst Biol. 2022 Aug 12;18(8):e10855. doi: 10.15252/msb.202110855 (PMC9372727; doi:10.15252/msb.202110855)
Supplement: Supplementary file 3 [file MSB-18-e10855-s001.pdf]

molecular systems biology

Drug-microenvironment perturbations reveal resistance mechanisms and prognostic subgroups in CLL

Peter-Martin Bruch, Holly Giles, Carolin Kolb, Sophie Herbst, Tina Becirovic, Tobias Roider, Junyan Lu, Sebastian Scheinost, Lena Wagner, Jennifer Huellein, Ivan Berest, Mark Kriegsmann, Katharina Kriegsmann, Christiane Zgorzelski, Peter Dreger, Judith Zaugg, Carsten Müller-Tidow, Thorsten Zenz, Wolfgang Huber, and Sascha Dietrich
DOI: 10.15252/msb.202110855

Corresponding author(s): Sascha Dietrich (sascha.dietrich@embl.de) , Wolfgang Huber (wolfgang.huber@embl.org)

Review Timeline:

Submission Date:	6th Dec 21
Editorial Decision:	11th Jan 22
Revision Received:	11th May 22
Editorial Decision:	8th Jun 22
Revision Received:	14th Jul 22
Accepted:	22nd Jul 22

Editor: Maria Polychronidou

Transaction Report:

(Note: With the exception of the correction of typographical or spelling errors that could be a source of ambiguity, letters and reports are not edited. Depending on transfer agreements, referee reports obtained elsewhere may or may not be included in this compilation. Referee reports are anonymous unless the Referee chooses to sign their reports.)

1st Editorial Decision — 11th Jan 2022

Thank you again for submitting your work to Molecular Systems Biology. We have now heard back from the three referees who agreed to evaluate your study. Overall, the reviewers acknowledge that the study seems relevant for the field. They raise however a series of concerns, which we would ask you to address in a major revision.

I think that the reviewers' recommendations are rather clear and therefore there is no need to repeat the points listed below. The reviewers mainly refer to the need to better support the main conclusions (e.g. by including additional validatory analyses) and they recommend improving the data presentation and interpretation.

All issues raised by the reviewers need to be satisfactorily addressed. Please contact me in case you would like to discuss in further detail any of the issues raised.

On a more editorial level, we would ask you to address the following points:

Reviewer #1:

Summary:
The manuscript of Bruch/Giles et al. describes an approach that tests microenvironmental stimuli on CLL viability and drug responses in 192 genetically characterized patient samples ex vivo. Additional available data of the cohort is used to connect genotype to drug responses/environmental stimuli. The authors show that they can classify CLL patients based on environmental stimuli responses, that the subgroups show distinct clinical characteristics, and that trisomy 12 seems to be a driver to response to such stimuli. Further Trisomy 12 is linked to a specific chromatin signature that might be targetable by BRD inhibition. As another key finding, the authors report in detail IL4 and TLR activity as the strongest stimuli, and IFN signaling as a new stimulus, which mediate potential treatment resistance. The data should facilitate understanding interactions between drugs and the microenvironment.

General remarks:
The paper is well written, and all data is available for interactive exploration. Although the ex vivo testing excludes the effect of drugs on the microenvironment cells, the idea to systematically test interactions of such environmental stimuli with drugs is very appealing. In light of the importance of the microenvironment not only for CLL, and the value to test human primary material for drug responses, makes the paper timely and important.

The work therefore conceptually advances the field, and generates new data that is useful for researchers and clinicians. The key findings - trisomy 12 as an amplifier to environmental stimuli, targetable by BRD inhibition; IL4/TLR/IFNG signaling as potential resistance mechanism to certain treatments - are interesting, although not always fully convincing or highly significant. Along this line I have several comments to the content of this - in summary nice! - manuscript:

Major points:
• POT1 in not discussed in the manuscript although it seems to be the single significant mutational characteristics of M-CLL cluster 3 with fast progression. A mechanistic link also in light of the RNA-seq data to environmental stimuli (and drug responses) should be investigated at least on the bioinformatic level and/or discussed.
• Environment stimuli, drugs, and drug concentration - how are they selected? Also, 2 different concentrations are described in the table and a detailed description of viability measurements in addition to citation of published literature should be given
• Clustering of M-CLL cluster 3 and 4: Is this possibly related to the "intermediate CLL" group described in https://www.nature.com/articles/ng.2443 and https://www.nature.com/articles/ncomms11938 ? As DNA methylation data is available this could be tested (or otherwise)
• Fig1D/E/F relating to supplementary figure 2A/B: many negative correlations, analysis and interpretation would be nice, do they make sense, counter each other, or even amplify? This would be interesting in terms of potential additive effects coming from manifold microenvironmental stimuli in vivo.
• 279 fdr is high at 10%, how is it justified and how is the association with a lower FDR?
• Fig 2C cluster 3 what is the significances vs cluster 1/2? Is it significantly different in addition to C4 or in the same group as C1/2?
• Figure 3/4: molecular mechanisms of trisomy 12 is in my opinion the weakest part in the paper. Increased BCR signaling and other pathways in tri12 is known (e.g. https://doi.org/10.3324/haematol.2018.190132) and was correlated to DNA methylation data (although at a array-level that misses many distal regulatory elements). I have a hard time to believe that a general BRD inhibitor is specifically targeting a SPIB program. i) the pathways seem to be cherry picked to construct the story: several pathways that were extra run in 4D are in the hallmark set used for 4C (mtorc1, notch signaling) but not seen in gsea (or are they but not shown?). What is the normalized enrichment score and statistical testing? ii) SPIB targets - as SPIB is targeting thousands of sites in B cells, B cell pathways will likely be enriched simply due to the fact that it is a B cell malignancy, so I'd like to see the rank of the TLR/BCR/TGF enrichment based on all KEGG/REACTOME pathways. iii) concerning the TF enrichment, is the ATAC-seq data normalized for the third copy of chrom12, and if not yet but when done: how does this influence the diff peaks and discovered motifs? iv) the limitation of motif enrichment should be stated as several of the indicated TFs have very similar motifs. The analysis is just a hint for actual binding, which is an additional downside to the fact that a cell line and no primary CLL SPIB ChIP-seq data is used which can have quite a different SPIB DNA binding profile. v) ATAC-seq in ibet-treated samples: are these treated samples tri12 or non-tri12 samples? Even if the TF activity changes - do the changes occur at similar target genes or are some diff accessible peaks dominating the change in TF activity, meaning is it a general reduction of open chromatin sites of SPIB target genes, or are there specific peaks appearing/disappearing? Along this line, is SPIB expression (also PU.1 expression) different in the sample groups? l341-343 reads confusing, the TFs don't show higher accessibility but their putative binding sites I assume is the intention?
• IL4 enrichment in LN CLL cells also not really new (DOI: 10.1182/blood-2015-11-682906), also IFNG signaling as CLL supportive does not appear completely new to me (https://doi.org/10.4049/jimmunol.2000478), can the authors elaborate and put the significance of these findings in context?

Minor
• The DNA methylation/epigenomic information is not really analyzed to the extent the abstract / Fig 1A suggests, although it is available.
• Fig2C it could be noted in the text that no difference in OS is visible

• Figure 2D legend scale response coloring blue/red?
• l 248 reads slightly misleading as on the first read it seems to refer to cluster 3 not 4.
• Fig3: not well described. What is "methylation cluster LP/IP/HP"? What are the other 2 stimuli besides the 3 displayed?

Reviewer #2:

The study submitted by Bruch & Giles and coworkers deals about integrating molecular profiling samples with microenvironmental interactions and drug responses in vitro, all in the same chronic lymphocytic leukemia samples. This is indeed a powerful stragety to investigate the link between genetic alterations, interaction with signals from the microenvironment and drug sensitivities. As such, this approach, although applied to CLL, would be interesting for a broad readership in the field of personalized medicine in cancer. In spite of the general interest of the topic, I have several concerns related to how the story is unfolding, coherence of different parts of the manuscript, data presentation and interpretation.

General issues
1. The same authors have recently published an interesting article in Nat Cancer. That article reports a multiomic approach that includes genetic, epigenetic, transcriptional and ex vivo drug responses in the context of clinical data of CLL patients. If I am not mistaken, the same cases have been analyzed in the present manuscript but adding new layers of information. Therefore, this manuscript seems to be a continuation of the previous one, but this is not properly recognized in the manuscript text, nor the new findings are put in the context of the previous findings. What is the role of the multiomic component associated with proliferative drive by Lu et al and the data reported in the present manuscript?
2. The text/interpretation and graphical evidence shown in main figures are not always aligned, and makes data interpretation cumbersome in some instances (see below some major issues)
3. The identification, in Fig 2, of 4 clusters (2 U-CLL and 2 M-CLL) is indeed interesting. I was expecting that the manuscript was going to deepen into the nature of these clusters, but from Figure 3 on, these new clusters mostly disappear, and the analysis seems to be focused more on specific genetic changes. This feels like a break in the flow of ideas that confuses the message of the manuscript.

Major issues
4. In Figure 1E you show the association among specific stimuli and drug responses. In Suppl fig2, however, you graphically show the entire correlation matrix, which is far more informative than the selection of most correlated pairs. Please omit fig 1E and replace it by supl 2. Please also mark which ones of the correlations are statistically significant.
5. In lines 221-225 you describe the findings of suppl. Fig 3, but the text could be rephased to improve clarity. Please describe these findings 1) interpreting CLL as a whole and 2) interpreting differences between M-CLL and U-CLL.
6. Please correct me if I am wrong, but findings described in lines 232-237 do not seem to be coupled with the graphical evidence shown in main figure 2A. Which is are the values shown in this figure 2A (ranging from -3 to 3)? viability? Z-scores? The interpretation of the text can be followed only through the suppl. figure 4, but not through the main figure. For instance, you mention that C1/2 show stronger responses to IL4 and TLR7-9 agonists (Resiquimod and CpG ODN), sorry but I cannot see that in the main figure. I can neither see that C2 shows stronger responses to IL1beta and anti-IgM. In contrast, I clearly see that C1/2 have stronger responses to SDF-1alpha and BAFF. How is it possible that panel 2A and suppl fig 4 lead to different interpretations? Are you capturing in suppl fig 3 very subtle differences that not visually reflected in panel 2A? Perhaps I am missing something here, but this is confusing.
7. In lines 238-243 you link the 4 clusters to LDT. I am not sure whether the minimal (but significant) difference between C3 and C4 justifies the distinct clinical behavior. In a recent article from the same team (Nat Cancer 2021), part of the data of the current study are mined and they identify a signature related proliferative capacity (and linked to DNA hypomethylation, LDT and clinical behavior). Can you put the data of the present study also in the context of your previous report?
8. Fig 2D is a confusing way of presenting the link between clusters and genetic changes. Instead of enrichments, please provide the % of each genetic change in each cluster. C3 shows a very small enrichment in mutSF3B1, is seems to be significant, but which is the magnitude of the effect? Again, proportion of cases with a given mutation in each cluster would be a far more straight-forward way of showing/interpreting the data.
9. Lines 264-269: the pathways identified as differentially expressed in C3 vs C4 provide a link to your previous article in Nat Cancer. In line with comment 7, you may link/interpret the new data in the context of your previous study.
10. I have several remarks related to figure 3A: A) panel 2A clearly shows that U-CLLs (clusters 1-2) show increased responses to SDF-1alpha and BAFF. However, in this figure, it seems that IGHV status is unrelated to these stimulations. B) which gene alterations were used in this analysis? it seems that only copy number changes are related to these stimuli. Is there a reason why there is an enrichment in copy number changes? Some of them are actually rare events present in a low % of CLLs. C) copy number changes, as compared to gene mutations, do not have a clear target gene/pathway, and therefore, finding that almost only copy number changes are significant, is suggesting that genetic complexity rather than specific changes may be relevant. D) in line with this comment, could you do this analysis with genetic complexity (number of driver alterations, number of CNA..)?
11. In fig 3B, I do not understand what you mean with DNA methylation as co-variate, global DNA methylation levels, methylation

of specific genes, promoters?
12. With regard to tri12, in line 306 you mention that you performed differential expression and GSEA in positive vs negative cases. Did you perform this analysis separately for IGHV mutational status? This is unclear in the results text/figures. Going through the methods, it seems that you used 4 IGHV-M cases (clusters 3 and 4), but this has to be clarified in the results. Moreover, comparing 2 vs 2 cases seems indeed insufficient for a discovery series. In the case of the series by Rendeiro, this study analyzes 88 samples from 55 patients, and therefore, considering the sample size of your comparison (43 vs 9), I assume that you took tri12 cases vs WT regardless of their IGHV status. IGHV will be then a strong counfounding factor, as IGHV-M and -U show clearly different accessibility profiles and tri12 is actually enriched in IGHV-U cases. Additionally, I am aware of at least another series of CLL patients with available ATACseq data (Beekman et al., Nat Med 2018). It could potentially be also used for validation.
13. The potential role of SpiB in tri12 cases is interesting. However, the ChIPseq data are derived from DLBCL cell lines which have two important problems in your study: a) DLBCL has a clearly different biology than CLL, and b) cell lines and primary cases have different chromatin profiles. I am not sure how useful this dataset is in the context of your study. This limitation should be at least commented.

Minor issues
14. You sometimes refer to genetic alterations as mutations. In general, SVs are not considered mutations, and this term is used more for gene-specific mutations. This is a bit misleading (e.g. Fig 3A bottom). Please use genetic alterations as a general term and mutations when referring to specific genes.
15. The overall interpretation of the second part of the results section (lines 271-273) seems uncomplete and only biased towards the finding in M-CLLs.

Reviewer #3:

In this manuscript by Bruch et al. the authors investigate the impact of several microenvironmental stimuli on various drugs in a model of chronic lymphocytic leukemia (CLL). Using this system the authors identify 4 subgroups of CLL which respond differently to the chosen drugs and microenvironmental cues. The authors identify trisomy 12 as an important factor determining outcome dependent on microenvironmental signals and state that interleukin 4 and toll like receptor stimulation may contribute to resistance mechanisms in CLL. The manuscript is well written, informative and of interest, but it lacks significant validation in other, more biologically relevant model systems. For instance, the importance of adhesion to cells of the microenvironment (rather than mere exposure to soluble cytokines) or migration to microenvironmental components are completely disregarded.
Other concerns are:
1. How long were the cells cultured? Did the authors check that at the time of the experiments the cells still are phenotypically and functionally what they were at the beginning?
2. How was the concentration of the microenvironmental stimuli determined?
3. At least one validating/more mechanistic experiment should be performed to test the subroups in biologically meaningful ways. Do these subgrops contain more or less leukemic stem cells?
4. Figure 2C: In this reviewer's mind it is not sufficiently proven, that the differences in subgroups is due to the microenvironment.
5. Does group C3 consist of a group with genetic lesions previously known to be associated with poor response?
6. Suppl. Figure 5B: Why is there no difference in survival between C3 and C4?

We thank the reviewers for their time and effort spent reviewing our work, for their positive and encouraging overall assessment, and for their constructive suggestions to provide additional evidence and clarify our presentation. As the reviewers suggested, our main focus during revision has been on the following two main findings:

A) The characterisation and validation of the microenvironmental clusters described in Figure 2.
B) The effect of trisomy 12 in enhancing microenvironmental signalling and the downstream mechanism involving Spi-B as well as its potential targetability using BET inhibition.

Further, we have adapted their suggestions on figure design and display. We have particularly updated Figures 2 and 4 and added multiple Appendix figures with the aim of improving the overall clarity of our manuscript.
Additionally, we have updated the method descriptions, especially regarding the drug-stimulus-response profiling. We have added a step-by-step method section regarding the combinatorial drug-stimulus perturbation assay to improve the reproducibility. For the analyses we supply reproducible scripts as well as an online interactive app to explore the data.

For the convenience of the reviewers, we have added our answers in blue.
In cases where or answer directly connects to the manuscript, we have added a line annotation with the corresponding manuscript text in grey.

Reviewer #1:

Summary:
The manuscript of Bruch/Giles et al. describes an approach that tests microenvironmental stimuli on CLL viability and drug responses in 192 genetically characterized patient samples ex vivo. Additional available data of the cohort is used to connect genotype to drug responses/environmental stimuli. The authors show that they can classify CLL patients based on environmental stimuli responses, that the subgroups show distinct clinical characteristics, and that trisomy 12 seems to be a driver to response to such stimuli. Further Trisomy 12 is linked to a specific chromatin signature that might be targetable by BRD inhibition. As another key finding, the authors report in detail IL4 and TLR activity as the strongest stimuli, and IFN signaling as a new stimulus, which mediate potential treatment resistance. The data should facilitate understanding interactions between drugs and the microenvironment.

General remarks:
The paper is well written, and all data is available for interactive exploration. Although the ex vivo testing excludes the effect of drugs on the microenvironment cells, the idea to systematically test interactions of such environmental stimuli with drugs is very appealing. In light of the importance of the microenvironment not only for CLL, and the value to test human primary material for drug responses, makes the paper timely and important.

The work therefore conceptually advances the field, and generates new data that is useful for researchers and clinicians. The key findings - trisomy 12 as an amplifier to environmental stimuli, targetable by BRD inhibition; IL4/TLR/IFNG signaling as potential resistance mechanism to certain treatments - are interesting, although not always fully convincing or highly significant. Along this line I have several comments to the content of this - in summary nice! - manuscript:

Major points:

• POT1 is not discussed in the manuscript although it seems to be the single significant mutational characteristics of M-CLL cluster 3 with fast progression. A mechanistic link also in light of the RNA-seq data to environmental stimuli (and drug responses) should be investigated at least on the bioinformatic level and/or discussed.

POT1 mutations were significantly enriched in cluster 3 (3 out of 37 patient samples for whom DNA-sequencing data was available).The overall frequency of POT1 mutations in CLL was reported to be 3.5% (Ramsay *et al*, 2013) and this is lower than in cluster 3 of our cohort (8.1%) . POT1-mutated CLL cells have been reported to harbour numerous telomeric and chromosomal abnormalities that suggest that POT1 mutations favour the acquisition of the malignant features of CLL cells (Ramsay *et al*, 2013). This might contribute to the worse survival of cluster three patients but due its comparably low frequency it does not fully explain the observed clinical phenotype of this subgroup. We have updated Fig. 2D to more clearly represent the abundance of genetic alterations in the subgroups. We briefly discuss this aspect in the manuscript.

Line 183: POT1 mutations were enriched in C3, which may contribute to the worse survival of C3 patients.

Line 192: Notably, the observed difference in progression dynamics in vivo was not explained solely by the known prognostic markers IGHV status, trisomy 12, TP53, DNA methylation profiles, and POT1. A multivariate Cox proportional hazards model accounting for these features showed an independent prognostic value of the cluster assignment between C3 and C4 (p=0.03, Appendix Tab. 5).

• Environment stimuli, drugs, and drug concentration - how are they selected? Also, 2 different concentrations are described in the table and a detailed description of viability measurements in addition to citation of published literature should be given

Cell viability was measured after 48 hours of incubation with or without drugs and stimuli. The viability readout was performed using CellTiterGlo (Promega) per manufacturer's instructions. The drugs were selected based on the results from our previous study (Dietrich et al. 2018) as follows. We selected drugs that produced diverse patterns of responses across patient samples, and that covered targets important to the biology of CLL. For each drug, two concentrations were chosen to induce by itself (1) weak, sublethal effects and (2) very noticeable, toxic effects. With this experimental design, we anticipated to be able to observe both synergistic toxic effects and protective effects when used in combination with microenvironmental stimuli (Reagents and Tools table 1).

The stimuli were chosen based on a literature review of small scale *in vitro* studies of microenvironmental factors in CLL. Our choice(s) of concentrations also followed this literature and in some cases were refined by our own preliminary titration experiments (Reagents and Tools Table 2).

We have added this information to the main text. Following the editor's suggestion, we also added a step-by-step description of the protocol for the combinatorial drug-stimuli perturbation assay in the methods section and moved the tables describing concentrations and ordering information for drugs and stimuli from the Appendix to the Methods section.

Line 107: Relative viability was measured as luminescence-based ATP count (i.e. Celltiter Glo, Promega®) after 48h of drug-stimulus perturbation divided by the median of the DMSO controls. In subsequent analyses we used the natural logarithm of this value, so that

negative and positive values indicate pro- and antiapoptotic effects, respectively, and values close to 0 correspond to no effect.

Line 473 (Methods): Mononuclear cells were isolated from peripheral blood of CLL patients by a Ficoll gradient and cryopreserved as previously described(Dietrich et al, 2018). Cells were incubated for 48 hours with or without drugs and stimuli. The viability was measured by a luminescent ATP readout (CellTiter-Glo, Promega ®). The detailed methodology is described below. (Followed by detailed step-by-step protocol)

• Clustering of M-CLL cluster 3 and 4: Is this possibly related to the "intermediate CLL" group described in https://www.nature.com/articles/ng.2443 and https://www.nature.com/articles/ncomms11938 ? As DNA methylation data is available this could be tested (or otherwise)

To address this question, we added Appendix Fig. 6, which shows the DNA methylation profiles of all four clusters, and we added the DNA methylation status annotation to Fig. 2A. The majority of patient samples in Cluster 3 (C3) had a *high programmed* methylation profile. This was in contrast to the clinically indolent Cluster 4 (C4), which was enriched in *intermediate* and *low programmed* profiles. To check whether this difference explained the difference in clinical outcome observed between C3 and C4, we added the three DNA methylation groups as a covariate in the multivariate survival analysis of TTT (Appendix Table 3). In this analysis, the difference between C3 and C4 remained significant (p-value: 0.02, HR 0.35) even with IGHV status, trisomy 12, TP53 mutations, and DNA methylation groups as further covariates. This indicates that the observed clinical and biological phenotype of Cluster 3 is more than a mere consequence of DNA methylation group.

Line 187: In addition to genetic features, we also investigated the epigenetic profiles of the clusters. We observed that C1 and C2 were enriched for samples with low programmed (LP) DNA methylation profiles, whilst C3 and C4 contained many samples with high programmed (HP) DNA methylation profiles (Appendix Fig. 6) (Oakes et al, 2016).

Line 192: Notably, the observed difference in progression dynamics in vivo was not explained solely by the known prognostic markers IGHV status, trisomy 12, TP53, DNA methylation profiles, and POT1. A multivariate Cox proportional hazards model accounting for these features showed an independent prognostic value of the cluster assignment between C3 and C4 (p=0.03, Appendix Tab. 5).

• Fig1D/E/F relating to supplementary figure 2A/B: many negative correlations, analysis and interpretation would be nice, do they make sense, counter each other, or even amplify? This would be interesting in terms of potential additive effects coming from manifold microenvironmental stimuli in vivo.
CLL Drug-microenvironment interactions MSB First Revision

We have replaced the previous Fig. 1 D-F with the full correlation matrices to more clearly show all correlations between drugs and stimuli. Significant correlations (i.e. BH-transformed p-values<0.05) are labelled with the Pearson correlation coefficient.

With respect to the negative correlations that the reviewer mentions, the majority of these were between TGF-β1 and other stimuli. This makes sense due to the fact that TGF-β1 stimulation leads to a decreased viability in many patient samples, thus meaning it negatively correlates with the stimuli that induce an increase in viability.

Line 118: To assess the heterogeneity of the response patterns, we calculated Pearson correlation coefficients for each pair of drugs and each pair of stimuli. For the drugs, high correlation coefficients (R>0.75) were associated with identical target pathways. For example, drugs targeting the BCR pathway (ibrutinib, idelalisib, PRT062607 and selumetinib) were highly correlated, indicating that our data sensitively and specifically reflect inter-individual differences in pathway dependencies (Fig. 1D) (Dietrich et al, 2018).

In contrast, microenvironmental stimuli showed lower correlations, even where stimuli targeted similar pathways (Fig. 1E). For example, lower correlations were observed between different stimuli of the JAK-STAT and NfκB pathways, indicating a low degree of redundancy between stimuli. High correlations (R>0.75) were only seen with the TLR stimuli resiquimod (TLR7/8) and CpG ODN (TLR9) as well as IL4 and IL4 + soluble CD40L (sCD40L), which target near-identical receptors and downstream targets.

• 279 fdr is high at 10%, how is it justified and how is the association with a lower FDR?

The 10% threshold was selected as a trade-off between statistical power (false negatives) and false positives. We chose this threshold in the knowledge that this figure represents an exploratory analysis in which promising hits were later investigated in more detail and with additional experiments, such as the analysis of trisomy 12 in Fig. 4.

Our overall results do not change when a more stringent cut-off of 5% is applied (see below). In particular, the extent to which genetic alterations influence stimulus response as well as the effect of IGHV mutation status and trisomy 12, which we highlight in the text, remains the same.

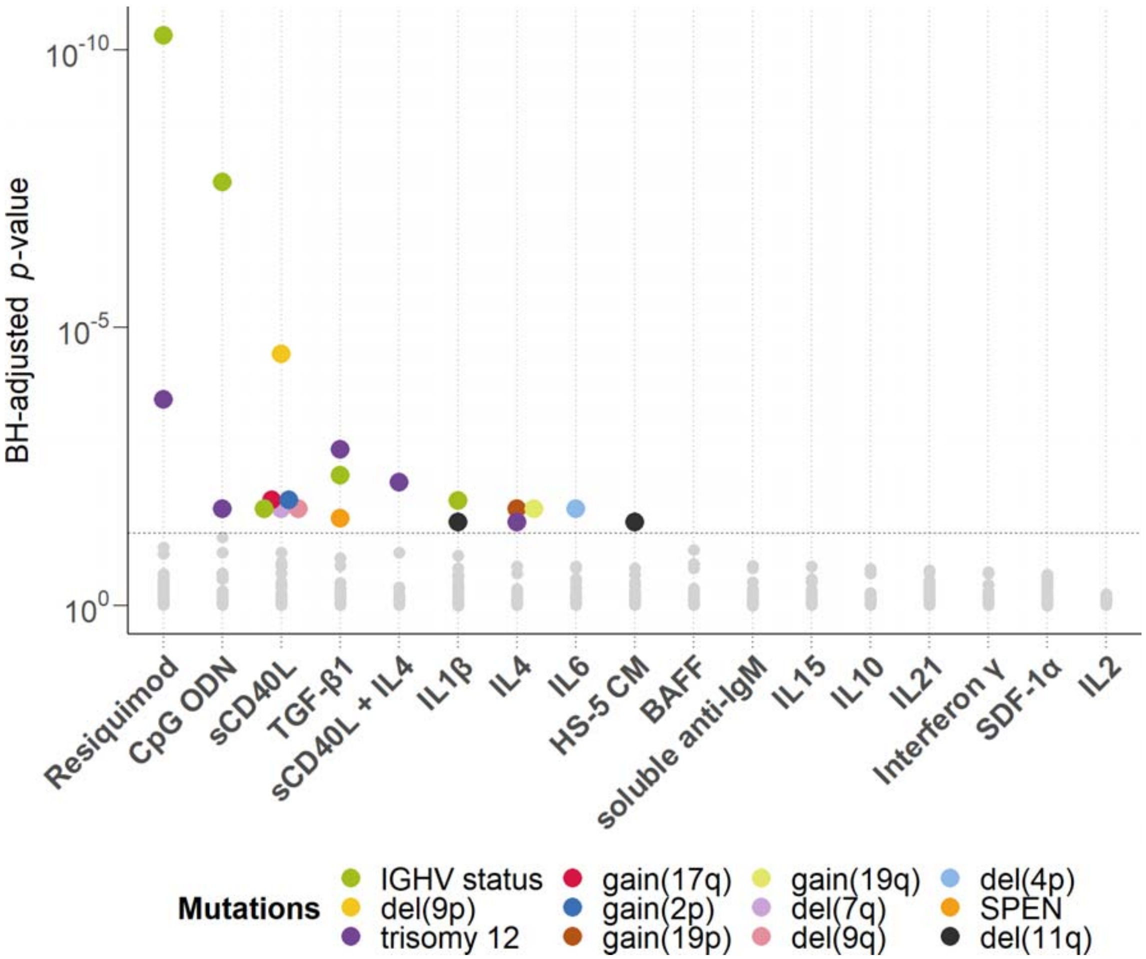

Revision Figure 1: Associations between genetic features (gene mutations and structural aberrations) and stimulus response. x-axis shows stimuli, y-axis shows p-values from Student's t-test (two-sided, with equal variance). Each dot represents a genetic feature-stimulus association. Tests with p-values smaller than the threshold corresponding to a false discovery rate (FDR) of 5% (method of Benjamini and Hochberg) are indicated by coloured circles, where the colours represent the genetic features.

• Fig 2C cluster 3 what is the significances vs cluster 1/2? Is it significantly different in addition to C4 or in the same group as C1/2?
To more clearly show the association between Cluster assignment and survival we added a univariate survival analysis in Appendix Tab. 3. Here, we observed that Cluster 3 has a significantly shorter TTT than C4 and a significantly longer TTT than Cluster 2. There is no significant difference between TTT of the patients in Cluster 3 and Cluster 1.

When combining the predominantly IGHV-U Clusters 1 and 2 and comparing these to Cluster 3 we do not observe a significant difference (Appendix Tab. 4).

• Figure 3/4: molecular mechanisms of trisomy 12 is in my opinion the weakest part in the paper.
We thank the reviewer for highlighting their concerns about this figure and for their helpful suggestions on how to address these. Individual points are discussed below. In summary, we have:

- Removed the GSEA analysis comparing trisomy 12 and non-trisomy 12 CLL as this lacked novelty
- Validated our finding that Spi-B and PU.1 are upregulated in trisomy 12 CLL by confirming that this result is independent of the third copy of chromosome 12 and by validating in a second external dataset from Beekman et al. (2018)
- Visualised the locations of Spi-B binding sites that show differential accessibility in trisomy 12 CLL, and linked these to target genes
- Complemented our analysis of Spi-B targets in lymphoma cell lines with data from primary CLL ATACseq data, to support our hypothesis that microenvironmental signalling pathways are enriched amongst Spi-B target genes

Increased BCR signaling and other pathways in tri12 is known (e.g. https://doi.org/10.3324/haematol.2018.190132) and was correlated to DNA methylation data (although at a array-level that misses many distal regulatory elements).
We agree with the reviewer and have removed the previous Fig. 4C + D, which showed a GSEA analysis of trisomy 12 versus non-trisomy 12 samples. Instead, we refer to previous work in our lab (Dietrich *et al*, 2018; Herbst *et al*, 2022a) that showed that transcripts and proteins involved in BCR signalling are more abundant in trisomy 12.

Line 248: Previous work has indicated that trisomy 12 CLLs show higher transcript and protein abundance of microenvironmental pathways including BCR (Herbst et al, 2022b) and chemokine signalling genes (Dietrich et al, 2018).

I have a hard time to believe that a general BRD inhibitor is specifically targeting a SPIB program.
We have adjusted the text to indicate that IBET-762 treatment leads to downregulation of many transcription factors, among which includes Spi-B.

Line 301: The inferred TF activity was decreased upon IBET-762 treatment in a broad range of TFs, including all nine TFs with higher inferred activity in trisomy 12 CLL (Fig. 4F).

i) the pathways seem to be cherry picked to construct the story: several pathways that were extra run in 4D are in the hallmark set used for 4C (mtorc1, notch signaling) but not seen in gsea (or are they but not shown?). What is the normalized enrichment score and statistical testing?
We have removed this GSEA and the associated figure panels 4C and C4. Instead, we refer to previous work in our lab (Dietrich *et al*, 2018; Herbst *et al*, 2022a) that showed that transcripts and proteins involved in BCR signalling are more abundant in trisomy 12.

Line 248: Previous work has indicated that trisomy 12 CLLs show higher transcript and protein abundance of microenvironmental pathways including BCR (Herbst et al, 2022b) and chemokine signalling genes (Dietrich et al, 2018).

ii) SPIB targets - as SPIB is targeting thousands of sites in B cells, B cell pathways will likely be enriched simply due to the fact that it is a B cell malignancy, so I'd like to see the rank of the TLR/BCR/TGF enrichment based on all KEGG/REACTOME pathways.
We thank the reviewer for highlighting this. We originally ran this over-representation analysis on immune pathways, in line with our hypothesis that Spi-B coordinates transcriptional regulation of microenvironmental signalling pathways. We have now repeated this analysis on all KEGG and REACTOME pathways, which generates many significant results including immune signalling pathways. To address the reviewer's

suggestion, the current analysis now includes the original immune signalling pathways plus control pathways (Oxidative Phosphorylation, P53 signalling pathway, DNA replication, Cell Cycle). None of these controls are significant indicating that immune signalling pathways specifically are enriched.

Line 285: We ran over-representation tests to investigate enrichment of immune and control pathways amongst these gene targets. We found that JAK-STAT signalling was enriched amongst these genes (Appendix Tab. 6).

iii) concerning the TF enrichment, is the ATAC-seq data normalized for the third copy of chrom12, and if not yet but when done: how does this influence the diff peaks and discovered motifs?
To confirm that the additional copy of chromosome 12 was not influencing the result, we reran both diffTF comparisons (smaller and larger datasets) by removing peaks on chromosome 12 from the input peak files. The set of transcription factors showing differential binding site accessibility was not significantly affected, and Spi-B and PU.1 remained the top hits (Appendix Fig. 16).

Line 271: To check whether the results were affected by the additional reads on chromosome 12, we repeated these analyses without including data from chromosome 12 (Appendix Fig. 16) and Spi-B and PU.1 remained the top hits.

iv) the limitation of motif enrichment should be stated as several of the indicated TFs have very similar motifs. The analysis is just a hint for actual binding, which is an additional downside to the fact that a cell line and no primary CLL SPIB ChIP-seq data is used which can have quite a different SPIB DNA binding profile.
We have adjusted the wording in the text to more accurately reflect the limitation of using motifs

Line 258: Both are hematopoietic regulators which exhibit functional redundancy (Garrett-Sinha et al, 2001). They also have similar binding motifs which makes it difficult to distinguish whether either or both are upregulated in trisomy 12 based on this motif-based method of inferring activity .

In addition, we recognise the limitation of using lymphoma cell line data to investigate Spi-B gene targets. However, it is also prohibitively difficult (beyond our means) to generate ChIP-Seq data for primary CLL samples, and no CLL cell line exists. Instead, we tried to perform further validatory work using primary CLL ATACseq data.

More specifically, we approximately identified target genes of the Spi-B transcription factor from this primary ATACseq data. We subset the Spi-B motif locations based on whether they showed differential accessibility between trisomy 12 and non-trisomy 12 and then proceeded to treat these motif locations in a similar way to ChIPseq peaks and annotated each motif with its nearest gene. We ran over-representation tests to investigate enrichment of immune pathways amongst these gene targets. Thus, the manuscript now includes two analyses of Spi-B gene targets, based on both the primary CLL ATACseq data and the ChIPseq data from lymphoma cell lines (Appendix Tab. 6 & 7).

Line 284: We next approximated Spi-B target genes in trisomy 12 CLL by annotating each of these motif locations with the nearest gene. We ran over-representation tests to investigate enrichment of immune and control pathways amongst these gene targets. We found that JAK-STAT signalling was enriched amongst these genes (Appendix Tab. 6).

v) ATAC-seq in ibet-treated samples: are these treated samples tri12 or non-tri12 samples?
The ATACseq of IBET-762 treated CLL samples contain two trisomy 12 and two non-trisomy 12 samples.

Even if the TF activity changes - do the changes occur at similar target genes or are some diff accessible peaks dominating the change in TF activity, meaning is it a general reduction of open chromatin sites of SPIB target genes, or are there specific peaks appearing/disappearing? Along this line, is SPIB expression (also PU.1 expression) different in the sample groups?
CLL Drug-microenvironment interactions MSB First Revision
In the original manuscript we focused on global changes to Spi-B binding sites and we thank the reviewer for highlighting the importance of investigating specific changes. We thus investigated whether the changes in inferred TF activity calculated by diffTF were due to global changes across many motifs, or if some differentially accessible motifs were dominating the result.

To visualise the effects of Spi-B we plotted the genomic locations of all Spi-B binding sites that showed an absolute log2FC >1, and an associated adjusted p-value < 0.1 (Appendix Fig. 17). This analysis indicated that Spi-B motifs at certain sites show strong differential accessibility between trisomy 12 and non-trisomy 12 and that these are distributed across the genome. We next annotated each of these binding sites with their nearest gene and ran over-representation tests to investigate enrichment of immune and control pathways amongst these target genes. We found that JAK-STAT signalling is enriched amongst these target genes (Appendix Tab. 6) The manuscript now includes this analysis in both the text and Appendix figures (Appendix Fig. 17 and Appendix Tab. 6).

Line 281: To investigate this, we first visualised the genomic locations of all Spi-B motifs that showed differential inferred activity in trisomy 12 samples (Appendix Fig. 17). This analysis indicated that Spi-B binding motifs showing differential accessibility in trisomy 12 CLL are distributed throughout the genome. We next approximated Spi-B target genes in trisomy 12 CLL by annotating each of these motif locations with the nearest gene. We ran over-representation tests to investigate enrichment of immune and control pathways amongst these gene targets. We found that JAK-STAT signalling was enriched amongst these genes (Appendix Tab. 6).

We further investigated the RNA expression and protein abundance of Spi-B as well as PU.1 and SPI1, the gene encoding for PU.1 in our recently published dataset (Herbst *et al*, 2022b). Spi-B was not differentially expressed in trisomy 12 CLL, and was not detected within our proteomics dataset. PU.1 (SPI1) showed higher gene expression (p=0.0032) and higher protein abundance (p=0.00037) in trisomy 12 CLL. Gene dosage effects might therefore influence the higher activity of PU.1 in trisomy 12 CLL.

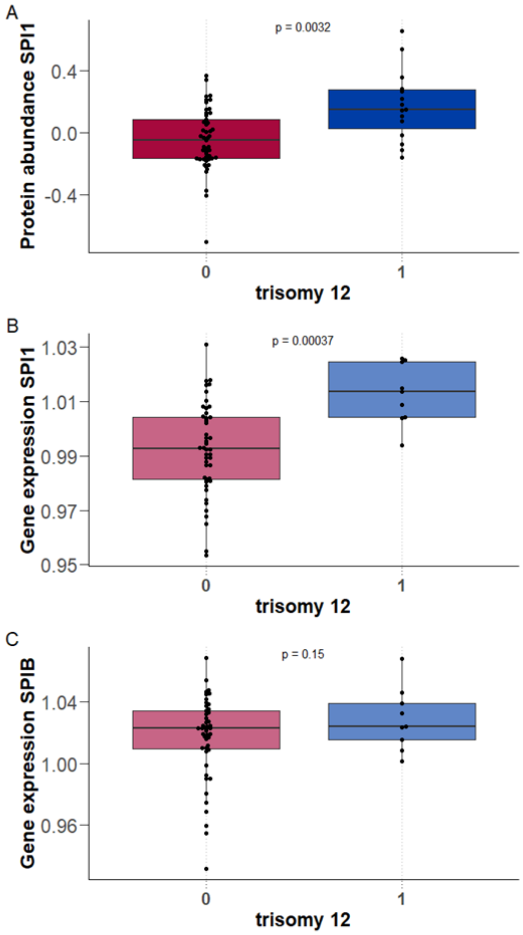

Revision Figure 2: SPI1/PU.1 gene expression and protein abundance in CLL samples with and without trisomy 12. Figures adapted from (Herbst *et al*, 2022b) and available as online application under: dietrichlab.de/CLL_Proteomics/

l341-343 reads confusing, the TFs don't show higher accessibility but their putative binding sites I assume is the intention?

We thank the reviewer for highlighting this ambiguous wording, we were indeed referring to the putative binding sites. We have now updated the text to reflect this.

Line 253: Building on these studies of the trisomy 12 CLL transcriptome and proteome, we investigated the impact on the epigenome. We used chromosome accessibility data from ATAC sequencing to infer transcription factor (TF) activity based on motif accessibility. We quantified inferred TF activity in trisomy 12 and non-trisomy 12 CLL PBMC samples (n=4). 92 TFs demonstrated higher inferred activity (p<0.05) in the trisomy 12 samples (Appendix Fig. 14), reflecting a specific signalling signature in trisomy 12 CLL. Spi-B and PU.1 were the top hits.

• IL4 enrichment in LN CLL cells also not really new (DOI: 10.1182/blood-2015-11-682906), also IFNG signaling as CLL supportive does not appear completely new to me

(https://doi.org/10.4049/jimmunol.2000478), can the authors elaborate and put the significance of these findings in context?

We thank the reviewer for highlighting these studies, which we had previously overlooked. We are pleased that these results are in line with our own observations and support the quality of our data and analysis. More specifically:

Aguilar-Hernandez et al. (Aguilar-Hernandez *et al*, 2016) showed that CLL cells extracted from lymph nodes (Herishanu *et al*, 2011) revealed a significant enrichment of expression of IL4 target genes (Ruiz-Lafuente *et al*, 2014) compared to CLL cells isolated from peripheral blood. This analysis employed 24 patient samples and highlighted the relative signalling activity in different anatomical compartments. Their results align with our finding that IL4 signalling is higher in microenvironmental niches.

However, we would like to point out that the analysis by Aguilar-Hernandez et al. (based on data from Herishanu et al.) was potentially influenced by the extraction of CLL cells from lymph node biopsies. The method cited by the authors (Herishanu *et al*, 2011) specifies that mononuclear cells were isolated by centrifugation over lymphocyte separation medium, followed by CD19+ selection. The exact method used to obtain a single cell solution from the lymph node biopsies is not specified. This leaves the concern that some of the observed signalling patterns could have been modulated by the extraction process.

In contrast, we investigated the content of phosphorylated STAT6 using immunohistochemistry in primary lymph node biopsies as a proxy for IL4 signalling activity. Thereby, we avoided potential biases on pathway activity introduced by isolating viable cells from lymph nodes. On the other hand, our analysis is contingent on the fixative process and the indirect measurement of IL4 activity using pSTAT6.

Further, our analysis differs from that performed by Herishanu et al. in that we compared microenvironmental signalling activity between CLL and tumour-free lymph nodes, rather than between anatomical compartments. Our large, clinically annotated cohort also enabled us to correlate treatment progression with the measured microenvironmental signalling activity. We found that increased signalling activity of IL4 was associated with shorter treatment free survival (Fig. 7G). This is a novel result and directly links microenvironmental activity to faster disease progression.

Line 435: Further, we observed increased IL4 activity within CLL-infiltrated lymph nodes compared to tumour-free lymph nodes, which complements previous work demonstrating higher IL4 activity in the lymph node compared to peripheral blood (Aguilar-Hernandez et al, 2016). Increased IL4 signalling activity was associated with faster disease progression.

We also thank the reviewer for highlighting the study by Xia et al. (Xia *et al*, 2020), which investigated the effect of ibrutinib and IFNγ in a cohort of 11 CLL patient samples. The results of this study are consistent with our results and highlight the robustness of our scientific approach. We have updated the manuscript to cite this previously published result.

Line 333: The action of targeted therapies (including BCR inhibitors) was also reduced by the stimuli, most commonly by IL4 (Fig. 5D) and interferon-γ (IFNγ, Fig. 5E) in alignment with other studies on IL4 and IFNγ (Xia et al, 2020; Aguilar-Hernandez et al, 2016). IL4 and IFNγ reversed drug action in a common set of drugs, suggesting that both stimuli may act via a common mechanism.

Line 427: Building on work on IL4 and IFN induced drug resistance (Aguilar-Hernandez et al, 2016; Xia et al, 2020), our assay demonstrates the importance of both pathways in inducing resistance across a range of BCR inhibitors and chemotherapeutics.

Minor

• The DNA methylation/epigenomic information is not really analyzed to the extent the abstract / Fig 1A suggests, although it is available.

We have investigated the DNA methylation profiles both in regard to their impact on microenvironmental response (Fig. 3) and their role in the distinct clusters defined in Fig. 2. To make this more clear we have added Appendix Fig. 6 and included DNA methylation profiles in the multivariate survival analysis (Appendix Table 5).

Another layer of epigenetic information was obtained by ATAC Sequencing and is central to our analysis of the effects of trisomy 12 in the context of microenvironmental signalling (Fig. 4C&F).

• Fig2C it could be noted in the text that no difference in OS is visible
We have noted in the manuscript (Line XXX) that no difference was observed in overall survival between C3 and C4.

Due to the high median age of onset of CLL, the indolent nature of the disease, and the many effective treatment options, CLL is no longer a leading cause of death, and therefore prognostic differences only partly translate into overall survival. We chose Time to Next Treatment (TTT) as a biologically meaningful measure of disease outcomes, which is independent of the available treatment options. It reflects the biological aggressiveness of the disease which ultimately leads to a treatment indication, including anaemia, thrombocytopenia or massive lymph node enlargement.

Line 163: Overall survival was not detectably different between C3 and C4 (Appendix Fig. 4B).

• Figure 2D legend scale response coloring blue/red?
We have updated Fig. 2D. It now shows the absolute number of patient samples with each genetic feature and the total number of patient samples in each cluster for which the genetic information is available. We aim to provide a clearer indication of the level of depletion or enrichment of each given feature. To avoid confusion between Fig. 2A and Fig. 2D we intentionally did not use the blue/red colour scale here and instead use the colours associated with cluster assignment in Fig. 2A-D.

• l 248 reads slightly misleading as on the first read it seems to refer to cluster 3 not 4.
We have now updated the text to indicate more clearly that we are referring to C3, which shows faster disease progression than C4.

Line 163: This points towards a subset of mostly IGHV-M patients with more aggressive disease (C3), which is characterised by its differential response to microenvironmental stimuli.

• Fig3: not well described. What is "methylation cluster LP/IP/HP"? What are the other 2 stimuli besides the 3 displayed?

Distinct patient subgroups in CLL can be distinguished by whole genome DNA methylation data. These groups are differentiated into *low*, *intermediate* and *high programmed* DNA methylation profiles and impact clinical outcomes (Oakes *et al*, 2016).

Line 187: In addition to genetic features, we also investigated the epigenetic profiles of the clusters. We observed that C1 and C2 were enriched for samples with low programmed (LP) DNA methylation profiles, whilst C3 and C4 contained many samples with high programmed (HP) DNA methylation profiles (Appendix Fig. 6) (Oakes et al, 2016).

We have added the stimuli with significant coefficients assigned by the lasso model as Appendix Fig. 13. These are resiquimod and sCD40L+IL4.

Reviewer #2:

The study submitted by Bruch & Giles and coworkers deals about integrating molecular profiling samples with microenvironmental interactions and drug responses in vitro, all in the same chronic lymphocytic leukemia samples. This is indeed a powerful stragety to investigate the link between genetic alterations, interaction with signals from the microenvironment and drug sensitivities. As such, this approach, although applied to CLL, would be interesting for a broad readership in the field of personalized medicine in cancer. In spite of the general interest of the topic, I have several concerns related to **how the story is unfolding**, **coherence** of different parts of the manuscript, **data presentation** and **interpretation**.

General issues

1. The same authors have recently published an interesting article in Nat Cancer. That article reports a multiomic approach that includes genetic, epigenetic, transcriptional and ex vivo drug responses in the context of clinical data of CLL patients. If I am not mistaken, the same cases have been analyzed in the present manuscript but adding new layers of information. Therefore, this manuscript seems to be a continuation of the previous one, but this is not properly recognized in the manuscript text, nor the new findings are put in the context of the previous findings. What is the role of the multiomic component associated with proliferative drive by Lu et al and the data reported in the present manuscript?

Results: CLL Drug-microenvironment interactions MSB First Revision

Discussion: CLL Drug-microenvironment interactions MSB First Revision

In this publication (Lu *et al*, 2021), we described a previously unknown biological axis associated with disease progression in CLL. The discovery of this so-called CLL proliferative drive (CLL-PD) was based on observational multi-omics data and tumour drug response, but the study did not systematically investigate the role of microenvironmental stimuli.

Following the reviewer's very appropriate suggestion, we have now added an analysis that investigates the link between microenvironmental response and CLL-PD, summarised in Appendix Fig. 6. Panel A shows the proliferative drive of the patient samples stratified by the four clusters. C3 shows a significantly ($p=0.006$) higher proliferative drive compared to C4.

Furthermore, we looked for associations between CLL-PD and the response to each stimulus, using multivariate regression (Appendix Fig. 6B). CLL-PD in IGHV-M and U samples was associated with distinct pathways. In IGHV-M, CLL-PD correlated with responses to TLR stimuli (Appendix Fig. 6C&E). In IGHV-U, it correlated with NFkB and TGF-beta stimuli (sCD40L, IL-1β, soluble anti-IgM and TGF-β1, Appendix Fig. 6D&F).

In summary, in our previous work we found that CLL-PD was not associated with specific mutations but rather with their total number, and with expression profiles. In our new work, we observe CLL-PD associated with different responses to microenvironmental stimuli, and thus provide further biological characterisation of this novel biomarker.

Line 167: CLL Proliferative Drive (CLL-PD) is a recently discovered molecular biomarker profile that captures the proliferative capacity of CLL cells based on data from multiple omic layers (Lu et al, 2021). CLL-PD was significantly higher in C3 samples than in C4, and higher in C2 samples than in C1 (Appendix Fig. 5A). To understand these relationships, we looked for associations between CLL-PD and the response to each stimulus, using multivariate regression (Appendix Fig. 5B). CLL-PD was associated with distinct pathways in IGHV-M and

U disease. In IGHV-M, CLL-PD correlated with responses to TLR stimuli (Appendix Fig. 5C&E). In IGHV-U, it correlated with NFkB and TGF-beta stimuli (sCD40L, IL-1β, soluble anti-IgM and TGF-β1, Appendix Fig. 5D&F).

Line 422: Further, we link microenvironmental response phenotypes to the CLL Proliferative Drive (Lu et al, 2021), further characterising the biology of this newly proposed patient stratification method.

2. The text/interpretation and graphical evidence shown in main figures are not always aligned, and makes data interpretation cumbersome in some instances (see below some major issues)

We thank the reviewer for raising this point. We have revised the manuscript in multiple places to make it easier to follow, including in all places mentioned below by the reviewer. We describe the specific changes under the respective reviewer comments.

3. The identification, in Fig 2, of 4 clusters (2 U-CLL and 2 M-CLL) is indeed interesting. I was expecting that the manuscript was going to deepen into the nature of these clusters, but from Figure 3 on, these new clusters mostly disappear, and the analysis seems to be focused more on specific genetic changes. This feels like a break in the flow of ideas that confuses the message of the manuscript.

The reviewer is correct. While we agree that research papers ideally have a single "story" thread, in this case there are indeed two somewhat separate strands. This is in the nature of our results, and despite the drawbacks we feel that trying to press this into a single "story" would not be appropriate.

Instead, we chose to structure the presentation as follows: we follow a stepwise systems medicine approach, which describes the interplay of cell intrinsic genetic features and cell extrinsic signals from the microenvironment and how they cooperate and modulate anti-cancer drug responses. We started the analysis by describing similarities and differences of responses to microenvironmental stimuli. This analysis revealed four subgroups with distinct responses to microenvironmental stimuli which had clinically distinct progression dynamics. This is indeed a striking result, but we do not propose this stratification as a clinical tool, because it is based on a complex experimental readout, and it is not ripe for use as a biomarker or stratification principle in a clinical setting. However, the main intention of this analysis was to illustrate that variable responses to microenvironmental stimuli *ex vivo* reflect clinically important disease biology of CLL.

In the next step we aimed to understand if and how genetic features determine these microenvironmental response patterns. We did this by systematically comparing the genetic makeup of the subgroups (Fig. 2D) and regressing each genetic feature separately on each stimulus (Fig. 3A). Finally we systematically investigated how this interplay of genetic features and microenvironmental stimuli may modulate drug response (Fig. 5 & 6). We have now added a systematic comparison of drug response profiles across the subgroups defined by microenvironmental response (Appendix Fig. 9 & 10).

Major issues

4. In Figure 1E you show the association among specific stimuli and drug responses. In Suppl fig2, however, you graphically show the entire correlation matrix, which is far more informative than the selection of most correlated pairs. Please omit fig 1E and replace it by supl 2. Please also mark which ones of the correlations are statistically significant.

We thank you for this encouraging suggestion to present our data more comprehensively. Following this suggestion, we moved the full correlation matrices from the supplemental material to Fig. 1 D & E. To avoid overcrowding the figure, we limited the text-labels of correlation coefficients to those that were statistically significant values, i.e. those with BH adjusted p-values < 0.05 (BH adjustment performed separately for drug and stimulus correlations).

5. In lines 221-225 you describe the findings of suppl. Fig 3, but the text could be rephased to improve clarity. Please describe these findings 1) interpreting CLL as a whole and 2) interpreting differences between M-CLL and U-CLL.

In the passage mentioned by the reviewer we discuss the overall effects of microenvironmental stimuli on CLL cells. While we agree that in the case of TLR agonists, a distinct effect between M-CLL and U-CLL is present and visible here, we discuss this. We decided to build the manuscript in this way to enable readers with less background in CLL biology to follow our findings.

Line 131: Most stimuli increased CLL viability, underlining the supportive nature of the microenvironment. However, IL6 and tumour growth factor β (TGFβ) decreased viability (Appendix Fig. 2). The strongest responses were seen with IL4 and TLR7/8/9 agonists, highlighting the potency of these pathways in modulating CLL survival.

6. Please correct me if I am wrong, but findings described in lines 232-237 do not seem to be coupled with the graphical evidence shown in main figure 2A. Which is are the values shown in this figure 2A (ranging from -3 to 3)? viability? Z-scores? The interpretation of the text can be followed only through the suppl. figure 4, but not through the main figure.

For instance, you mention that C1/2 show stronger responses to IL4 and TLR7-9 agonists (Resiquimod and CpG ODN), sorry but I cannot see that in the main figure. I can neither see that C2 shows stronger responses to IL1beta and anti-IgM. In contrast, I clearly see that C1/2 have stronger responses to SDF-1alpha and BAFF. How is it possible that panel 2A and suppl fig 4 lead to different interpretations? Are you capturing in suppl fig 3 very subtle differences that not visually reflected in panel 2A? Perhaps I am missing something here, but this is confusing.

The viability measurements in Fig2A are modified z scores for optimal visualisation. These were calculated from the natural logarithm of the relative viability used in all other analyses. Essentially, these viability measurements were row-scaled using the median absolute deviation, and limits were applied to the scaling to ensure all values are between 3 and -3 to improve the readability.

In the previous version of this manuscript the row labels of Fig. 2A were incorrectly ordered due to an error in producing the figure. We sincerely apologise for this mistake and have corrected it in the current version. The interpretation of the figure now aligns with the figure itself.

Line 944: The data are shown on a robust z-score scale, i.e., the logarithm of the relative viability measurements were scaled by the median absolute deviation within each row. Limits were applied to scaling factor for optimal visualisation. Red indicates increased viability, blue indicates decreased viability. Samples are annotated for genetic features, sex and pre-treatment status.

7. In lines 238-243 you link the 4 clusters to LDT. I am not sure whether the minimal (but significant) difference between C3 and C4 justifies the distinct clinical behavior.

The observed difference in LDT represents one level of clinical annotation which highlights the different progression dynamics of Clusters 3 and 4. The shorter TTT for Cluster 3 shown in Fig. 2C leads to the same conclusion.

Fig. 2B shows LDT on a logarithmic scale. The median LDT of Cluster 3 is 2.3 years, while the median LDT of Cluster 4 is 4.19 years, meaning that the progression from 20 lymphocytes per nl of peripheral blood to 160/nl would take roughly 6.9 and 12.5 years respectively.

Line 152: In line with clinical observations (Baumann et al, 2021), the IGHV-U enriched C1 and C2 showed a shorter lymphocyte doubling time than the IGHV-M enriched C3 and C4. More strikingly, C3 showed a significantly shorter lymphocyte doubling time than C4, indicating a higher proliferative

In a recent article from the same team (Nat Cancer 2021), part of the data of the current study are mined and they identify a signature related proliferative capacity (and linked to DNA hypomethylation, LDT and clinical behavior). Can you put the data of the present study also in the context of your previous report?

In a recent publication (DOI: 10.1038/s43018-021-00216-6), we have described a previously unknown biological axis of progressiveness in CLL patients. This so-called CLL proliferative drive (CLL-PD) is based on multi-omics and drug response data for each of the patient samples in the cohort. In this manuscript, we also investigated the effect the relationship between TLR response and CLL-PD.

In the current manuscript, we have now added an appendix figure investigating the relationship between microenvironmental response more generally, and the previously described CLL-PD. We explain this in more detail above, under *General Issues.*

While our publication in Nature Cancer describes a previously unknown biological axis of progressiveness which can be used to stratify CLL patients, we here improve the general understanding of CLL microenvironmental response. This provides further possible biological explanation to the axis captured by CLL-PD. Further, we provide a community resource to expedite hypothesis generation and testing and suggest a methodology for broad interaction experiments to be applied in other cancer entities.

Line 167: CLL Proliferative Drive (CLL-PD) is a recently discovered molecular biomarker profile that captures the proliferative capacity of CLL cells based on data from multiple omic layers (Lu et al, 2021). CLL-PD was significantly higher in C3 samples than in C4, and higher in C2 samples than in C1 (Appendix Fig. 5A). To understand these relationships, we looked for associations between CLL-PD and the response to each stimulus, using multivariate regression (Appendix Fig. 5B). CLL-PD was associated with distinct pathways in IGHV-M and U disease. In IGHV-M, CLL-PD correlated with responses to TLR stimuli (Appendix Fig. 5C&E). In IGHV-U, it correlated with NFkB and TGF-beta stimuli (sCD40L, IL-1β, soluble anti-IgM and TGF-β1, Appendix Fig. 5D&F).

Line 422: Further, we link microenvironmental response phenotypes to the CLL Proliferative Drive (Lu et al, 2021), further characterising the biology of this newly proposed patient stratification method.

8. Fig 2D is a confusing way of presenting the link between clusters and genetic changes. Instead of enrichments, please provide the % of each genetic change in each cluster. C3 shows a very small enrichment in mutSF3B1, is seems to be significant, but which is the magnitude of the effect? Again, proportion of cases with a given mutation in each cluster would be a far more straight-forward way of showing/interpreting the data.

We have changed Fig. 2D according to the reviewer's suggestion. Most importantly, we have added an annotation of the number of samples with the given genetic feature and the total number of samples where information on this annotation is present.

9. Lines 264-269: the pathways identified as differentially expressed in C3 vs C4 provide a link to your previous article in Nat Cancer. In line with comment 7, you may link/interpret the new data in the context of your previous study.

As shown in the new Appendix Fig. 5, C3 and C4 show a significantly different CLL-PD. These clusters show differential activity of a similar set of pathways as are captured by CLL-PD, including mTOR, MYC and Oxidative Phosphorylation.

C3 and C4 also demonstrate differential intrinsic cytokine signalling activity and show distinct responses to the stimuli. This suggests microenvironmental signalling may be a determinant of CLL proliferative drive, providing further biological characterisation of this novel biomarker.

10. I have several remarks related to figure 3A:

A) panel 2A clearly shows that U-CLLs (clusters 1-2) show increased responses to SDF-1alpha and BAFF. However, in this figure, it seems that IGHV status is unrelated to these stimulations.

This misunderstanding arises from the incorrect labelling of the rows in Fig. 2A, which we have now fixed. SDF-1α and BAFF are not significantly influenced by IGHV mutation status when applying a 10% FDR cut-off after BH multiple testing adjustment.

B) which gene alterations were used in this analysis? It seems that only copy number changes are related to these stimuli. Is there a reason why there is an enrichment in copy number changes? Some of them are actually rare events present in a low % of CLLs.

We have included a table of all genetic features which were considered in the univariate analysis of Fig. 3A (Appendix Tab. 2). For this analysis, genetic features were considered which had a minimum of three positive cases. This left a high number of chromosomal aberrations, which explains their high frequency in Fig. 3A.

C) copy number changes, as compared to gene mutations, do not have a clear target gene/pathway, and therefore, finding that almost only copy number changes are significant, is suggesting that genetic complexity rather than specific changes may be relevant.

Chromosomal aberrations are known to play a dominant role in CLL disease biology and are prominently used for clinical stratification. (Döhner *et al*, 2000). Therefore, the strong influence on microenvironmental response is not surprising. While a complex karyotype is a clinically used marker, there is no clear consensus definition of genetic complexity in CLL.

D) in line with this comment, could you do this analysis with genetic complexity (number of driver alterations, number of CNA..)?

We thank the reviewer for highlighting this important point. We have investigated whether the presence of at least three chromosomal aberrations impacts the microenvironmental response. In line with the clinically used nomenclature, we termed this Complex Karyotype. We found that the presence of a so-called Complex Karyotype influences the response to TLR stimulation (resiquimod and CpG ODN) as well as sCD40L. We have added this to Fig. 3A.

11. In fig 3B, I do not understand what you mean with DNA methylation as co-variate, global DNA methylation levels, methylation of specific genes, promoters?

Three CLL patient subgroups can be distinguished by whole genome DNA methylation data (in our case 850k and 450k arrays). These groups are differentiated into *low*, *intermediate* and *high programmed* DNA methylation profiles and impact clinical outcomes (Oakes *et al*, 2016). We used these three categories as a covariate.

Line 228: To address the possible interplay of multiple genetic features, we applied linear regression with lasso regularisation to derive a multivariate predictor for each stimulus, composed of genetic, IGHV and DNA methylation covariates (Fig. 3B, Appendix Fig. 13).

12. With regard to tri12, in line 306 you mention that you performed differential expression and GSEA in positive vs negative cases. Did you perform this analysis separately for IGHV mutational status? This is unclear in the results text/figures. Going through the methods, it seems that you used 4 IGHV-M cases (clusters 3 and 4), but this has to be clarified in the results.
We thank the reviewer for pointing out this ambiguity in the methods. In response to Reviewer 1 we have removed the previous figure panels 4C and D, which showed a differential expression and GSEA analysis of trisomy 12 versus non-trisomy 12 CLL samples. Similar results have already been shown and thus we now refer to previous work in our lab (Dietrich *et al*, 2018; Herbst *et al*, 2022a) that showed that transcripts and proteins involved in BCR signalling are more abundant in trisomy 12. As a result we have removed the associated method for differential expression and GSEA of trisomy 12 CLL from the manuscript.

Moreover, comparing 2 vs 2 cases seems indeed insufficient for a discovery series.
We recognise that 2 vs 2 is a small sample size, and in the original manuscript we replicated our analysis with the data from Rendeiro et al (2016) (43 vs 9). When analysing the smaller 2 vs 2 dataset we calculated TF p-values in diffTF using the analytical approach rather than the permutation approach, to account for the smaller sample size.

Additionally, we repeated the analysis once more with the Beekman et al. (2018) dataset as the reviewer suggests, in which we compared 13 trisomy 12 samples with 87 non-trisomy 12 samples. This analysis validated our finding that Spi-B and PU.1 show higher inferred activity in trisomy 12, in a large and independent dataset.

In the case of the series by Rendeiro, this study analyzes 88 samples from 55 patients, and therefore, considering the sample size of your comparison (43 vs 9), I assume that you took tri12 cases vs WT regardless of their IGHV status. IGHV will be then a strong counfounding factor, as IGHV-M and -U show clearly different accessibility profiles and tri12 is actually enriched in IGHV-U cases.
The reviewer correctly highlights that we included both IGHV U and M samples in the diffTF analysis, and that IGHV status could be a strong confounding factor. We therefore ran diffTF using the design formula "~ sample_processing_batch + sex + IGHV status + trisomy 12" to account for the confounding effect of IGHV status. This is stated in the methods.

Line 637: We used diffTF in permutation mode (Berest et al, 2019) to infer the differential TF activity between trisomy 12 and non-trisomy 12 samples using TF motifs from HOCOMOCO v10 (Kulakovskiy et al, 2016). For the (Rendeiro et al, 2016) data, we used minOverlap = 2 and design formula: "~ sample_processing_batch + sex + IGHV status + trisomy 12" and for the (Beekman et al, 2018) data, we used minOverlap = 5 and design formula "~ sex + IGHV status + trisomy 12".

Additionally, I am aware of at least another series of CLL patients with available ATACseq data (Beekman et al., Nat Med 2018). It could potentially be also used for validation.

We repeated the analysis once more with the (Beekman et al. 2018) dataset as the reviewer suggested, in which we compared 13 trisomy 12 samples with 87 non-trisomy 12 samples. This analysis validated our finding that Spi-B and PU.1 show higher inferred activity in trisomy 12.

Line 264: We validated our finding in two additional independent datasets taken from Rendeiro et al, 2016 (nine trisomy 12 and 43 non-trisomy 12) and Beekman et al, 2018 (13 trisomy 12 and 87 non-trisomy 12). In the Rendeiro et al, 2016 data, nine TFs showed higher inferred activity (p<0.05) in the trisomy 12 samples (Fig. 4D) and in the Beekman et al, 2018 dataset, 18 TFs showed higher inferred activity (p<0.05) in the trisomy 12 samples (Appendix Fig. 15). Spi-B and PU.1 were the top hits in both datasets.

13. The potential role of SpiB in tri12 cases is interesting. However, the ChIPseq data are derived from DLBCL cell lines which have two important problems in your study: a) DLBCL has a clearly different biology than CLL, and b) cell lines and primary cases have different chromatin profiles. I am not sure how useful this dataset is in the context of your study. This limitation should be at least commented.

CLL Drug-microenvironment interactions MSB First Revision
We thank the reviewer for this comment and the interest in the finding involving Spi-B in trisomy 12. We agree with the reviewer that biological differences between cell lines and primary cells as well as between DLBCL and CLL make the direct interpretation of Spi-B binding sites difficult. To address this we have now added an over-representation analysis of Spi-B gene targets, defined using the CLL ATAC Seq data from Rendeiro et al. 2016. Thereby, we now show that Spi-B gene targets are enriched for pathways relating to microenvironmental signalling using both primary CLL ATACseq data (Appendix Tab. 6) and DLBCL cell line ChIPseq data (Appendix Tab. 7).

Line 281: To investigate this, we first visualised the genomic locations of all Spi-B motifs that showed differential inferred activity in trisomy 12 samples (Appendix Fig. 17). This analysis indicated that Spi-B binding motifs showing differential accessibility in trisomy 12 CLL are distributed throughout the genome. We next approximated Spi-B target genes in trisomy 12 CLL by annotating each of these motif locations with the nearest gene. We ran over-representation tests to investigate enrichment of immune and control pathways amongst these gene targets. We found that JAK-STAT signalling was enriched amongst these genes (Appendix Tab. 6).

To further validate this, we additionally determined Spi-B target genes through the use of a ChIPseq dataset (Care et al, 2014), quantifying Spi-B binding in lymphoma cell lines. We found that TLR, BCR and TGFβ signalling genes were enriched (p<0.01) amongst Spi-B targets based on ChIPseq data (Appendix Tab. 7). Whilst lymphoma cell lines cannot

completely capture the biology of CLL, this result supports the hypothesis that Spi-B may control microenvironmental response genes in malignant B cells.

Minor issues

14. You sometimes refer to genetic alterations as mutations. In general, SVs are not considered mutations, and this term is used more for gene-specific mutations. This is a bit misleading (e.g. Fig 3A bottom). Please use genetic alterations as a general term and mutations when referring to specific genes.

We have corrected the manuscript and figure legends to more accurately distinguish between specific mutations and genetic features in general.

15. The overall interpretation of the second part of the results section (lines 271-273) seems uncomplete and only biased towards the finding in M-CLLs.

We intentionally highlight the difference between the two predominantly IGHV mutated Clusters (C3 and C4) as we observed a distinct clinical and biological phenotype distinguishing these two. While we could identify two Clusters among IGHV unmutated patients which exhibited distinct microenvironmental response profiles, these Clusters did not show significantly different survival. The comparisons between C1 and C2 have been included in all figures where applicable and we have added Appendix Fig. 8 comparing C1 and C2 on a transcriptional level.

Line 212: Taken together, the patterns of responses to our set of stimuli distinguish two subgroups of IGHV-M CLL (C3 and C4) that are characterised by distinct in vivo pathway activities and differential disease progression.

Reviewer #3:

In this manuscript by Bruch et al. the authors investigate the impact of several microenvironmental stimuli on various drugs in a model of chronic lymphocytic leukemia (CLL). Using this system the authors identify 4 subgroups of CLL which respond differently to the chosen drugs and microenvironmental cues. The authors identify trisomy 12 as an important factor determining outcome dependent on microenvironmental signals and state that interleukin 4 and toll-like receptor stimulation may contribute to resistance mechanisms in CLL. The manuscript is well written, informative and of interest, **but it lacks significant validation in other, more biologically relevant model systems**. For instance, **the importance of adhesion to cells of the microenvironment (rather than mere exposure to soluble cytokines) or migration to microenvironmental components are completely disregarded.**

The cellular interaction between microenvironmental and malignant cells is very complex. With the approach presented in this manuscript we apply a reductionist model investigating many different combinations. This allows for direct conclusions between cause and effect of microenvironmental components on CLL cell viability and drug efficacy.

Regarding the cellular interaction between bone marrow stroma cells and malignant cells we have recently published a preprint (https://www.biorxiv.org/content/10.1101/2022.02.18.481065v1) which is currently being prepared for submission. These two manuscripts are based on two different datasets, apply different methods and try to answer distinct scientific questions. We therefore decided against merging these manuscripts to best approach the distinct readerships best suited for these studies.

Other concerns are:
1. How long were the cells cultured? Did the authors check that at the time of the experiments the cells still are phenotypically and functionally what they were at the beginning?
The cells were cultured for 48 hours from start of incubation to viability read-out. All CLL samples have been isolated from peripheral blood, stored on liquid nitrogen, thawed and taken into culture. This procedure has been previously established for CLL cells (Dietrich *et al*, 2018). In this short-term culture, no phenotypical change is expected in CLL cells.

We have added a description of these methods to the main text. As encouraged by the editorial policies, we added a step-by-step version to improve the reproducibility of our method.

Line 107: Relative viability was measured as luminescence-based ATP count (i.e. Celltiter Glo, Promega®) after 48h of drug-stimulus perturbation divided by the median of the DMSO controls. In subsequent analyses we used the natural logarithm of this value, so that negative and positive values indicate pro- and antiapoptotic effects, respectively, and values close to 0 correspond to no effect.

Line 473 (Methods): Mononuclear cells were isolated from peripheral blood of CLL patients by a Ficoll gradient and cryopreserved as previously described(Dietrich et al, 2018). Cells were incubated for 48 hours with or without drugs and stimuli. The viability was measured by a luminescent ATP readout (CellTiter-Glo, Promega ®). The detailed methodology is described below. (Followed by detailed step-by-step protocol)

2. How was the concentration of the microenvironmental stimuli determined?
CLL Drug-microenvironment interactions MSB First Revision
The stimuli were chosen based on a literature review of small scale *in vitro* studies of CLL. Concentrations were based on previously reported studies and on preliminary experiments.

3. At least one validating/more mechanistic experiment should be performed to test the subroups in biologically meaningful ways. Do these subgrops contain more or less leukemic stem cells?
Results: CLL Drug-microenvironment interactions MSB First Revision
Discussion: CLL Drug-microenvironment interactions MSB First Revision
In the original manuscript, we characterised the microenvironmental response, genetic features, DNA methylation status and RNA expression of each of the clusters. As an additional validation, we investigated the proteomic profiles of the clinically distinct Clusters 3 and 4 based on data previously published by us (Meier-Abt *et al*, 2021). Unfortunately, we could not identify differentially expressed proteins beyond a 10% false discovery rate.

We therefore investigated the clusters on a more functional level and compared their drug response profiles. As the comparisons between C1/2 and C3/4 are always biassed by the IGHV mutation status, for which a strong impact on drug response has previously been reported (Dietrich *et al*, 2018), we focused on the comparison between C1 and C2 as well as between C3 and C4.

We observed a higher efficacy of fludarabine and Nutlin-3a in C4 compared to C3. This difference is in line with the observation that TP53 mutations are depleted in C4. Surprisingly, the mTOR inhibitor everolimus showed increased efficacy in C4, which showed a lower mTOR activity in the GSEA compared to C3 (Fig. 2E). This points towards a reduced dependency on mTOR signalling in C3 due to increased baseline activity of this pathway.

The increased efficacy of NFkB inhibition with BAY-11-7085 in C3 points towards a higher baseline NFkB activity in C3. This is in line with the observation of a decreased effect of BAFF in C3 after NFkB inhibition and the GSEA presented in Fig. 2E.

Line 206: The clusters also demonstrated differential pathway sensitivities. For example, we found C3 to be more sensitive towards treatment with BAY-11-7085, a selective IκBα phosphorylation inhibitor (Appendix Fig. 9 & 10). In C3 samples the treatment with BAY-11-7085 led to a reduction of the pro-survival effect of BAFF, which stimulates the NFκB pathway (Appendix Fig. 11).

Line 416: This subgroup (C3) also demonstrated higher activity in vivo of pathways involved in response to microenvironmental signals, including NFκB, and was more susceptible to NFκB inhibition by BAY-11-7085.

For CLL, there is no established phenotype of leukemic stem cells, therefore a quantification of leukemic stem cells in our patient samples is not feasible. Since the vast majority of CLL cells are mature B-cells, a small fraction of cells with immature phenotype would not impact the measured viability which we present and use to define the clusters of microenvironmental response.

4. Figure 2C: In this reviewer's mind it is not sufficiently proven, that the differences in subgroups is due to the microenvironment.
We define the clusters based on responses to microenvironmental stimuli. The distinct microenvironmental response profiles are therefore a clear difference between those patient samples.

Whether the observed clinical differences between C3 and C4, namely the different TTT and LDT, are caused by microenvironmental signalling cannot be definitively proven based on our data. The multivariate cox-proportional hazards analysis shown in Appendix Tab. 4 however shows that the difference in disease progression is independent of the most important known biomarkers.

We further investigated the signalling activity of the two most important microenvironmental signalling pathways identified in our study in CLL infiltrated lymph nodes. There, we observed a faster disease progression of patients with higher IL4 signalling activity and saw the same trend in the TLR pathway.

5. Does group C3 consist of a group with genetic lesions previously known to be associated with poor response?
We thank the reviewer for highlighting this very important question. We describe the genetic alterations which are enriched or depleted in all clusters in Fig. 2D and have now updated this figure as suggested by reviewer 1 by adding the incidence of the reported genetic features. The only features significantly enriched in C3 are IGHV-M and POT1. IGHV mutated patients have a better clinical prognosis. POT1 is present in only 8% of the C3 patient samples and therefore cannot completely explain the worse prognosis of these patients. When taking into account the most important prognostic markers we still find a significant difference for TTT in Appendix Tab. 4.

Line 183: POT1 mutations were enriched in C3, which may contribute to the worse survival of C3 patients.

Line 192: Notably, the observed difference in progression dynamics in vivo was not explained solely by the known prognostic markers IGHV status, trisomy 12, TP53, DNA methylation profiles, and POT1. A multivariate Cox proportional hazards model accounting for these features showed an independent prognostic value of the cluster assignment between C3 and C4 ($p=0.03$, Appendix Tab. 5).

6. Suppl. Figure 5B: Why is there no difference in survival between C3 and C4?
Due to the high median age of onset of CLL, the indolent nature of the disease, and the many effective treatment options, CLL is no longer a leading cause of death, and therefore, prognostic differences only partly translate into overall survival. We chose Time to next treatment (TTT) as a biologically meaningful outcome measure which is independent of the available treatment options. It reflects the biological aggressiveness of the disease which ultimately leads to a treatment indication, including anaemia, thrombocytopenia or massive lymph node enlargement.

Line 162: Overall survival was not detectably different between C3 and C4 (Appendix Fig. 4B).

Standfirst text:

Combined perturbation by microenvironmental stimuli and drugs of chronic lymphocytic leukaemia cells annotated for genetic alterations reveals distinct response patterns and molecular modulators. Microenvironmental signalling activity *in vivo* was measured in primary lymph node samples.

- CLL samples fell into four subgroups with distinct progression dynamics by their microenvironmental response.
- Trisomy 12 enhances response to microenvironmental stimulation and has a distinct transcription factor activity profile which is inhibited by IBET-762 treatment.
- Linear modelling revealed different types of drug - stimuli interactions. The most common was drug resistance induced by microenvironmental stimulation.
- IL4 and TLR signalling is more active in CLL infiltrated lymph nodes, and higher IL4 signalling activity correlates with faster disease progression.

Thank you for sending us your revised manuscript. We have now heard back from the two reviewers who were asked to evaluate your revised study. As you will see below, the reviewers think that the study has improved as a result of the performed revisions. However, reviewer #1 raises a few remaining concerns, which we would ask you to address in a minor revision.

We would also ask you to address some remaining editorial issues listed below.

Reviewer #1:

Bruch/Giles et al. updated their manuscript "Drug-microenvironment perturbations reveal resistance mechanisms and prognostic subgroups in CLL" by completing methodological information, clarifying several passages in the paper, revisiting some statements, and providing additional data/analyses to strengthen their points. Validating their observations with primary published ATAC-seq data is appreciated and improves the manuscript. I still have comments:

• The pathway analysis is still not satisfying. In the appendix is the one picked immune pathway, and none of the pathways described in the point-to-point reply (Oxphos, p53 etc.). A ranked table would be useful. Why are the other pathways "control pathways"? Are they not playing any role here?
• Spi-B is always discussed together with PU.1 - similar binding sites, biological redundancy etc. Still, all analysis is centered solely around Spi-B. Why? Neglecting Pu.1 seems odd here, either provide a really good explanation for that or include it in the analysis /discussion / hypothesis- there is lots of PU.1 ChIP-seq data available, also it is differentially expressed and gene dosage was already shown to impact Pu.1 binding over the genome.
• The studies that tested stimuli (IFN etc.) on CLL cells should be mentioned in the introduction

Reviewer #3:

In this revised manuscript by Bruch et al. the authors have addressed most but not all of the reviewers' comments. The manuscript is improved, however, but still represents a reductionist approach to the microenvironment. This should be mentioned in the manuscript.

For the convenience of the reviewers, we have added our answers in blue.

Reviewer #1:

Bruch/Giles et al. updated their manuscript "Drug-microenvironment perturbations reveal resistance mechanisms and prognostic subgroups in CLL" by completing methodological information, clarifying several passages in the paper, revisiting some statements, and providing additional data/analyses to strengthen their points. Validating their observations with primary published ATAC-seq data is appreciated and improves the manuscript. I still have comments:

• The pathway analysis is still not satisfying. In the appendix is the one picked immune pathway, and none of the pathways described in the point-to-point reply (Oxphos, p53 etc.). A ranked table would be useful. Why are the other pathways "control pathways"? Are they not playing any role here?

We thank the reviewer for highlighting this point, and we have aimed to make this analysis easier to follow by updating table S5 and S6 to show ranked results. The tables now show all tested pathways that contain at least one Spi-B or PU.1 target gene. The pathways described in the main text are those with p value <0.01. Our hypothesis is that Spi-B and PU.1 control expression of microenvironmental signalling genes, and to that end we only test immune signalling pathways rather than all Hallmark or KEGG genesets. To ensure that immune pathways are specifically enriched, we included some control pathways (i.e. not related to immune signalling).

• Spi-B is always discussed together with PU.1 - similar binding sites, biological redundancy etc. Still, all analysis is centered solely around Spi-B. Why? Neglecting Pu.1 seems odd here, either provide a really good explanation for that or include it in the analysis /discussion / hypothesis- there is lots of PU.1 ChIP-seq data available, also it is differentially expressed and gene dosage was already shown to impact Pu.1 binding over the genome.

We obtained PU.1 ChIPseq data and updated our analysis of PU.1 target genes accordingly. PU.1 targets genes based on the ATACseq data were enriched for TCR signalling genes, whilst PU.1 targets based on ChIPseq data were not enriched for any immune pathways. We include these findings in the main text.

• The studies that tested stimuli (IFN etc.) on CLL cells should be mentioned in the introduction

We thank the reviewer for highlighting this. We have added the findings of Xia *et al.* to the introduction. As many studies have previously investigated individual microenvironmental factors in CLL, a complete overview of this literature in the introduction is not feasible. We cite multiple studies which have investigated individual components of the microenvironment and highlight two studies which also investigated the effects on drug toxicity.

Reviewer #3:

In this revised manuscript by Bruch et al. the authors have addressed most but not all of the reviewers' comments. The manuscript is improved, however, but still represents a reductionist approach to the microenvironment. This should be mentioned in the manuscript.

We agree with the reviewer that our study represents a reductionist approach to the microenvironment and as such is most suited to inform on individual pathway effects. We have elaborated on this point in the discussion and added it to the introduction.

Thank you again for sending us your revised manuscript. We have evaluated your manuscript and we think that the performed revisions have satisfactorily addressed the concerns of the reviewers. I am pleased to inform you that your paper has been accepted for publication.

EMBO PRESS

YOU MUST COMPLETE ALL CELLS WITH A PINK BACKGROUND

PLEASE NOTE THAT THIS CHECKLIST WILL BE PUBLISHED ALONGSIDE YOUR PAPER

Corresponding Author Name: Sascha Dietrich

Journal Submitted to: Molecular Systems Biology
Manuscript Number: MSB-2021-10855

USEFUL LINKS FOR COMPLETING THIS FORM

http://www.antibodypedia.com
http://1degreebio.org
http://www.equator-network.org/reporting-guidelines/improving-bioscience-research-repor
http://grants.nih.gov/grants/olaw/olaw.htm
http://www.mrc.ac.uk/Ourresearch/Ethicsresearchguidance/Useofanimals/index.htm
http://ClinicalTrials.gov
http://www.consort-statement.org
http://www.consort-statement.org/checklists/view/32-consort/66-title
http://www.equator-network.org/reporting-guidelines/reporting-recommendations-for-tumo
http://datadryad.org
http://figshare.com
http://www.ncbi.nlm.nih.gov/gap
http://www.ebi.ac.uk/ega
http://biomodels.net/
http://biomodels.net/miriam/
http://jjj.biochem.sun.ac.za
https://osp.od.nih.gov/biosafety-biosecurity-and-emerging-biotechnology/
http://www.selectagents.gov/

Reporting Checklist For Life Sciences Articles (Rev. June 2017)

This checklist is used to ensure good reporting standards and to improve the reproducibility of published results. These guidelines are consistent with the Principles and Guidelines for Reporting Preclinical Research issued by the NIH in 2014. Please follow the journal's authorship guidelines in preparing your manuscript.

A- Figures
1. Data
The data shown in figures should satisfy the following conditions:

- the data were obtained and processed according to the field's best practice and are presented to reflect the results of the experiments in an accurate and unbiased manner.
- figure panels include only data points, measurements or observations that can be compared to each other in a scientifically meaningful way.
- graphs include clearly labeled error bars for independent experiments and sample sizes. Unless justified, error bars should not be shown for technical replicates.
- if n< 5, the individual data points from each experiment should be plotted and any statistical test employed should be justified
- Source Data should be included to report the data underlying graphs. Please follow the guidelines set out in the author ship guidelines on Data Presentation.

2. Captions

Each figure caption should contain the following information, for each panel where they are relevant:

- a specification of the experimental system investigated (eg cell line, species name).
- the assay(s) and method(s) used to carry out the reported observations and measurements
- an explicit mention of the biological and chemical entity(ies) that are being measured.
- an explicit mention of the biological and chemical entity(ies) that are altered/varied/perturbed in a controlled manner.
- the exact sample size (n) for each experimental group/condition, given as a number, not a range;
- a description of the sample collection allowing the reader to understand whether the samples represent technical or biological replicates (including how many animals, litters, cultures, etc.).
- a statement of how many times the experiment shown was independently replicated in the laboratory.
- definitions of statistical methods and measures:
  - common tests, such as t-test (please specify whether paired vs. unpaired), simple χ2 tests, Wilcoxon and Mann-Whitney tests, can be unambiguously identified by name only, but more complex techniques should be described in the methods section;
  - are tests one-sided or two-sided?
  - are there adjustments for multiple comparisons?
  - exact statistical test results, e.g., P values = x but not P values < x;
  - definition of 'center values' as median or average;
  - definition of error bars as s.d. or s.e.m.

Any descriptions too long for the figure legend should be included in the methods section and/or with the source data.

In the pink boxes below, please ensure that the answers to the following questions are reported in the manuscript itself. Every question should be answered. If the question is not relevant to your research, please write NA (non applicable). We encourage you to include a specific subsection in the methods section for statistics, reagents, animal models and human subjects.

B- Statistics and general methods

Please fill out these boxes (Do not worry if you cannot see all your text once you press return)

1.a. How was the sample size chosen to ensure adequate power to detect a pre-specified effect size?	No effect size was pre-specified.
1.b. For animal studies, include a statement about sample size estimate even if no statistical methods were used.	NA
2. Describe inclusion/exclusion criteria if samples or animals were excluded from the analysis. Were the criteria pre-established?	Samples of CLL patients with available viable cells stored at the University Hospital Heidelberg were included. Samples which after thawing had less viable cells than necessary for the respective experiments were excluded.
3. Were any steps taken to minimize the effects of subjective bias when allocating animals/samples to treatment (e.g. randomization procedure)? If yes, please describe.	All selected samples were used for drug - stimuli profiling.
For animal studies, include a statement about randomization even if no randomization was used.	NA
4.a. Were any steps taken to minimize the effects of subjective bias during group allocation or/and when assessing results (e.g. blinding of the investigator)? If yes please describe.	Samples were measured in batches (12-18 patient samples each). The assignment to batches was performed randomly. The investigator was not blinded, as the samples needed to be collected, thawed and handeled.
4.b. For animal studies, include a statement about blinding even if no blinding was done	NA
5. For every figure, are statistical tests justified as appropriate?	Yes
Do the data meet the assumptions of the tests (e.g., normal distribution)? Describe any methods used to assess it.	Data of drug - stimuli perturbation experiments has been normalized to untreated control and log transformed prior to statistical testing. Thereby, normal distribution of this data has been ensured.
Is there an estimate of variation within each group of data?	Yes

Is the variance similar between the groups that are being statistically compared?	We detected no indication of unequal variance between the statistically compared groups.

C- Reagents

6. To show that antibodies were profiled for use in the system under study (assay and species), provide a citation, catalog number and/or clone number, supplementary information or reference to an antibody validation profile. e.g., Antibodypedia (see link list at top right), 1DegreeBio (see link list at top right).	N/A
7. Identify the source of cell lines and report if they were recently authenticated (e.g., by STR profiling) and tested for mycoplasma contamination.	N/A

* for all hyperlinks, please see the table at the top right of the document

D- Animal Models

8. Report species, strain, gender, age of animals and genetic modification status where applicable. Please detail housing and husbandry conditions and the source of animals.	N/A
9. For experiments involving live vertebrates, include a statement of compliance with ethical regulations and identify the committee(s) approving the experiments.	N/A
10. We recommend consulting the ARRIVE guidelines (see link list at top right) (PLoS Biol. 8(6), e1000412, 2010) to ensure that other relevant aspects of animal studies are adequately reported. See author guidelines, under 'Reporting Guidelines'. See also: NIH (see link list at top right) and MRC (see link list at top right) recommendations. Please confirm compliance.	N/A

E- Human Subjects

11. Identify the committee(s) approving the study protocol.	Ethics Committee of the medical faculty Heidelberg, Germany.
12. Include a statement confirming that informed consent was obtained from all subjects and that the experiments conformed to the principles set out in the WMA Declaration of Helsinki and the Department of Health and Human Services Belmont Report.	Informed consent was obtained from all patients before isolation and storage of CLL cells in line with the Declaration of Helsinki and the Department of Health and Human Services Belmont Report.
13. For publication of patient photos, include a statement confirming that consent to publish was obtained.	NA
14. Report any restrictions on the availability (and/or on the use) of human data or samples.	Drug - stimuli perturbation data is supplied in the GitHub Repository and stored at Biostudies. The ATAC Sequencing data is available at the EGA under the Data use Ontology DUO:0000006health/medical/biomedical research and clinical care. Meaning that the study of population origins or ancestry is not allowed.
15. Report the clinical trial registration number (at ClinicalTrials.gov or equivalent), where applicable.	NA
16. For phase II and III randomized controlled trials, please refer to the CONSORT flow diagram (see link list at top right) and submit the CONSORT checklist (see link list at top right) with your submission. See author guidelines, under 'Reporting Guidelines'. Please confirm you have submitted this list.	NA
17. For tumor marker prognostic studies, we recommend that you follow the REMARK reporting guidelines (see link list at top right). See author guidelines, under 'Reporting Guidelines'. Please confirm you have followed these guidelines.	NA

F- Data Accessibility

18: Provide a "Data Availability" section at the end of the Materials & Methods, listing the accession codes for data generated in this study and deposited in a public database (e.g. RNA-Seq data: Gene Expression Omnibus GSE39462, Proteomics data: PRIDE PXD000208 etc.) Please refer to our author guidelines for 'Data Deposition'.<br><br>Data deposition in a public repository is mandatory for:<br>a. Protein, DNA and RNA sequences<br>b. Macromolecular structures<br>c. Crystallographic data for small molecules<br>d. Functional genomics data<br>e. Proteomics and molecular interactions	The section is included in our manuscript: Screening data and patient annotation used in this study are available at github.com/Huber-group-EMBL/CLLCytokineScreen2021. The drug-stimuli-perturbation data and immunohistochemistry results have further been uploaded to BioStudies(Sarkans et al. 2018) under accession number S-BSST824. Original sequencing data has been uploaded to EGA under EGAD00001008723.
19. Deposition is strongly recommended for any datasets that are central and integral to the study; please consider the journal's data policy. If no structured public repository exists for a given data type, we encourage the provision of datasets in the manuscript as a Supplementary Document (see author guidelines under 'Expanded View' or in unstructured repositories such as Dryad (see link list at top right) or Figshare (see link list at top right).	All drug - stimuli perturbation data used in this analysis is available at https://github.com/Huber-group-EMBL/CLLCytokineScreen2021. Further, an online application is provided at dietrichlab.de/CLL_Microenvironment for rapid data exploration. To ensure long term data availability we have additionally uploaded the data to BioStudies(Sarkans et al. 2018) under accession number S-BSST824.
20. Access to human clinical and genomic datasets should be provided with as few restrictions as possible while respecting ethical obligations to the patients and relevant medical and legal issues. If practically possible and compatible with the individual consent agreement used in the study, such data should be deposited in one of the major public access-controlled repositories such as dbGAP (see link list at top right) or EGA (see link list at top right).	As described in the manuscript, the ATAC Seq data which is newly produced by us has been uploaded to EGA under EGAD00001008723. We obtained CLL ATACseq data(Rendeiro et al, 2016) from the EGA (EGAD00001002110) and ChIPseq data for Spi-B binding(Care et al, 2014) from the NCBI GEO database(Edgar et al, 2002) (GSE56857, GSM1370276).
21. Computational models that are central and integral to a study should be shared without restrictions and provided in a machine-readable form. The relevant accession numbers or links should be provided. When possible, standardized format (SBML, CellML) should be used instead of scripts (e.g. MATLAB). Authors are strongly encouraged to follow the MIRIAM guidelines (see link list at top right) and deposit their model in a public database such as Biomodels (see link list at top right) or JWS Online (see link list at top right). If computer source code is provided with the paper, it should be deposited in a public repository or included in supplementary information.	As described in the manuscript, the code for the analysis is provided in a reproducible form at https://github.com/Huber-group-EMBL/CLLCytokineScreen2021.

G- Dual use research of concern

22. Could your study fall under dual use research restrictions? Please check biosecurity documents (see link list at top right) and list of select agents and toxins (APHIS/CDC) (see link list at top right). According to our biosecurity guidelines, provide a statement only if it could.	N/A
